# Energy-Aware Finite-Horizon MPC Coordination of Adaptive VSG Inertia and BESS Control for Transient-Stability Enhancement in Renewable-Dominated Low-Inertia Hybrid Microgrids

**DOI:** 10.3390/s26175459

**Published:** 2026-08-28

**Authors:** Juan D. Rodríguez Romero, Emmanuel Hernández-Mayoral, Sergio A. Gamboa, V. Torres-García, Manuel Madrigal-Martínez, J. C. Trujillo-Caballero, O. A. Jaramillo

**Affiliations:** 1 Instituto de Energías Renovables, Universidad Nacional Autónoma de México (UNAM), Priv. Xochicalco s/n, Temixco 62580, Mexico; jdrr@ier.unam.mx (J.D.R.R.); sags@ier.unam.mx (S.A.G.); ojs@ier.unam.mx (O.A.J.); 2 SECIHTI, Instituto de Energías Renovables, Universidad Nacional Autónoma de México (UNAM), Priv. Xochicalco s/n, Temixco 62580, Mexico; 3 PGIIE, Instituto Nacional de Mexico, Morelia City 58120, Mexico; v.torres@ieee.org (V.T.-G.); manuelmadrigalmtz@gmail.com (M.M.-M.); 4 Instituto Nacional de Mexico, Orizaba City 58120, Mexico; juan.tc@orizaba.tecnm.mx

**Keywords:** renewable-dominated hybrid microgrid, low-inertia power system, adaptive virtual synchronous generator, finite-horizon model predictive control, battery energy-storage system, transient stability, RoCoF, frequency nadir, critical clearing time

## Abstract

The increasing integration of renewable energy sources (RESs) into modern power grids has reduced the rotational inertia traditionally provided by synchronous generators. This reduction poses significant challenges for transient stability, frequency regulation, and the dynamic resilience of power systems. The problem is more pronounced in renewable-dominated hybrid microgrids, where inverter-based resources are progressively replacing conventional generation units. In this context, sudden disturbances can produce large frequency deviations, high RoCoF values, oscillatory behavior, and even loss of stability. This paper proposes an energy-aware adaptive virtual synchronous generator (VSG) control strategy coordinated with a model predictive control (MPC)-based energy-management layer for a battery energy-storage system (BESS), in order to enhance the transient stability of a hybrid microgrid with high renewable penetration. Unlike conventional fixed-parameter VSGs, the proposed MPC–VSG–BESS framework dynamically updates active-power references and adjusts the virtual inertia *J* and damping *D* parameters in real time. In this way, the framework seeks to reduce RoCoF, improve the frequency nadir, and preserve the BESS operating constraints, including its energy availability. The methodology is evaluated using detailed EMT simulations in MATLAB–Simulink^®^ R2023b under severe contingencies, including three-phase faults and sudden loss of conventional generation, with renewable-penetration levels ranging from 70% to 100%. Under the most severe 100% RES condition, the proposed MPC–VSG–BESS framework limits the frequency nadir to 59.26 Hz compared with 56.78 Hz for the conventional control case, corresponding to a 77.0% reduction in the magnitude of the frequency nadir deviation. The proposed controller also extends the critical clearing time from 0 ms to 185 ms under the considered severe fault condition, while maintaining a maximum observed RoCoF of approximately 0.32 Hz/s. During the transient response, the virtual inertia and damping reach maximum observed values of 48.5 kg·m^2^ and 38.2 N·m·s/rad, respectively. Under a low initial SoC of 22%, the energy-aware constraint limits the admissible virtual inertia to 12.4 kg·m^2^. The MPC implementation requires an average computation time of approximately 2.8 ms and a maximum of 6.5 ms for a 10 ms control interval, demonstrating computational feasibility within the adopted simulation framework.

## 1. Introduction

### 1.1. Background and Motivation

The global transition toward decarbonized electric power systems has accelerated the deployment of renewable energy sources (RESs), particularly photovoltaic (PV) plants and wind-turbine (WT) generation, within distribution grids and microgrids. Although these resources reduce emissions and improve sustainability, their integration is mainly achieved through power converters rather than synchronous machines directly coupled to the grid. As a result, microgrids with high renewable penetration operate with lower physical inertia and lower electromechanical damping, which increases the speed and sensitivity of their dynamic response to disturbances.

Virtual synchronous generator (VSG)-based control has emerged as a promising strategy for grid-forming converters, as it enables emulation of synchronous machine swing dynamics through converter control. However, fixed-parameter VSG implementations may be suboptimal under variations in renewable generation, changes in BESS state of charge (SoC), and severe transient events. Therefore, a controller capable of increasing virtual inertia and damping during disturbances is required, without compromising the energy availability of the BESS once nominal operation is restored.

Hybrid microgrids provide a flexible architecture for integrating distributed energy resources (DERs), BESSs, dispatchable generation, and mixed loads within a controllable local grid. These architectures can improve reliability, resilience, and operational flexibility. Nevertheless, as conventional synchronous generation is displaced, their stability margins increasingly depend on the dynamic performance of grid-forming and grid-following converters [1,2,3].

Unlike conventional synchronous machines, inverter-based renewable generators do not inherently provide rotational inertia. Consequently, renewable-dominated microgrids exhibit low-inertia characteristics that increase their vulnerability to transient disturbances, frequency instability, oscillatory phenomena, and poor dynamic responses under abnormal operating conditions [4]. These effects become more severe as renewable-penetration approaches nearly fully renewable operation.

In grid-connected hybrid microgrids, severe transient events such as short-circuit faults, abrupt load variations, renewable intermittency, and disturbances originating from the transmission system can considerably reduce stability margins. Under these conditions, rapid frequency deviations and high RoCoF values may activate protections, increase oscillatory behavior, and in extreme cases lead to loss of system stability.

This work addresses that need by coordinating the adaptive inertia and damping of the VSG with a finite-horizon MPC-based BESS energy-management layer. The central hypothesis is that transient stability can be improved if the controller jointly considers frequency dynamics, RoCoF suppression, BESS power limits, converter saturation, and SoC-dependent energy availability [5,6].

Nevertheless, most conventional VSG strategies are designed with constant values of virtual inertia *J* and damping *D*. This condition limits their performance under dynamic operating variations, renewable-generation fluctuations, and energy constraints associated with the BESS SoC [7]. Likewise, the reported methods usually address virtual-inertia provision and BESS energy management separately, omitting their coordination during severe transient events [8,9].

### 1.2. Literature Review

Recent research on low-inertia microgrids can be grouped into four main families: fixed-parameter VSG control, heuristic or adaptive VSG tuning, MPC-based converter control, and BESS-oriented frequency-support strategies. Fixed VSG approaches improve inertial response compared with conventional grid-following control, but their performance depends strongly on preselected inertia and damping coefficients. Adaptive VSG methods improve flexibility, although many of them rely on heuristic rules and do not explicitly optimize future frequency trajectories or BESS energy constraints.

MPC-based approaches can predict system behavior over a finite horizon, but many formulations omit the electrochemical and converter limits of the storage resource. Finally, BESS-based frequency support provides fast power injection, but uncoordinated operation may overuse the battery during severe contingencies.

Therefore, the key unresolved problem is not whether the VSG, BESS, or MPC can individually improve stability, but rather how these three layers can be integrated into a single energy-aware predictive framework that enhances transient stability while respecting the BESS operating envelope. This manuscript seeks to strengthen the literature review by presenting a comparative table that distinguishes among fixed VSG, adaptive VSG, MPC-based VSG, BESS-only control, hybrid storage, and hardware-in-the-loop validations.

VSG-based control enhancements have become a research focus for new auxiliary modulation methods [10,11,12,13,14,15]. In [16], a comprehensive study is presented to explore the role of the BESS and the VSG in improving transient stability and frequency response in a low-inertia microgrid. In [17], the application of a BESS control scheme connected to a classical VSG is investigated, together with its impact on transient stability and primary frequency control in microgrids with high-RES penetration.

In [18], the authors analyzed problems associated with microgrid operation in both islanded and grid-connected modes, including large power-balance mismatches, frequency and voltage control, and microgrid instability caused by the simultaneous connection or disconnection of a large number of low-capacity RESs. In [19], the authors proposed adaptive BESS control to improve the transient stability and frequency response of a low-inertia microgrid. In [20], the impact of BESS location on transient stability and frequency support in microgrids with high renewable-energy penetration is presented. In [21], the authors discuss independent active- and reactive-power controllers by analyzing the VSG power-coupling mechanism in medium- and low-voltage microgrids, but high-voltage microgrids and transient-stability analysis are not addressed.

Furthermore, refs. [22,23] present adaptive-inertia techniques for the VSG with different control schemes, but without involving stability studies, only energy management. In [24,25], the authors use coordinated-control techniques for the classical VSG to provide J and thereby improve the transient and steady-state performance of the microgrid. In [26], it is discussed that the transient response of the system can also be improved by using a BESS with a VSG. However, stability support based only on BESSs can be inefficient and costly for high-voltage microgrids. Finally, in [27], the authors propose an auxiliary damping controller for a grid-connected VSG to minimize frequency deviation and improve system stability. However, the proposed technique is demonstrated in a single-machine infinite-bus scenario, while the results for a multi-machine scenario are left for future research.

According to the literature review, adaptive virtual inertia *J* schemes, intelligent-control techniques, and coordinated ESS management strategies have been proposed. Nevertheless, most studies focus on simplified test systems, islanded microgrids, or individual renewable technologies, without considering hybrid architectures with high renewable penetration interconnected to high-voltage transmission systems. Likewise, the integration of MPC schemes into adaptive VSG frameworks to coordinate the BESS and improve transient stability under ultrahigh renewable penetration remains limited.

#### Critical Comparison of Existing Approaches

The control strategies analyzed differ not only in their ability to modify virtual inertia and damping but also in how they consider the system’s future dynamics and energy availability. Fixed-parameter VSG schemes offer a relatively simple implementation and low computational load; however, their predefined *J* and *D* values can become inadequate as the penetration of renewable energies, operational conditions, and the severity of disturbances vary. Adaptive heuristic VSG strategies improve this flexibility by modifying the virtual coefficients based on instantaneous variables such as frequency deviation or RoCoF. Nonetheless, their decisions remain primarily reactive, as they do not explicitly optimize the system’s expected trajectory over a finite horizon. The review conducted in those manuscript identifies similar limitations in existing adaptive approaches, particularly regarding the treatment of BESS energy and SoC constraints.

MPC-based strategies overcome part of this limitation by explicitly optimizing the predicted trajectories of the system, subject to operational constraints. However, the reviewed literature indicates that MPC formulations are not necessarily linked to the electrochemical or converter constraints of the storage device. On the other hand, frequency-support strategies based on BESS can provide a rapid injection of active power, but uncoordinated operation may consume an excessive amount of stored energy during severe contingencies. Consequently, the difference between existing approaches and the proposed framework is not simply in the introduction of adaptive parameters *J* and *D*, but in the integration of the predictive adaptation of the VSG parameters with the future energy availability of the BESS within a single constrained optimization problem.

From a computational perspective, heuristic VSG schemes generally require less real-time computation, while finite-horizon MPC involves an additional optimization load. This dilemma becomes relevant in practical implementation, since the controller must solve the optimization problem within the sampling interval. In the proposed implementation, this aspect is explicitly evaluated: the quadratic programming formulation based on the active set requires approximately 2.8 ms on average—and less than 6.5 ms under the most demanding simulated conditions—compared to a control sampling period of 10 ms. Therefore, the proposed approach aims to preserve the predictive capabilities of MPC while maintaining computational feasibility for controlling rapid transients.

### 1.3. Research Gap

The literature demonstrates that VSG-type adaptive control can improve the dynamic response of low-inertia systems by modifying virtual inertia and damping based on instantaneous operating conditions. Therefore, the novelty of this work does not lie in the adaptive tuning of the *J* and *D* parameters themselves. Rather, the unresolved issue is how to predictively determine these parameters while simultaneously considering the future energy availability and operational constraints of the BESS. Current approaches to adaptive VSG typically determine virtual parameters from instantaneous or heuristic indicators. On the other hand, conventional MPC-based methods offer predictive optimization, but do not necessarily coordinate the resulting control action with the energy-dependent admissible region of the storage system, identifying the differences and characteristics in the summary in Table 1.

Similarly, BESS-based frequency-support strategies can provide rapid power injection, but may not explicitly consider the future energy consequences of such support when virtual inertia is simultaneously adapted. The proposed framework addresses this intersection by integrating the adaptive VSG parameters and active-power support of the BESS into a finite-horizon optimization problem whose feasible inertia region is explicitly conditioned on the BESS’s SoC and energy availability. This formulation allows the controller to balance improving transient stability with preserving the future capacity of the BESS, rather than maximizing instantaneous synthetic inertia regardless of the storage state.

To further specify the positioning of the proposed framework relative to existing approaches based on adaptive VSG and MPC, Table 2 presents a functional comparison of the main control paradigms identified in the literature. This comparison considers the ability to adapt virtual inertia and damping, the use of finite-horizon prediction, the explicit incorporation of the BESS charge state and energy constraints, the coordinated power support of the BESS, the consideration of future energy availability, applicability under high-renewable-energy penetration conditions, and the assessment of transient stability. Rather than claiming that the previous approaches lack individual control capabilities, the comparison aims to identify the specific combination of functionalities addressed by the proposed framework and clarify the distinction between adaptive parameter tuning and predictive coordination—subject to energy constraints—of VSG and BESS control.

As indicated in Table 2, the uniqueness of the proposed framework does not lie in the use of adaptive virtual inertia, MPC, or support through BESS as isolated technologies, since each of these functionalities has been the subject of previous studies. Instead, the proposed framework combines these elements through an explicit predictive energy-aware coordination mechanism. In particular, the BESS state is incorporated into the allowable operating region of the adaptive VSG, enabling the MPC to determine transient support actions while considering the available energy resource. This distinction becomes especially relevant in high and ultrahigh-renewable-energy penetration scenarios, where the demand for synthetic inertia increases, while the energy margin available for rapid support via BESS becomes progressively more restrictive.

### 1.4. Main Contributions

To address these gaps, this paper proposes an energy-aware finite-horizon MPC framework for adaptive VSG–BESS coordination in a renewable-dominated hybrid microgrid. The main contributions are:

- A finite-horizon MPC problem is formulated to coordinate BESS active-power support with transient-stability objectives, including improvement of the frequency nadir, reduction of RoCoF, and extension of the CCT.
- A dynamic hybrid-microgrid test bench is developed by integrating PV generation, wind generation, diesel generation, combined-cycle generation, ZIP load, BESS, and grid interconnection, evaluated for renewable-penetration levels from 10% to 100%.
- A set of severe contingencies is defined, including three-phase short-circuit faults and sudden disconnections of synchronous generation, in order to compare the proposed controller with base-case operation and fixed-parameter strategies.
- A sensitivity assessment is performed considering the SoC, BESS capacity, virtual-inertia limits, damping limits, and MPC horizon length. Action required by the authors: report the final numerical improvement percentages for CCT, RoCoF, frequency nadir, settling time, and BESS energy use.
- A comprehensive transient-stability assessment is carried out in a grid-connected hybrid microgrid composed of PV systems, WT wind turbines, diesel generators, combined-cycle units, and BESS.
- The proposed coordinated adaptive VSG–MPC framework significantly improves the CCT, reduces RoCoF, and optimizes frequency-nadir behavior compared with conventional fixed-parameter VSG strategies. The effectiveness of the approach is validated through detailed simulation scenarios implemented in MATLAB–Simulink^®^.

### 1.5. Paper Organization

The remainder of this paper is organized as follows. Section 2 describes the proposed hybrid microgrid and the operating scenarios. Section 3 presents the mathematical models of the RESs, synchronous generators (SGs), BESS, loads, and VSG dynamics. Section 4 formulates the energy-aware finite-horizon MPC controller. Section 5 defines the simulation framework, contingencies, and performance metrics. Section 6 presents the results and discussion. Section 7 summarizes the conclusions and future work.

## 2. Configuration and Operating Characteristics of the Proposed Hybrid Microgrid

A hybrid microgrid is a controllable local electric power system that integrates multiple DERs, ESSs, power converters, controllable and uncontrollable loads, measurement devices, and supervisory-control layers. It can operate connected to the main grid or in islanded mode, depending on technical and operational requirements. Electric microgrids are small-scale power systems consisting of RESs, ESSs, power converters, AC–DC buses, AC–DC loads, control units, system monitoring, and software interfaces [28].

### 2.1. Hybrid Microgrid Definition

**Figure 1:**
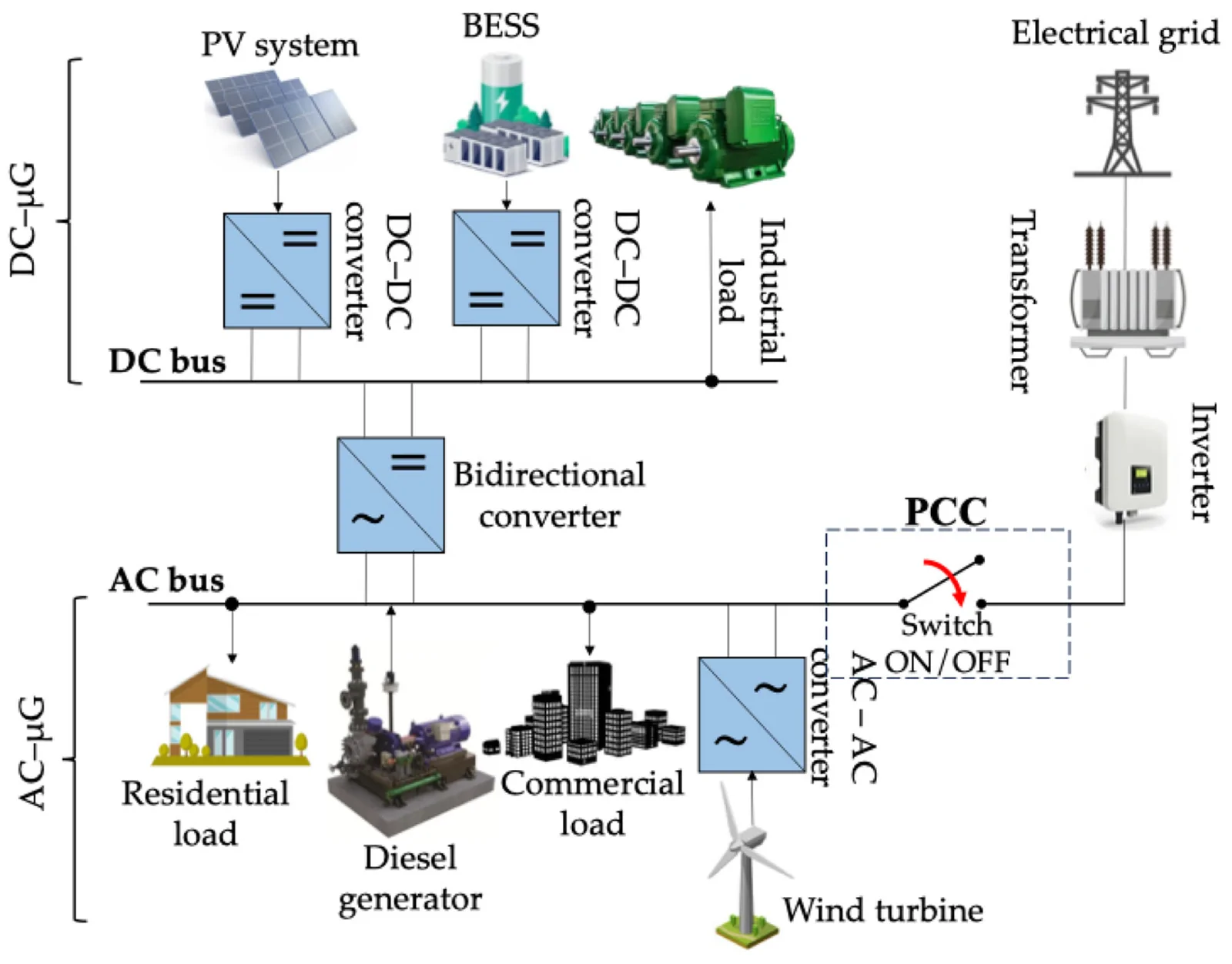
Architecture of a typical hybrid microgrid operating in both grid-connected and islanded modes.

Hybrid microgrids are intelligent electric power systems capable of integrating multiple DERs, ESSs, and different types of electrical loads within a controllable local grid. They can operate connected to the main grid or in islanded mode, ensuring reliable, resilient, and sustainable electricity supply. The architecture of a typical hybrid microgrid operating in grid-connected mode is shown in Figure 1.

### 2.2. Hybrid Microgrid Operational Characteristics

The proposed test system corresponds to a 10 MW grid-connected hybrid microgrid designed to represent the dynamic behavior of modern power systems with high participation of renewable sources and operation under low-inertia conditions. The microgrid integrates various DERs, including PV generation, WT generation, synchronous diesel generation, a gas-turbine-based combined-cycle generation unit, and BESS. The selected configuration represents a realistic transition scenario toward ultrahigh-renewable-penetration levels, close to operation based almost entirely on renewable sources. The inclusion of dispatchable conventional units, such as diesel and combined-cycle generators, provides operational controllability and auxiliary support under renewable-source intermittency and transient disturbances. Complementarily, the BESS and converter-interfaced RESs are coordinated through advanced control strategies to improve the transient-stability performance of the hybrid microgrid.

**Figure 2:**
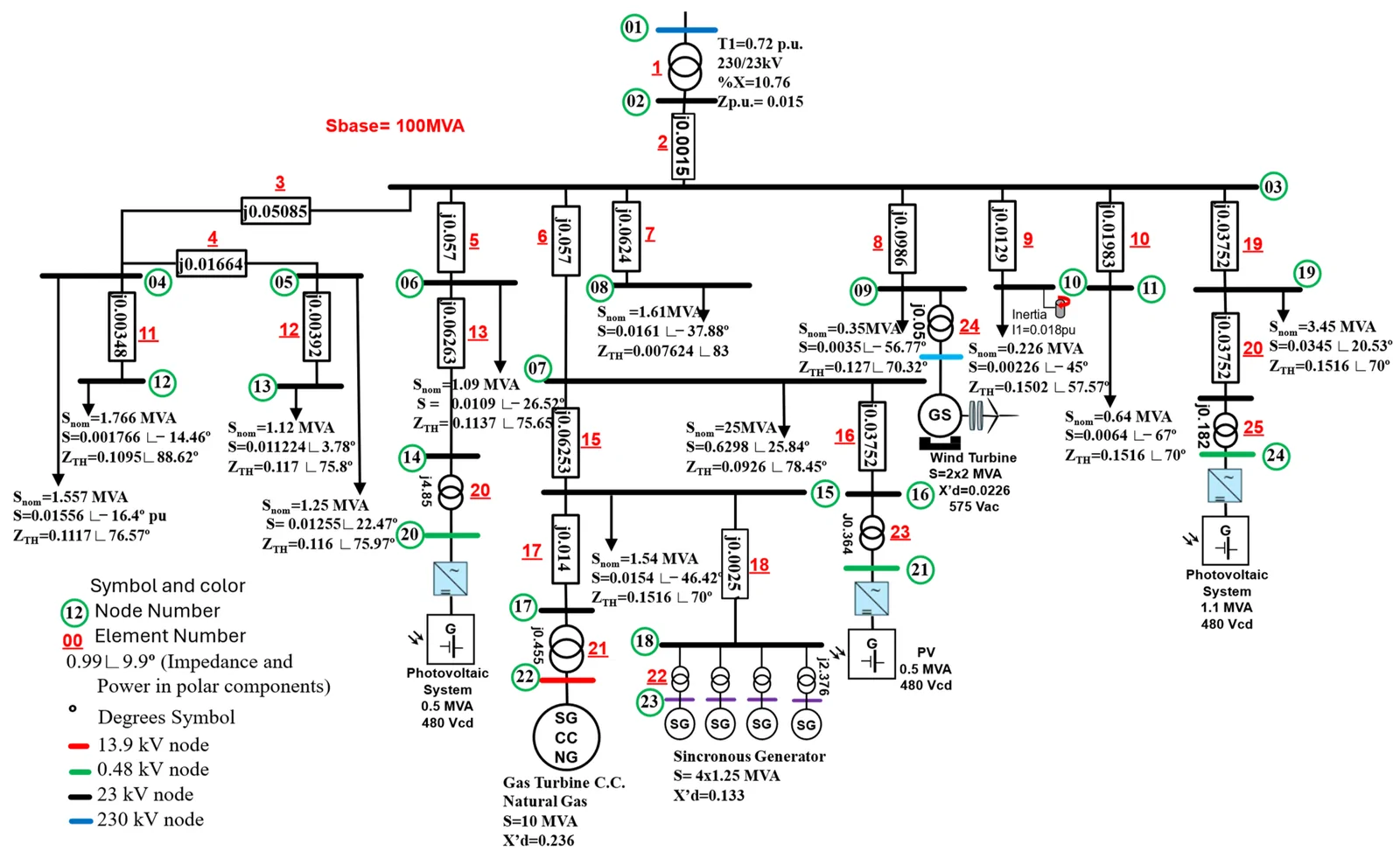
Electrical diagram of the proposed microgrid.

The hybrid microgrid is interconnected with the main electric grid through a point of common coupling (PCC), which operates at a voltage level of 13.8 kV, as shown in Figure 2. The total aggregated demand is 10.5 MW and is represented by a dynamic ZIP load model composed of industrial, commercial, and residential loads. The proposed architecture enables evaluation of transient-stability behavior under variable-inertia conditions, renewable-source intermittency, and severe disturbances, including three-phase short-circuit faults and sudden disconnection of SGs. Likewise, the hybrid microgrid adopts a radial medium-voltage distribution topology, in which all distributed generation units are connected to the main AC bus through dedicated step-up transformers and power-electronic interfaces.

It should be clarified that the nominal capacities of the components adopted in this study correspond to a design configuration oriented toward research, rather than a direct replica of a real electrical installation or an IEEE reference system. These capacities were selected to represent a medium-scale hybrid microgrid with a nominal load of 10.5 MW, while also providing a generation portfolio sufficiently heterogeneous to investigate the progressive replacement of synchronous inertia with renewable generation coupled through converters. The capacities of each microgrid component were deliberately chosen to allow controlled scalability of renewable penetration—from 10% up to 100%—while maintaining constant the grid structure, voltage level, load profile, and control architecture across all test cases. Therefore, the resulting configuration constitutes a reproducible test platform to investigate the behavior of transient stability under a progressive reduction of synchronous inertia.

The rated powers of the main components of the hybrid microgrid are summarized in Table 3. It should be noted that the different renewable-penetration levels are established by varying the active power supplied by the PV units and WTs, together with a proportional reduction in the participation of conventional SGs.

#### 2.2.1. PV Generation Systems

The PV system consists of a 2 MW utility-scale solar plant based on monocrystalline modules interconnected through a two-stage power-conversion topology. The first stage is a DC–DC boost converter, responsible for regulating the DC link and operating under a maximum power point tracking (MPPT) strategy using the perturb-and-observe (P&O) algorithm. The second stage consists of a three-phase voltage-source inverter (VSI), responsible for DC–AC conversion and controlled connection of the PV system to the hybrid microgrid. The PV inverter operates under a grid-following control strategy, with active- and reactive-power regulation capability. However, because of the dynamic decoupling introduced by the power-electronic interface, the direct inertial contribution of the PV system to the dynamic response of the hybrid microgrid is practically zero.

Therefore, high PV-generation penetration contributes to the reduction of the system’s effective inertia, which can increase the severity of the transient response to disturbances.

The PV system model implemented in MATLAB–Simulink^®^ considers irradiance variability, temperature effects, inverter switching dynamics, and DC-link voltage regulation in order to adequately represent its dynamic behavior under transient conditions.

#### 2.2.2. Wind Turbine

The wind-generation subsystem consists of two 2 MW variable-speed wind turbines. The first corresponds to a type III turbine based on a doubly fed induction generator (DFIG), while the second corresponds to a type IV turbine based on a permanent-magnet synchronous generator (PMSG) coupled to a full-scale power converter. These topologies allow decoupled control of active and reactive power and enable variable-speed operation to improve aerodynamic efficiency and wind-energy extraction.

Both wind turbines incorporate vector-control strategies for power-converter regulation, as well as blade pitch-angle control to limit the captured mechanical power under high-wind-speed conditions. As in the PV system, the direct inertial contribution of the wind turbines to the dynamics of the hybrid microgrid is limited because of the decoupling introduced by the power-electronic interfaces.

The aerodynamic model implemented for both turbines consider wind-speed variability, rotor mechanical dynamics, shaft-coupling effects, and converter switching dynamics. These elements make it possible to represent more accurately the transient behavior of the wind subsystem under grid disturbances and rapid variations in operating conditions.

#### 2.2.3. Diesel Generator Unit

The diesel-generation subsystem consists of a 1 MW medium-speed synchronous generator that operates as a dispatchable generation source within the hybrid microgrid. The unit includes a conventional frequency-governor system and an excitation-control system for voltage support.

The synchronous machine is modeled as a fourth-order dynamic machine, including transient electromagnetic dynamics, rotor swing equations, governor response, and excitation-system dynamics. An inertia constant (*H* = 4.5 s) is considered to represent the contribution of the diesel unit’s rotational inertia during transient disturbances. In addition, the diesel generator provides short-circuit current support, frequency-stabilization capability, and electromechanical damping *D* under low-renewable-energy penetration scenarios.

#### 2.2.4. Combined-Cycle Generation Unit

The combined-cycle generation subsystem consists of a 2 MW synchronous generation unit based on a gas turbine that operates as a base-load support source. The generator is modeled using a synchronous-machine representation that includes turbine-governor dynamics and IEEE type 1 excitation-system characteristics.

Compared with the diesel generator, the combined-cycle unit offers higher efficiency and a greater inertial contribution, represented by an inertia constant of *H* = 5.5 s. The unit contributes to voltage regulation, frequency support, and transient-stability enhancement during large disturbances. The gradual reduction in combined-cycle participation under ultrahigh-renewable-energy penetration scenarios enables assessment of low-inertia operating conditions in the proposed hybrid microgrid.

In this study, the combined-cycle unit is modeled exclusively as a dispatchable steam generator (SG). A mode of operation as a synchronous compensator is not considered when the unit is not dispatched for active-power generation. Such a move would require an explicit mechanical decoupling, operation of the excitation system, switching logic, and reactive power control—aspects that are outside the scope of this model. Consequently, the contributions to inertia and voltage support attributed to the CCGT unit are considered only while the SG remains in the modeled operating state.

#### 2.2.5. BESS

The BESS subsystem consists of a 1 MW/2 MWh lithium-ion battery connected through a bidirectional voltage-source converter. The BESS operates as the main fast frequency-support resource within the proposed adaptive-control framework. The BESS model includes SoC dynamics, charge–discharge efficiency, power-converter limitations, and operating constraints [29]. The SoC operating range is limited between 20% and 90% to preserve BESS lifetime and ensure operational reliability.

The BESS converter integrates the proposed adaptive VSG controller and the MPC-based energy-management strategy to provide synthetic inertia, fast active-power injection, transient damping *D*, and frequency-stabilization support during severe disturbances.

#### 2.2.6. Utility Grid Interconnection

The proposed electric grid adopts a radial distribution topology interconnected with the hybrid microgrid through a 13.8 kV medium-voltage distribution grid. The grid interconnection enables bidirectional energy exchange, provides voltage and frequency references during normal operation, and allows evaluation of transient interactions between the hybrid microgrid and the main electric grid under different renewable-energy penetration levels and disturbance conditions.

Each distributed-generation subsystem is connected through dedicated step-up transformers and feeders modeled by equivalent distributed-parameter (π) transmission-line representations. Table 4 presents the parameters of the electrical infrastructure described.

#### 2.2.7. Load Modeling

The local load is modeled using an aggregated dynamic ZIP representation with a total nominal demand of 10.5 MW. This formulation includes voltage-dependent components associated with constant impedance, constant current, and constant power, allowing the transient response of the microgrid to electrical faults, voltage deviations, and frequency disturbances to be captured more accurately. The aggregated load also considers a combination of industrial, commercial, and residential profiles to represent operating conditions closer to a realistic consumption scenario.

### 2.3. Renewable Penetration and Low-Inertia Operational Scenarios

To comprehensively evaluate transient-stability performance under low-inertia conditions, this work considers seven renewable-penetration scenarios, corresponding to renewable-generation contributions of 10%, 20%, 30%, 50%, 70%, 90%, and 100%. In the low-renewable-penetration scenarios, conventional SGs remain the main sources of physical inertia and primary frequency regulation.

However, as the participation of inverter-based renewable sources increases, the effective inertia of the system decreases because of the progressive displacement of conventional synchronous generation. In particular, for renewable-penetration levels of 90% and 100%, the hybrid microgrid relies more heavily on converter-based control strategies and on the energy-support mechanisms provided by BESSs to preserve transient stability.

The considered scenarios allow the proposed coordinated adaptive VSG–MPC control framework to be evaluated under progressively more demanding operating conditions characterized by lower equivalent inertia, higher sensitivity to disturbances, and greater dependence on the dynamic support provided by power-electronic-based resources.

As renewable-energy penetration increases, defined as:

(1) $$\mathrm{Renewable}\;\mathrm{Penetration}\;\left(\%\right)\;=\;\left(\frac{\mathrm{Total}\;\mathrm{Renewable}\;\mathrm{Energy}\;\mathrm{Generated}\;(\mathrm{MWh})}{\mathrm{Total}\;\mathrm{Electricity}\;\mathrm{Demand}/\mathrm{Load}\;(\mathrm{MWh})}\right)\;\times \;100$$

the participation of synchronous generation units decreases proportionally, resulting in a significant reduction in the equivalent rotational inertia of the hybrid microgrid. The system equivalent inertia is calculated as:

(2) $$H_{\mathrm{sys}}=\frac{\sum_{i=1}^{n}H_{\mathrm{SG},i}\;S_{\mathrm{SG},i}+\sum_{j=1}^{m}H_{\mathrm{VIR},j}{\;S}_{\mathrm{RES},j}}{S_{\mathrm{sys}}}$$

where:

$H_{\mathrm{sys}}$ is the equivalent inertia constant of the total system (in seconds).
$H_{\mathrm{SG},i}$ is the inertia constant of the *i*-th synchronous generator.
$S_{\mathrm{SG},i}$ is the rated capacity of the *i*-th synchronous generator.
$H_{\mathrm{VIR},j}$ is the simulated (virtual) inertia constant of the *j*-th renewable energy source/inverter.
$S_{\mathrm{RES},j}$ is the rated capacity of the *j*-th renewable energy source.
$S_{\mathrm{sys}}$ is the total system base capacity (sum of all synchronous generator and RES capacities).

Equation (2) represents exclusively the physical inertia of the synchronous-generation fleet before the activation of converter-based synthetic inertia. The adaptive VSG contribution should therefore not be interpreted as an additional physical inertia constant. Instead, $H_{\mathrm{VSG}}(t)$ represents a dynamic control-dependent contribution produced through BESS active-power modulation during transient events. Its activation and magnitude depend on the operating condition, the MPC decision, and the BESS/converter constraints.

Under ultrahigh-renewable-penetration conditions, the hybrid microgrid tends to operate with reduced transient-stability margins, high RoCoF values, shorter CCTs, faster frequency responses, and greater susceptibility to disturbances. Therefore, advanced synthetic-inertia and predictive energy-management strategies must be incorporated to reinforce dynamic stability and ensure safe operation under low-inertia conditions.

With regard to severe disturbance scenarios and stability-assessment conditions, two case studies are considered to evaluate the transient-stability robustness of the proposed control strategy. The first disturbance corresponds to a three-phase short circuit applied to the main distribution bus of the hybrid microgrid. The fault duration is progressively varied to determine the CCT under different renewable-energy penetration conditions.

This scenario evaluates the ability of the proposed control framework to maintain synchronism and frequency stability during severe voltage-depression conditions. The second disturbance scenario involves the sudden disconnection of the main synchronous generation unit, producing an abrupt power imbalance and a significant frequency deviation. This contingency is used to evaluate the dynamic frequency-support capability of the adaptive VSG controller and the coordinated response of the BESS.

The transient-stability performance of the proposed hybrid microgrid is evaluated using three main performance indicators: CCT, frequency nadir, and RoCoF. These metrics provide a quantitative assessment of system resilience, transient frequency behavior, and disturbance tolerance under renewable-dominated operating conditions.

Finally, regarding control and energy-management characteristics, the proposed operating framework integrates adaptive VSG control with MPC-based BESS energy management to improve transient stability under diverse operating conditions. The adaptive VSG controller dynamically modifies the *J* and *D* parameters according to the system frequency deviation, RoCoF, and BESS state of charge.

This adaptive mechanism enables greater inertial support during severe disturbances while preventing excessive BESS discharge. Complementarily, the MPC-based EMS optimizes BESS charging and discharging actions over a finite prediction horizon while complying with operating constraints, including SoC limits, BESS power limits, and frequency-support requirements. The coordinated interaction between the adaptive VSG and the MPC framework enables a fast dynamic response and greater transient resilience under high-renewable-energy penetration scenarios.

The proposed control architecture is implemented as a multi-rate hierarchical structure. The EMT electrical plant is simulated with a 50 μs integration step, the local AEA-VSG controller is updated at 1 ms, and the supervisory MPC is executed every 10 ms. The local AEA-VSG layer provides fast frequency/RoCoF response, whereas the supervisory MPC coordinates BESS active-power support and updates the allowable virtual-inertia and damping trajectories. The present EMT implementation assumes synchronized measurements and does not explicitly model communication latency, packet loss, or asynchronous distributed control. Therefore, the reported results should be interpreted as controller/plant results under ideal measurement and communication conditions. Communication-delay and sensor-noise robustness will require dedicated real-time or HIL validation.

### 2.4. Low-Inertia Dynamic Characteristics

The transition toward inverter-dominated operation fundamentally modifies the dynamic-response characteristics of the hybrid microgrid. Under conventional synchronous operation, transient disturbances are naturally mitigated by the kinetic energy stored in rotating masses. However, inverter-based renewable systems decouple mechanical dynamics from the electric grid through power converters, substantially reducing inertial-response capability.

The swing dynamics of conventional synchronous machines are represented by:

(3) $$\frac{2H}{\omega_{s}}\;\frac{d^{2}\delta }{{\mathrm{dt}}^{2}}=P_{m}-P_{e}$$

where:

H is the inertia constant (measured in MW-s/MVA or seconds).
$\omega_{s}$ is the synchronous angular velocity (in electrical rad/s).
δ is the rotor (or power) angle (in electrical radians or degrees).
$P_{m}$ is the mechanical input power (per unit).
$P_{e}$ is the electrical output power (per unit).

Under high-renewable-energy penetration conditions, the reduction in *H* increases frequency acceleration during disturbances, leading to high RoCoF values and reduced transient-stability margins. The proposed adaptive VSG controller compensates for this inertia deficit by emulating synthetic inertia integrated into the BESS converter architecture [30].

**Figure 3:**
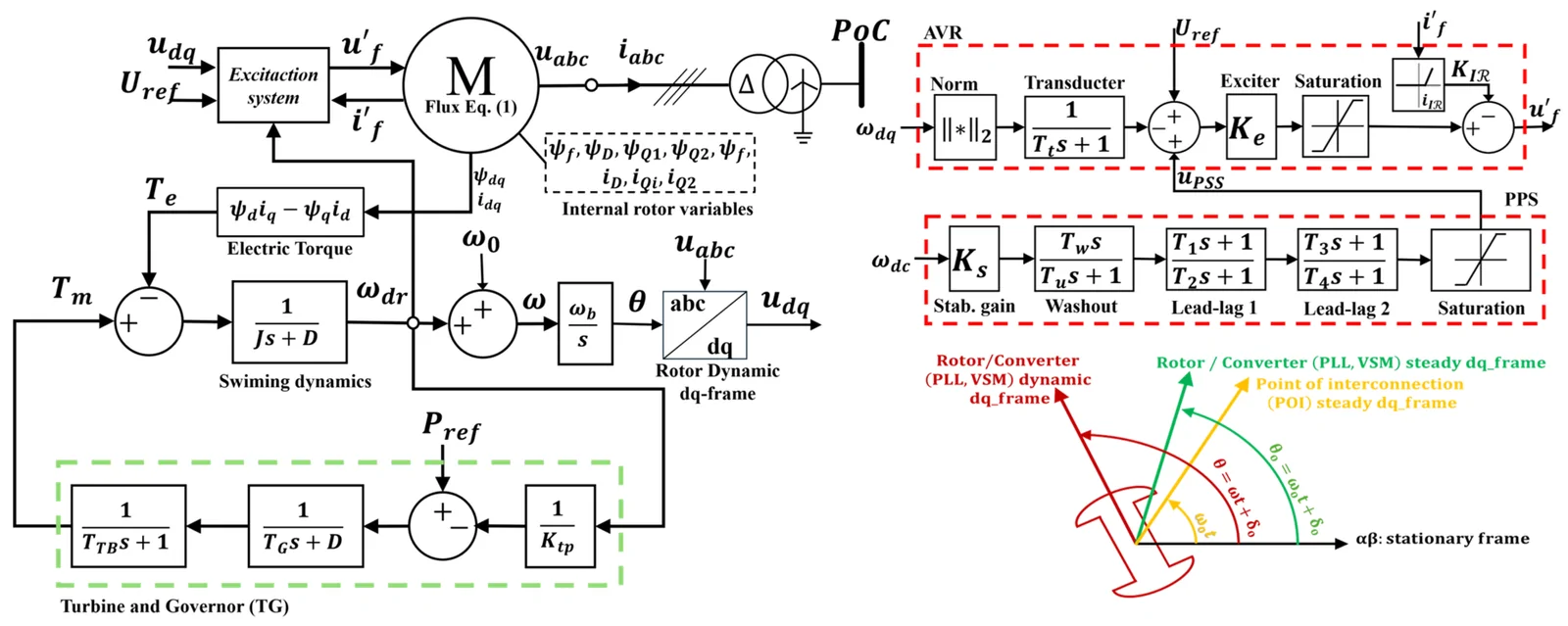
Dynamic transition from synchronous-dominated to converter-dominated operation.

The dynamic-transition diagram shown in Figure 3 illustrates the progressive reduction in physical inertia and the increasing dependence on synthetic-inertia support mechanisms as renewable-energy penetration approaches 100%. Synchronous condensers and converter-based synthetic inertia deliver complementary grid support functions and should not be regarded as interchangeable. Table 3 outlines their primary technical distinctions and the operational conditions under which each technology offers optimal advantages.

### 2.5. Sensing and Measurement Architecture

The proposed hybrid microgrid incorporates a measurement layer that provides the electrical and energy-state information required by the hierarchical control system. This layer establishes the interface between the physical electrical grid and the supervisory AEA-VSG–MPC controller. The measured variables are obtained from the PCC, converter interfaces, and BESS and are subsequently processed to generate the frequency, RoCoF, active-power, voltage, current, and SoC information required by the control algorithms (Table 5).

At the PCC, voltage and current measurements provide the electrical variables required to determine active and reactive power and to characterize the transient operating condition of the microgrid. The frequency signal is obtained from the measured electrical quantities and is used to calculate the frequency deviation Δ*f*. RoCoF is subsequently obtained from the temporal variation in the estimated frequency and is used as a transient-severity indicator by the adaptive VSG and supervisory MPC. At the BESS interface, electrical measurements provide the active-power response and operating-state information required to enforce the BESS power and energy constraints, while SoC provides the energy-availability information required by the energy-aware MPC formulation.

Accordingly, the measurement vector relevant to the proposed control architecture can be represented as:

(4) $$y_{m}\left(t\right)={\left[V_{\mathrm{PCC}},\;I_{\mathrm{PCC}},\;P_{\mathrm{PCC}},\;Q_{\mathrm{PCC}},\;f,\;\mathrm{RoCoF},\;P_{\mathrm{BESS}},\;\mathrm{SoC}\right]}^{T}$$

These measurements are processed according to the hierarchical timescales of the proposed controller. The electrical plant is resolved using the EMT integration step, while the local AEA-VSG controller operates with a 1 ms update interval and the supervisory MPC is executed every 10 ms. Thus, the sensing layer provides fast electrical information to the local controller and suitably processed measurements to the predictive layer.

The sensing layer is particularly relevant to the proposed energy-aware strategy because the controller simultaneously requires transient and energy-state information. Frequency and RoCoF characterize the severity and rate of the frequency disturbance, whereas BESS power and SoC determine the available support capability. Their joint use allows the MPC to distinguish between the need for immediate synthetic-inertia support and the requirement to preserve sufficient stored energy for subsequent operation (see Table 6).

### 2.6. Disturbance Scenarios and Transient-Stability Assessment Framework

The increasing integration of inverter-based renewable energy resources significantly modifies the transient behavior of modern microgrids because of the progressive reduction in physical *J* and electromechanical damping *D*. Under these conditions, severe disturbances can cause large frequency fluctuations, acceleration of rotor dynamics, reduced CCTs, and even loss of synchronism.

To comprehensively evaluate the effectiveness of the proposed adaptive coordinated VSG–MPC control strategy, a systematic transient-stability assessment framework is developed that considers multiple operating conditions, renewable-energy penetration levels, and disturbance severities.

The proposed assessment methodology aims to quantify the ability of the hybrid microgrid to withstand large disturbances while maintaining frequency stability and operational security. The hybrid microgrid is initially operated under the following nominal conditions (Table 7).

## 3. Mathematical Modeling of the Hybrid Microgrid

### 3.1. Dynamic Model of the Hybrid Microgrid

The proposed hybrid microgrid consists of multiple generation technologies with significantly different dynamic characteristics. Conventional SGs provide physical inertia and electromechanical damping, whereas PV and wind-generation systems are interconnected through power converters that by nature provide negligible rotational inertia.

Consequently, the overall dynamic behavior of the hybrid microgrid can be represented by the interaction among: (i) synchronous-generation dynamics, (ii) inverter-based renewable generation, (iii) BESS, (iv) electric-grid support, and (v) load variations. The active-power balance of a microgrid states that the total generated power must equal the power demanded by the loads plus system losses and the power exchange with the electric grid. This principle is expressed mathematically as:

(5) $$P_{\mathrm{PV}}\;+\;P_{\mathrm{WT}}\;+\;P_{\mathrm{DG}}\;+\;P_{\mathrm{CCGT}}{\;+\;P}_{\mathrm{BESS}}\;+\;P_{\mathrm{grid}}\;=\;P_{\mathrm{demand}}\;+\;P_{\mathrm{losses}}$$

where:

$P_{\mathrm{PV}}$ is the power generated by PV systems.
$P_{\mathrm{WT}}$ is the power generated by the wind systems.
$P_{\mathrm{DG}}$ is the power generated by the distributed generation sources.
$P_{\mathrm{CCGT}}$ is the combined-cycle power.
$P_{\mathrm{BESS}}$ is the BESS power.
$P_{\mathrm{grid}}$ is the grid power.
$P_{\mathrm{demand}}$ is the total load demand.
$P_{\mathrm{losses}}$ represent the system losses.

Any disturbance introduces a temporary power imbalance, given by:

(6) $$∆P\;=\;P_{\mathrm{PV}}\;+\;P_{\mathrm{WT}}\;+\;P_{\mathrm{DG}}\;+\;P_{\mathrm{CCGT}}\;+\;P_{\mathrm{grid}}-P_{\mathrm{demand}}$$

which directly affects the frequency dynamics of the system.

The detailed hybrid-microgrid model is formulated as a coupled nonlinear dynamic system in which the SGs, inverter-interfaced renewable sources, BESS, AC grid, PCC, and loads interact through the electrical grid equations and the active/reactive power balance. The system can be represented in compact form as:

(7) $$\dot{x}=f\left(x,\;u,\;w,\;p\right),$$

(8) $$0=g\left(x,\;u,\;w,\;p\right),$$

where x represents the dynamic states of the synchronous machines, converter-interfaced sources, BESS, and relevant load/grid states; u contains the control variables; w represents disturbances and external operating variations; and p contains the grid and component parameters.

At the system level, the active-power balance couples the individual subsystems according to (5), while deviations from the equilibrium condition generate the transient power imbalance represented by (6). This imbalance determines the frequency response through the synchronous-machine and converter dynamics. In parallel, the grid algebraic equations determine bus voltages, currents, and power flows.

The BESS-based AEA-VSG constitutes the principal interface between the physical plant and the supervisory MPC. Measurements of frequency, RoCoF, PCC voltage, BESS active power, and SoC are supplied to the MPC, which determines the admissible trajectories of PBESS, $J_{v}$, and $D_{v}$. These supervisory commands are subsequently executed by the local converter/VSG controller. Thus, the component models presented in the following subsections are interconnected subsystems of the same closed-loop EMT model rather than independent models.

### 3.2. Mathematical and Circuit Model of the PV System

**Figure 4:**
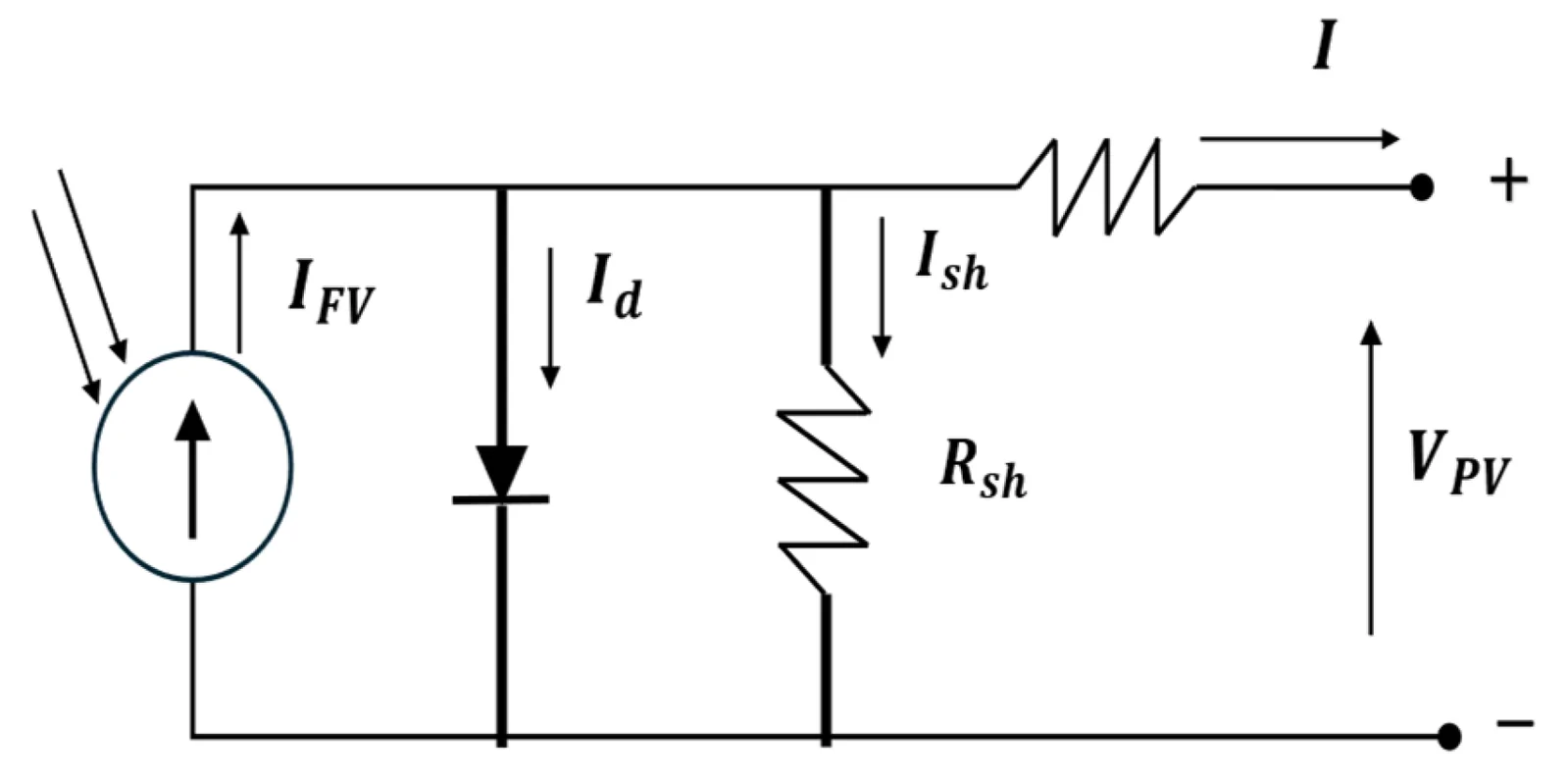
Equivalent circuit f PV cell: standard model IEEE.

A solar cell is a fundamental element of a PV system because it converts photonic energy into pollution-free electricity. A PV system consists of numerous PV cells connected in series and in parallel to produce a specific voltage and current at its output. Each solar cell can be represented as an electrical-circuit component containing a p–n junction known as a diode and a photocurrent generator that represents current generation from light. Several equivalent electronic circuits exist for a solar cell [31], as illustrated in Figure 4. By applying basic electrical-engineering techniques, such as Kirchhoff’s current law (KCL), and other methods detailed in [21], the output current of the PV cell, denoted I, is determined as follows:

(9) $$I=R_{\mathrm{sh}}-I_{0}\left[\exp\left(\frac{q\left(V+IR_{s}\right)}{\mathrm{akT}}\right)-1\right]-\frac{V+IR_{s}}{R_{\mathrm{sh}}}$$

where:

I is the subsystem output current.
$I_{\mathrm{PV}}$ is the photogenerated current (proportional to irradiance).
$I_{0}$ is the diode reverse saturation current (temperature-dependent).
q is the electron charge $\left(\approx 1.602\;\times \;10^{-19}\;C\right)$.
k is the Boltzmann constant $\left(\approx 1.381\;\times \;10^{-23}\;J/K\right)$.
T is the cell temperature (Kelvin).
a is the diode ideality factor (a value between 1 and 2).
$R_{s}$ is the series resistance (representing the ohmic resistance of the contacts and material).
$R_{\mathrm{sh}}$ is the parallel or shunt resistance (representing leakage currents).

The active power generated is:

(10) $$P_{\mathrm{PV}}=V_{\mathrm{PV}}\;I_{\mathrm{PV}}$$

The PV system operates through MPPT, maximizing energy extraction under variable irradiance conditions. A perturb-and-observe (P&O) algorithm is used as the direct control strategy for MPPT in unity power factor mode (PF = 1). Because the PV array is connected through a VSC, its inertial contribution is considered negligible.

This modeling choice is consistent with IEEE Std. 1547-2018, which specifies unity power factor as the default operating mode for distributed generation resources unless the grid operator requires otherwise. In addition, IEEE Std. 2800-2022 considers that inverter-based resources connected to transmission systems operate synchronously with the power system, implicitly depending on an existing voltage and frequency reference. The parameters of the PV system and power converter are shown in Table 8.

The PV system is composed of equivalent modules of approximately 540 W. Table 9 lists the PV-module parameters. Likewise, Table 10 shows the dynamic parameters of the PV-system power converter for transient-stability studies.

### 3.3. Mathematical and Circuit Model of the WTs

A wind turbine converts wind energy into mechanical energy and then into electrical energy through a generator. The active output power of the wind turbine and the input power to the generator depend only on changes in generator speed; therefore, output power can be regulated by adjusting generator speed to the desired level. The mechanical power captured by the wind turbine can be expressed by (10) [32].

(11) $$P_{W}=\frac{1}{2}\rho \;\pi \;R^{2}v^{3}C_{P}\left(\lambda ,\;\beta \right)$$

**Figure 5:**
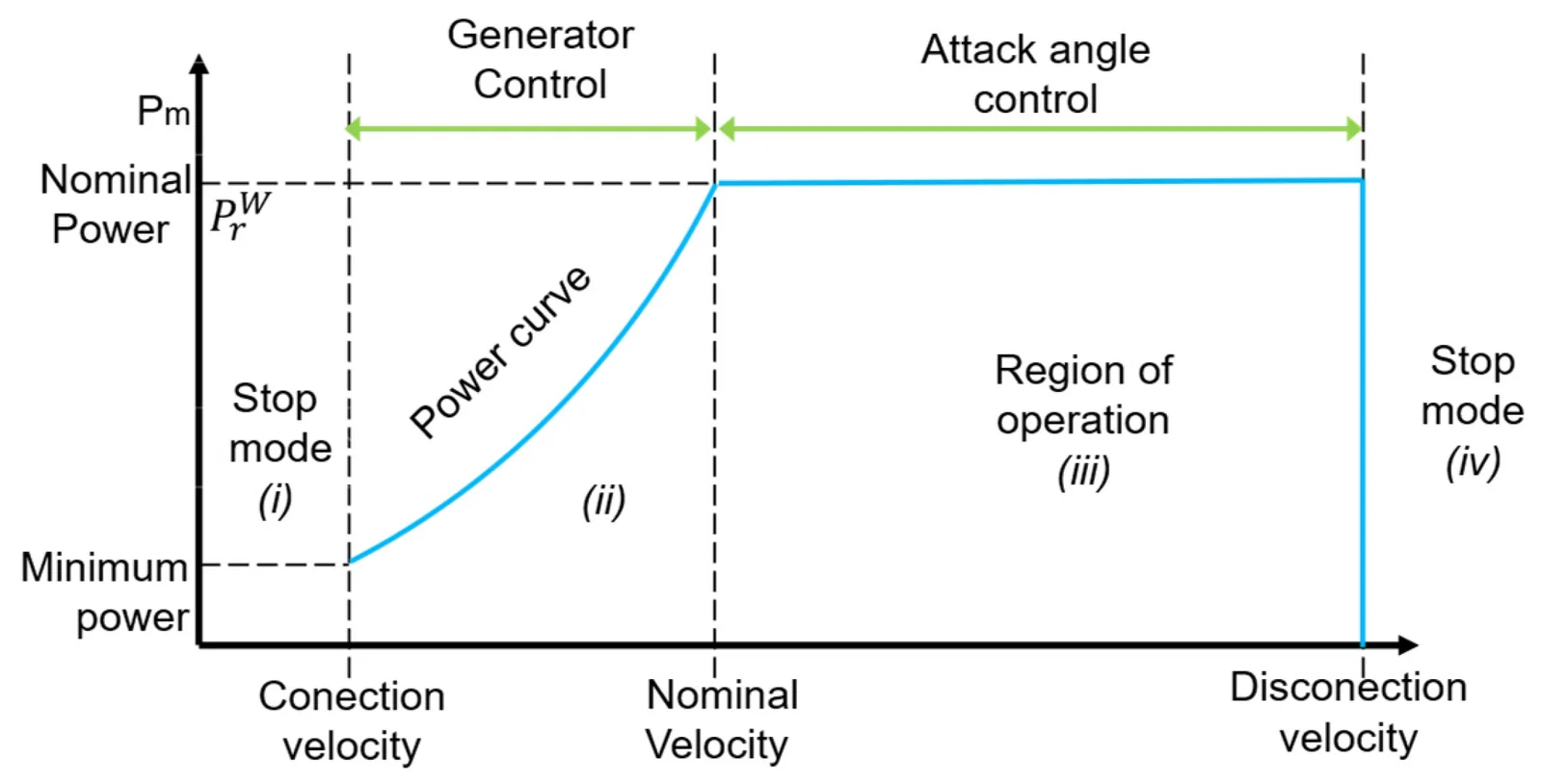
Wind-turbine performance at different speeds.

Here, the wind speed *v*^3^, blade radius *R*, blade angle *β*, air density ρ, and turbine power coefficient $C_{P}$ are functions of λ and *β*. Figure 5 shows the power-area diagram in terms of wind-turbine speed.

The three speeds (cut-in, rated, and cut-out) shown in Figure 5 determine different operating ranges of the system. The cut-in speed is the wind-speed region in which the turbine remains stopped (no power generation occurs).

The rated speed is the wind-speed region in which the turbine begins to rotate at rated speed, and once that value is reached, the power must remain constant in a steady state. In this speed range, increasing rotor angular speed would increase the generated power and the power system could become saturated upon reaching its maximum capacity. Finally, the cut-out speed is the speed at which the wind turbine is taken out of service to avoid damaging it and the angle of attack of the rotor blades is reduced until the blade is positioned parallel to the incident wind (0° angle of attack). The turbine stops rotating and the power is zero.

Table 11 and Table 12 summarize the technical parameters of the DFIG and PMSG wind turbines. Likewise, Table 13 summarizes the parameters of the dynamic control of the power converters of both wind turbines. The comparison of the dynamic characteristics of the DFIG and PMSG wind turbines is summarized in Table 14.

### 3.4. BESS Modeling

The BESS is the main fast-response energy resource within the proposed hybrid microgrid. Beyond its conventional energy-balancing function, the BESS serves as the physical platform for implementing the proposed energy-aware adaptive VSG strategy (BESS–VSG) and the MPC-based energy-management framework. Consequently, the BESS model must simultaneously represent: (i) ESS dynamics; (ii) electrical characteristics; (iii) SoC evolution; (iv) converter dynamics; (v) synthetic-inertia provision; and (vi) operating constraints. The adopted battery technology is a lithium-ion BESS with a nominal capacity of 1 MW/2 MWh, owing to its high energy density, fast dynamic response, high round-trip efficiency, and broad implementation in modern hybrid microgrids.

**Figure 6:**
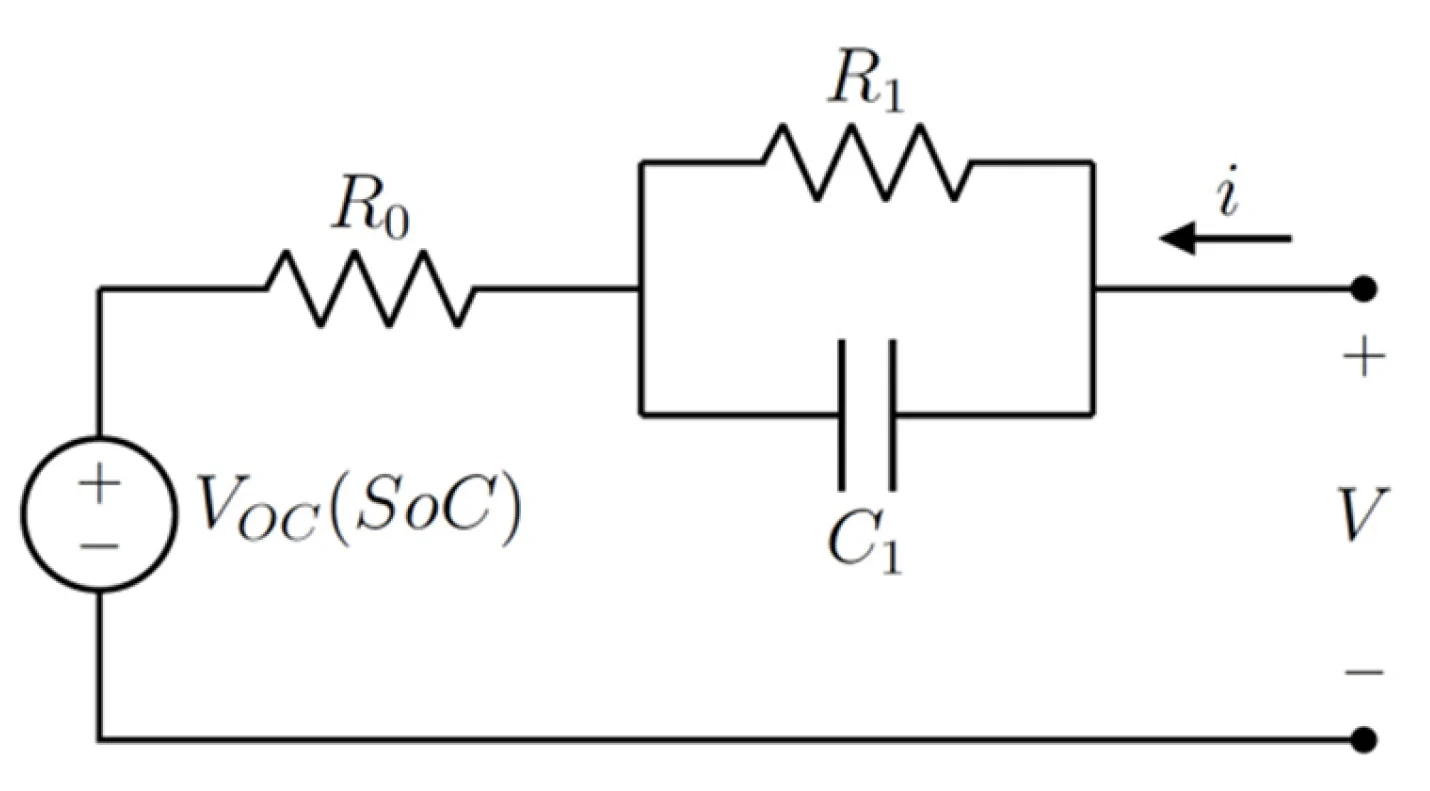
Diagram of the equivalent electrical circuit of a BESS.

The BESS is represented by a first-order Thevenin equivalent circuit, as shown in Figure 6, which provides an adequate balance between modeling accuracy and computational efficiency for transient-stability studies.

Here, $V_{\mathrm{oc}}$ is the open-circuit voltage source, $R_{0}$ is the internal resistance, $R_{1}$ is the polarization resistance, and $C_{1}$ is the polarization capacitance [28,29]. The voltage at the BESS terminals is expressed as:

(12) $$V_{\mathrm{BESS}\;}=V_{\mathrm{oc}}\left(\mathrm{SoC}\right)\;-\;I_{\mathrm{BESS}}R_{0}\;-\;V_{p}$$

where:

$V_{\mathrm{BESS}\;}$ is the instantaneous terminal voltage of the BESS (V).
$V_{\mathrm{oc}}\left(\mathrm{SoC}\right)$ is the open-circuit voltage (V), which exhibits a nonlinear dependence on the battery’s state of charge (SoC).
$I_{\mathrm{BESS}}$ is the internal BESS current (A), defined as positive during discharging and negative during charging.
$R_{0}$ represents the internal ohmic resistance $\left(\Omega \right)$, accounting for the instantaneous voltage droops.
$V_{p}$ is the polarization voltage (V), which models the transient overvoltage behaviors across the internal chemical diffusion layers.

The dynamics of the polarization voltage across the parallel RC grid are governed by the following first-order differential equation [30]:

(13) $$\frac{dV_{p}}{\mathrm{dt}}=-\frac{1}{R_{p}C_{p}}V_{p}+\frac{1}{C_{p}}I_{\mathrm{BESS}}$$

where $R_{p}$ and $C_{p}$ represent the polarization resistance $\left(\Omega \right)$ and polarization capacitance (*F*), respectively, dictating the short-term transient response of the storage medium.

To complete the mathematical formulation required for the controller’s energy-aware function, the instantaneous SoC tracker is modeled by Coulomb counting [33], as follows:

(14) $$\frac{\mathrm{dSoC}}{\mathrm{dt}}=-\frac{\eta }{Q_{n}}I_{\mathrm{BESS}}$$

where $Q_{n}$ is the nominal BESS capacity (Ah) and *η* is the Coulombic efficiency factor, with 0 < η ≤ 1. This model captures the transient voltage response during fast charging and discharging processes associated with frequency-support events. To avoid permanent degradation of the chemical cells, the state trajectory is limited by strict physical operating constraints [34]:

(15) $${\mathrm{SoC}}_{\min}\;\leq \;\mathrm{SoC}(t)\;\leq \;{\mathrm{SoC}}_{\max}$$

The net electrical power exchanged at the DC terminals of the BESS cells $P_{\mathrm{BESS}}$ is given by:

(16) $$P_{\mathrm{BESS}}\left(t\right)\;=\;V_{\mathrm{BESS}}\left(t\right)I_{\mathrm{BESS}}\left(t\right)\;=\;\left[V_{\mathrm{oc}}\left(\mathrm{SoC}\right)\;-\;I_{\mathrm{BESS}}\left(t\right)R_{0}\;-\;V_{p}\left(t\right)\right]I_{\mathrm{BESS}}\left(t\right)$$

The BESS model used in this study aims to reflect the relevant electrical and energy constraints necessary to maintain transient stability over short time horizons. Therefore, the controller sets clear limits on the SoC, active power, stored energy, current, voltage, and the rate of power change (ramp). These constraints ensure that the MPC does not consider the BESS as an unrestricted fast power source, maintaining that the synthetic inertia support is compatible with the instantaneous energy available from the storage device.

Electrochemical aging mechanisms, such as calendar aging and cycle aging, are not explicitly modeled because the perturbations studied in this work occur on a transient timescale ranging from milliseconds to a few seconds within a 60 s simulation horizon. In this short interval, the incremental capacity loss due to a single transient event is negligible compared to the long-term accumulated degradation caused by cycles, temperature, depth of discharge, and SoC history. Therefore, a detailed aging model would add a long-term timescale that is not relevant to the primary goal of the transient-stability analysis.

However, the repeated activation of the BESS for frequency support and synthetic inertia emulation would increase the accumulated energy flow and could contribute to long-term degradation of the BESS. In practical applications, this effect could be incorporated through an MPC formulation that accounts for aging, where the control objective includes a degradation cost based on the BESS energy flow, depth of discharge, or an electrochemical aging model. Such an extension would allow for a direct balance between improving transient stability and preserving the lifespan of the storage system, representing an important avenue for future work.

#### 3.4.1. Bidirectional Converter Model

The BESS is connected to the proposed 10 MW hybrid microgrid through a bidirectional buck–boost DC–DC converter coupled to a three-phase grid-forming VSI. If high-frequency switching harmonics are neglected, the average power dynamics of the electronic interface are represented as [35]:

(17) $$P_{\mathrm{AC}}\left(t\right)\;=\;\eta_{\mathrm{conv}}^{\mathrm{sign}\left(P_{\mathrm{BESS}}\right)}P_{\mathrm{BESS}}\left(t\right)$$

(18) $$\mathrm{sign}\left(P_{\mathrm{BESS}}\right)=\left\{\begin{array}{c} 1,\;\mathrm{if}\;P_{\mathrm{BESS}}\;\geq \;0\;(\mathrm{Discharging})\\ -1,\;\mathrm{if}\;P_{\mathrm{BESS}}\;<\;0\;(\mathrm{Charging}) \end{array}\right.$$

where $\eta_{\mathrm{conv}}$ represents the overall efficiency of the cascaded conversion stages. The active power injected into the AC bus of the hybrid microgrid $\left(P_{\mathrm{AC}}\right)$ is strictly limited by the thermal limits of the IGBTs.

(19) $${-P}_{\max}^{\mathrm{conv}}\;\leq \;P_{\mathrm{AC}}(t)\;\leq \;P_{\max}^{\mathrm{conv}}$$

#### 3.4.2. Synthetic Inertia Support Mechanism

To mitigate the severe frequency deviations caused by high penetration of variable renewable energy, the grid-forming VSI emulates the rotor dynamics of a traditional synchronous machine. The active-power reference for the converter is regulated by the virtual swing equation:

(20) $$P_{\mathrm{AC}}^{*}\left(t\right)\;=\;P_{\mathrm{ref}}\;-\;J\left(t\right)\omega_{0}\frac{d\omega \left(t\right)}{\mathrm{dt}}\;-\;D\left(t\right)\omega_{0}\left(\omega \left(t\right)\;-\;\omega_{0}\right)$$

where:

$P_{\mathrm{ref}}$ is the steady-state scheduled power dispatch.
$\omega \left(t\right)$ and $\omega_{0}$ are the measured and nominal virtual angular frequencies of the microgrid.
J(t) is the virtual moment of inertia $\left(\mathrm{kg}\cdot m^{2}\right)$.
D(t) is the virtual damping factor $\left(N\cdot m\cdot s/\mathrm{rad}\right)$.

Unlike conventional VSGs, the control parameters *J*(*t*) and *D*(*t*) are treated as dynamic control variables optimized online by the MPC framework [36].

#### 3.4.3. Energy-Aware Operation

The energy-management mechanism of the proposed framework is implemented by explicitly coupling the admissible contribution of virtual inertia to the instantaneous energy state of the BESS. Instead of imposing a direct dependence of the SoC on all the parameters of the VSG, the proposed formulation uses the BESS’s SoC to dynamically restrict the absolute inertia factor ($J_{n}$), which is one of the decision variables in the finite-horizon MPC. The corresponding upper limit is defined based on the energy-management relationship already implemented in the control model as:

(21) $$J_{n\text{\_}\max}\left(t\right)\;=\;J_{\mathrm{absolute}}\cdot \left[1-\exp\left(-\alpha \cdot \frac{\mathrm{SoC}\left(t\right)-{\mathrm{SoC}}_{\min}}{{\mathrm{SoC}}_{\max}-{\mathrm{SoC}}_{\min}}\right)\right]$$

(22) $$D_{n\text{\_}\max}\left(t\right)=D_{\mathrm{absolute}}\cdot \left[1-\exp\left(-\beta \;\cdot \frac{\mathrm{SoC}\left(t\right)-{\mathrm{SoC}}_{\min}}{{\mathrm{SoC}}_{\max}-{\mathrm{SoC}}_{\min}}\right)\right]$$

where α and β are tuning sensitivity coefficients, $J_{n}$ represents the maximum allowable normalized inertia factor, $\mathrm{SoC}\left(t\right)$ is the instantaneous load state of the BESS, and ${\mathrm{SoC}}_{\min}$ y ${\mathrm{SoC}}_{\max}$ define the permitted energy operating range. Consequently, a reduction in the available energy reserve progressively restricts the permissible contribution of synthetic inertia, preventing the MPC from requesting excessive inertial support when the BESS approaches its lower energy limit [37].

The inertia allocation is limited by the energy available in the BESS, since providing synthetic inertia requires a rapid exchange of active power. However, the damping adaptation remains as an independent dynamic degree of freedom to configure the transient response. The dimensional parameters of the VSG used in the converter are subsequently determined from the normalized MPC variables using the scaling relationships embedded in the model.

It is important to distinguish between the energy-dependent allowable limit and the instantaneous decision of the MPC. Equation (18) does not directly prescribe the value of $J_{n}$. Instead, it defines the maximum normalized inertia based on the available energy in the BESS. Subsequently, the MPC selects the optimal value (Jn^) within this feasible region, based on the predicted system dynamics and the associated control objectives. Therefore, the relationship ${J_{n\_max}}^{EA}=f(\mathrm{SoC})$ represents an energy-dependent constraint, while (Jn^) represents the actual optimized control action.

#### 3.4.4. State-Space Representation of the BESS

To implement the MPC algorithm, the combined electrochemical and electromechanical BESS equations are transformed into a unified nonlinear continuous-time state-space model:

(23) $$\dot{x}\left(t\right)\;=\;A\left(x\right)x\left(t\right)\;+\;B\left(x\right)u\left(t\right)\;+\;B_{d}d(t)$$

The state vector *x*(*t*), control input vector *u(t)*, and external disturbance vector d(t) are defined as:

(24) $$x\left(t\right)=\left[\begin{array}{c} \Delta \omega (t)\\ V_{p}(t)\\ SoC(t) \end{array}\right],\;u\left(t\right)=\left[\begin{array}{c} J(t)\\ D(t) \end{array}\right],\;d\left(t\right)=\left[\begin{array}{c} \Delta P_{L}(t)\\ \Delta P_{\mathrm{RES}}(t) \end{array}\right]$$

where $\Delta P_{L}(t)$ represents localized load steps and $\Delta P_{\mathrm{RES}}(t)$ accounts for rapid environmental power drops from the 10 MW generation portfolio. The system matrices are derived explicitly to allow the MPC to compute future trajectory horizons by linearizing the state-space model around each operational sampling point.

#### 3.4.5. Role of the BESS in Transient-Stability Enhancement

Under ultrahigh penetration of variable renewable energy (up to 100%), as in our study, the system lacks synchronous spinning reserve. When a severe fault occurs (a three-phase short circuit or disconnection of the main generator), the frequency of the proposed hybrid microgrid drops rapidly. Therefore, the role of the BESS in this mathematical model is threefold:

- First milliseconds (inertial response): The BESS instantaneously detects the RoCoF, and through the optimization of *J*(*t*) injects instantaneous power to arrest the frequency drop, directly improving the CCT.
- Transient phase (D support): As the frequency approaches its lowest point, the optimization of *D*(*t*) dominates the response, mitigating oscillatory behavior and stabilizing grid frequency within safe operating thresholds.
- Energy-constrained security: The mathematical integration of the SoC ensures that frequency-containment capability is maximized without activating low-voltage protections in the BESS cells.

Although BESS degradation is an important aspect of long-term energy-storage operation, the primary objective of this work was to evaluate transient-stability phenomena occurring over a timescale from milliseconds to a few seconds after severe disturbances. The investigated events, including three-phase short-circuit faults and generator outages, typically unfold over a period shorter than 20 s. In such short time intervals, electrochemical aging mechanisms—including solid-electrolyte interphase (SEI) growth, lithium plating, loss of active material, and electrolyte degradation—are negligible compared with the BESS lifetime, which is generally evaluated over thousands of charge–discharge cycles and several years of operation.

Consequently, degradation mechanisms are not explicitly incorporated into the dynamic model used in this study. Nevertheless, BESS-lifetime preservation is addressed indirectly through the proposed energy-aware control framework, which includes: (i) limiting BESS power fluctuations; (ii) restricting charge and discharge rates; (iii) enforcing SoC operating limits; and (iv) minimizing unnecessary energy expenditure through MPC optimization. Therefore, although electrochemical aging lies outside the scope of the present transient-stability analysis, the proposed control strategy intrinsically contributes to battery-lifetime preservation by avoiding excessive stress during frequency-support events. Table 15 summarize the BESS equivalent circuit parameters.

### 3.5. Diesel Generator Unit Modeling

The 1 MW diesel generator unit acts as a fast-response synchronous asset, providing both reference physical inertia and secondary frequency regulation. The unit is modeled by a salient-pole synchronous machine coupled to a speed governor and a diesel turbine.

The electromechanical behavior of the rotor is governed by the classical swing equation:

(25) $$2H_{d}\frac{d{\Delta \omega }_{d}\left(t\right)}{\mathrm{dt}}\;=\;P_{m}^{d}(t)-P_{e}^{d}(t)-D_{d}{\Delta \omega }_{d}\left(t\right)$$

where $H_{d}$ is the mechanical inertia constant (*s*) of the 1 MW unit; ${\Delta \omega }_{d}$ is the rotor speed deviation (p.u.); $P_{m}^{d}$ and $P_{e}^{d}$ are the mechanical power input and electromagnetic power output (p.u.); and $D_{d}$ is the physical damping coefficient.

The dynamics of the speed governor and fuel actuator, which regulate the fuel-injection mechanism as a function of frequency error, are expressed by a first-order transfer-function grid:

(26) $$\frac{d\phi_{d}\left(t\right)}{\mathrm{dt}}\;=\;\frac{1}{T_{g}}\;=\;\left[\frac{1}{R_{d}}\left(\omega_{\mathrm{ref}}-\omega_{d}\left(t\right)\right)-\phi_{d}\left(t\right)\right]$$

(27) $$\frac{dP_{m}^{d}\left(t\right)}{\mathrm{dt}}=\frac{1}{T_{d}}\left[\phi_{d}\left(t\right)-P_{m}^{d}\left(t\right)\right]$$

where $R_{d}$ represents the governor speed droop (%); $\phi_{d}\left(t\right)$ is the fuel valve position; $T_{g}$ is the governor time constant (*s*); and $T_{d}$ is the diesel engine combustion delay (*s*).

To ensure full reproducibility of the proposed hybrid microgrid and to ground the MATLAB–Simulink^®^ environment in validated power-system references, the exact design parameter values for the diesel generator are presented in Table 16.

### 3.6. Combined-Cycle Generation Unit Modeling

The 2 MW combined-cycle power plant (CCPP) has a multi-shaft configuration consisting of a gas turbine (GT), a heat-recovery steam generator (HRSG), and a steam turbine (ST). The CCPP provides substantial base-load power, but exhibits slower thermodynamic response times compared with the diesel unit.

The total mechanical power injected into the synchronous shaft $\left(P_{m}^{\mathrm{cc}}\right)$ e is the sum of the power generated by the gas and steam stages:

(28) $$P_{m}^{\mathrm{cc}}\left(t\right)\;=\;P_{\mathrm{GT}}\left(t\right)\;+\;P_{\mathrm{ST}}\left(t\right)$$

The dynamics of the GT governor and fuel system are modeled by considering the fuel-valve delay $\left(T_{f}\right)$ and the compressor discharge acceleration delay $\left(T_{c}\right)$:

(29) $$\frac{dX_{f}\left(t\right)}{\mathrm{dt}}=\frac{1}{T_{f}}\left[\frac{1}{R_{\mathrm{cc}}}\Delta \omega_{\mathrm{cc}}\left(t\right)-X_{f}\left(t\right)\right]$$

(30) $$P_{\mathrm{GT}}\left(t\right)=\frac{1}{1+sT_{c}}X_{f\;}\left(t\right)$$

where $R_{\mathrm{cc}}$ is the permanent drop of the combined-cycle plant and $X_{f}$ is the fuel command. The HRSG channels the GT exhaust gases to generate high-pressure steam. This thermodynamic delay and the resulting mechanical-power generation of the steam turbine $\left(P_{\mathrm{ST}}\right)$ are captured through a structural transport delay and a steam-chest time constant $\left(T_{s}t\right)$:

(31) $$\frac{dP_{\mathrm{ST}}\left(t\right)}{\mathrm{dt}}=\frac{1}{T_{\mathrm{st}}}\left[K_{\mathrm{HRSG}}P_{\mathrm{GT}}\left(t-T_{h}\right)-P_{\mathrm{ST}}\left(t\right)\right]$$

where $K_{\mathrm{HRSG}}$ is the thermal efficiency gain of the recovery boiler. The mechanical rotor dynamics match the synchronous machine swing equation with an operational inertia constant $H_{\mathrm{cc}}$ scaled for a 2 MW operating base.

Table 17 presents the exact design parameter values for the combined-cycle power plant (CCPP). These parameters structurally define the reference physical response of the hybrid microgrid. When renewable-energy penetration levels are adjusted during the simulations (10%, 20%, 30%, 50%, 70%, 90%, and 100%), the physical generation contribution of these units is dynamically reduced.

At 100% renewable-energy penetration, these arrays are electronically isolated, reducing the inherent inertia of the system to zero and forcing the proposed adaptive MPC–VSG–BESS framework to fully replace the dynamic-stability limits of the hybrid microgrid.

### 3.7. Load Modeling

In transient-stability evaluations, assuming a static constant-power load can generate overly conservative or inaccurate results. To capture the realistic physics of hybrid-microgrid demand during voltage sags and frequency deviations caused by faults, a dynamic ZIP model is implemented (constant impedance, constant current, constant power) integrated with frequency-dependence factors. The active power $\left(P_{\mathrm{active}}\right)$ and reactive power $\left(Q_{\mathrm{reactive}}\right)$ absorbed by the load bus are mathematically formulated as:

(32) $$P_{\mathrm{active}}\left(t\right)\;=\;P_{0}\left[a_{1}{\left(\frac{V(t)}{V_{0}}\right)}^{2}+\;a_{2}{\left(\frac{V(t)}{V_{0}}\right)}^{1}+\;a_{3}\right]\cdot \;\left[1\;+\;K_{\mathrm{pf}\;}\left(f\left(t\right)-f_{0}\right)\right]$$

(33) $$Q_{\mathrm{reactive}}\left(t\right)=Q_{0}\left[a_{4}{\left(\frac{V(t)}{V_{0}}\right)}^{2}+a_{5}{\left(\frac{V(t)}{V_{0}}\right)}^{1}+a_{6}\right]\cdot \left[1+K_{\mathrm{qf}}\left(f\left(t\right)-f_{0}\right)\right]$$

where:

$P_{0}$ and $Q_{0}$ are the nominal active and reactive power parameters at nominal voltage $V_{0}$ and nominal frequency $f_{0}$ (60 Hz).
V(t) and f(*t*) are the instantaneous bus voltage magnitude and frequency, respectively.
$K_{\mathrm{pf}}$ and $K_{\mathrm{qf}}$ are the frequency sensitivity coefficients of the load (%/Hz).

The dimensionless coefficients $a_{1}$ to $a_{6}$ define the proportional composition of the load type, satisfying the following algebraic unities:

(34) $$a_{1}+a_{2}+a_{3}=1$$

(35) $$a_{4}+a_{5}+a_{6}=1$$

Here, $a_{1}$ and $a_{4}$ represent the constant-impedance fractions (for example, resistive heating); $a_{2}$ and $a_{5}$ represent the constant-current fractions (for example, conventional lighting); and $a_{3}$ y $a_{6}$ represent the constant-power fractions (for example, electronically regulated industrial motor drives).

The distribution coefficients are explicitly defined to satisfy the algebraic units $\sum_{i=1}^{3}a_{i}=1$ and $\sum_{i=4}^{6}a_{i}=1$. During a three-phase short circuit or disconnection of the main generator, the local bus voltage drops significantly. Because 40% of the active load is modeled as constant impedance $\left(a_{1}\;=\;0.40\right)$, the net power consumed by the load automatically decreases in proportion to the voltage $\left(V/{V_{0}}^{2}\right)$.

This voltage-dependent load reduction provides an intrinsic self-healing power relief to the hybrid microgrid, modifying the virtual active-power injection required from the BESS converter. The inclusion of these parameters prevents the simulation from yielding overly pessimistic frequency-nadir and RoCoF values.

The values in Table 18 represent a realistic mixed commercial–industrial feeder profile, where the combination of static components and frequency-dependent behavior considerably influences the localized voltage profile of the hybrid microgrid and the frequency response during transient events.

## 4. MPC-Driven Adaptive Energy-Aware VSG Framework

In renewable-dominated microgrids, transient-stability requirements vary continuously because of (i) renewable-generation fluctuations, (ii) load-demand variation, (iii) BESS SoC limitations, (iv) grid disturbances, and (v) changes in system J levels. Therefore, a supervisory predictive-control layer is introduced to optimize VSG behavior in real time.

**Figure 7:**
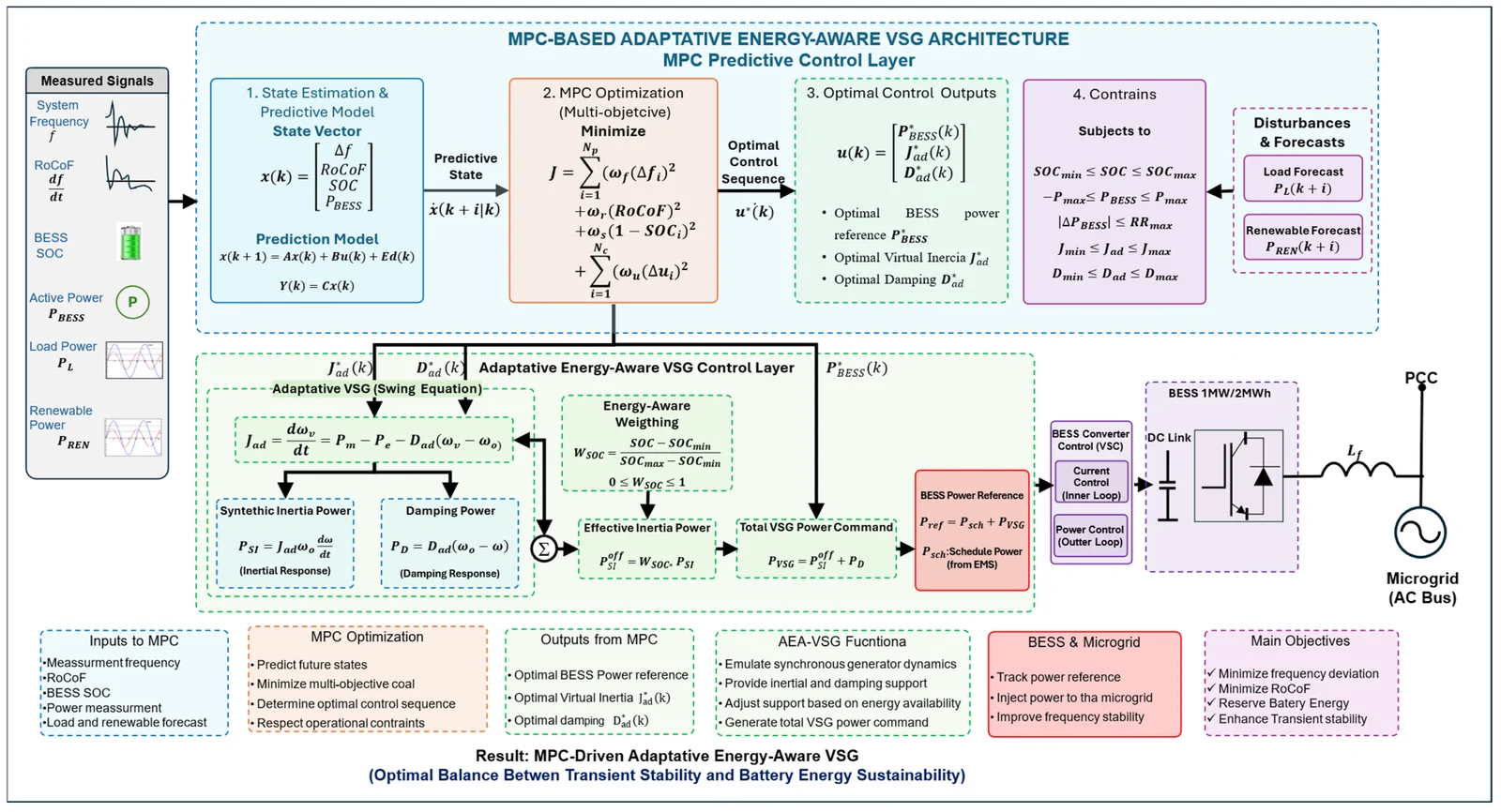
MPC-driven adaptive energy-aware VSG architecture.

To achieve this objective, a model predictive control (MPC) framework is integrated with the proposed energy-aware adaptive VSG (AEA-VSG), resulting in the MPC-based energy-aware adaptive VSG architecture illustrated in Figure 7.

The proposed controller continuously predicts the future system dynamics and determines the optimal values of active power, virtual inertia, and virtual damping, $P_{\mathrm{BESS}}$, $J_{n}$ y $D_{n}$, over a finite prediction horizon. A key feature of the proposed formulation is the asymmetric treatment of virtual inertia and damping. The normalized inertia factor ($J_{n}$) is explicitly linked to the available energy state of the BESS through SoC-dependent limits. In this way, the maximum admissible contribution of synthetic inertia is reduced when the available energy reserve is limited. Conversely, the normalized damping factor ($D_{n}$) is dynamically adjusted by the MPC within fixed lower and upper bounds without directly depending on the SoC.

This separation allows the controller to use damping adaptation primarily to shape the transient response, while the allocation of synthetic inertia is coordinated with the future energy availability of the BESS. Consequently, the energy-aware management feature of the proposed framework is incorporated through predictive coordination between the BESS energy availability and the admissible inertia contribution, rather than imposing an artificial SoC dependence on all VSG parameters.

Therefore, this section proposes an AEA-VSG control strategy embedded in the BESS. The proposed approach dynamically adjusts the virtual *J* and *D* coefficients as functions of (i) the magnitude of the frequency deviation, (ii) RoCoF; (iii) SoC, and (iv) system operating conditions. The main objective is to maximize transient-stability support while preserving the BESS energy reserves.

### 4.1. State-Space Prediction Model

The MPC requires a discrete-time dynamic model capable of predict the future states of the proposed hybrid microgrid [38]. The aggregated state vector is defined as:

(36) $$x\left(k\right)=\left[\begin{array}{c} \Delta f(k)\\ RoCoF(k)\\ SoC(k)\\ P_{\mathrm{BESS}}(k) \end{array}\right]$$

where Δ*f* denotes the frequency deviation. The aggregated state vector is defined as:

(37) $$u\left(k\right)=\left[\begin{array}{c} P_{\mathrm{BESS}}(k)\\ J_{n}(k)\\ D_{n}(k) \end{array}\right]$$

where $J_{n}\left(k\right)$ and $D_{n}\left(k\right)$ are normalized inertia factor and normalized damping factor. The discrete-time prediction model becomes:

(38) $$x\left(k+1\right)\;=\;\mathrm{Ax}\left(k\right)+B_{P}P_{\mathrm{BESS}}\left(k\right)+B_{J}J_{n}\left(k\right)+\;B_{D}D_{n}(k)\;+\;E\;d(k)$$

where:

*A* is the state transition matrix.
*B* is the control input matrix.
*E* represents disturbance influence.
*d*(*k*) contains renewable generation variations and load disturbances.

The output equation is:

(39) $$y\left(k\right)\;=\;\mathrm{Cx}(k)$$

where:

(40) $$y\left(k\right)=\left[\begin{array}{c} \Delta f(k)\\ RoCoF(k) \end{array}\right]$$

represent the main transient-stability indicators.

#### Linearization and Discrete-Time Matrix Construction

The predictive model used by the MPC is obtained through a local linearization of the nonlinear electromechanical dynamics and the BESS around the current operating point. This approach preserves the nonlinear behavior of the detailed plant model in MATLAB–Simulink^®^ while providing the MPC with a computationally efficient local prediction model. At each sampling instant of the control, the measured state vector is used to define the current operating point, and the nonlinear state equations are linearized with respect to the state variables, control, and perturbations.

Let the nonlinear aggregated model be represented by:

(41) $$\dot{x}\left(t\right)=f\left(x\left(t\right),\;u\left(t\right),\;d\left(t\right)\right)\;y\left(t\right)=h\left(x\left(t\right)\right),$$

where:

(42) $$x={\left[\Delta f\;\mathrm{RoCoF}\;\mathrm{SoC}\;P_{\mathrm{BESS}}\right]}^{T}$$

and:

(43) $$u={\left[\begin{array}{ccc} P_{\mathrm{BESS}} & J_{n} & D_{n} \end{array}\right]}^{T}$$

At the *k*-th sampling instant, the operating point is defined as:

$$\begin{array}{ccc} {\bar{x}}_{k}=x\left(k\right), & {\bar{u}}_{k}=u\left(k\right), & {\bar{d}}_{k}=d\left(k\right). \end{array}$$

A first-order Taylor expansion of the nonlinear model around this operating point yields:

(44) $$\Delta \dot{x}\left(t\right)=A_{c}\left(k\right)\Delta x\left(t\right)+B_{P,c}\left(k\right)\Delta P_{\mathrm{BESS}}\left(t\right)+B_{J,c}\left(k\right)\Delta J_{n}\left(t\right)+B_{D,c}\left(k\right)\Delta D_{n}\left(t\right)+B_{d}\left(k\right)\Delta d\left(t\right)$$

where the local Jacobian matrices are obtained from:

(45) $$A_{c}\left(k\right)={\left.\frac{\partial f}{\partial x}\right|}_{{\bar{x}}_{k},{\bar{u}}_{k},{\bar{d}}_{k}},$$

(46) $$B_{P,c}\left(k\right)={\left.\frac{\partial f}{\partial_{BESS}}\right|}_{{\bar{x}}_{k},{\bar{u}}_{k},{\bar{d}}_{k}},$$

(47) $$B_{J,c}\left(k\right)={\left.\frac{\partial f}{{\partial J}_{n}}\right|}_{{\bar{x}}_{k},{\bar{u}}_{k},{\bar{d}}_{k}},$$

(48) $$B_{D,c}\left(k\right)={\left.\frac{\partial f}{{\partial D}_{n}}\right|}_{{\bar{x}}_{k},{\bar{u}}_{k},{\bar{d}}_{k}},$$

and:

(49) $$E_{c}\left(k\right)={\left.\frac{\partial f}{\partial d}\right|}_{{\bar{x}}_{k},{\bar{u}}_{k},{\bar{d}}_{k}},$$

The local continuous-time model is subsequently discretized using the MPC sampling period $T_{s}$ = 10 ms. In this way, the discrete-time state transition matrix and the input matrices are obtained from the local continuous-time representation. In compact form:

(50) $$x\left(k+1\right)=A\left(k\right)x\left(k\right)+B_{P}\left(k\right)P_{\mathrm{BESS}}\left(k\right)+B_{J}\left(k\right)J_{n}\left(k\right)+B_{D}\left(k\right)D_{n}\left(k\right)+B_{d}\left(k\right)d\left(k\right)$$

where $B_{d}$ denotes the disturbance-input matrix. Consequently, the matrices depend on the operating point rather than being fixed globally. This iterative linearization process allows the prediction model to adapt to changes in frequency deviation, BESS SoC, BESS power, renewable generation, and the operational conditions of the hybrid microgrid. The detailed nonlinear model in MATLAB–Simulink^®^ continues to serve as the plant model for validation. The locally linearized model is used exclusively by the MPC supervisor to predict the short-term evolution of relevant stability states.

The output matrix is defined based on the transient-stability variables used in the optimization:

(51) $$C=\left[\begin{array}{cccc} 1 & 0 & 0 & 0\\ 0 & 1 & 0 & 0 \end{array}\right]$$

yielding:

(52) $$y\left(k\right)=\left[\begin{array}{c} \Delta f\;(t)\\ \mathrm{RoCoF}\;(t) \end{array}\right]$$

This formulation clarifies the relationship between the nonlinear model of the plant, its local linear prediction model, and the discrete-time MPC formulation.

### 4.2. MPC Prediction Horizon

The proposed MPC minimizes a transient-stability-oriented objective function that penalizes frequency deviation, RoCoF, BESS energy depletion, and aggressive control actions. At each sampling instant, the controller predicts the future behavior of the system over a finite horizon, where prediction steps are considered. The control horizon is selected to provide sufficient flexibility while maintaining computational efficiency. The optimization is executed at every sampling interval $T_{s}\;=\;10\;\mathrm{ms}$, which is compatible with practical implementations of BESS converters.

### 4.3. Multi-Objective Optimization Function

One of the main contributions of this work is the formulation of a transient-stability-oriented objective function [39]. The proposed MPC minimizes:

(53) $$J_{\mathrm{MPC}}\;=\;J_{f}+J_{r}+J_{e}+J_{u}$$

where:

$J_{f}$ penalizes frequency deviations.
$J_{r}$ penalizes RoCoF.
$J_{e}$ penalizes excessive battery energy usage.
$J_{u}$ penalizes aggressive control actions.

The complete objective function is:

(54) $$J_{\mathrm{MPC}}\;=\sum_{i=1}^{N_{p}}\left[\omega_{f}\;\Delta f_{k+i}^{2}+\omega_{r}{\mathrm{RoCoF}}_{k+i}^{2}\;+\;\omega_{E}\;\Delta \;E_{k+i}^{2}\right]+\sum_{i\;=\;0}^{N_{c}-1}\omega_{u}{\left\|{\Delta u}_{k+i}\right\|}^{2}$$

where:

$\omega_{f}$ is the frequency weighting factor.
$\omega_{r}$ is the RoCoF weighting factor.
$\omega_{s}$ is the energy-preservation weighting factor.
$\omega_{u}$ penalizes control effort.

This formulation explicitly incorporates transient stability and BESS sustainability within a unified optimization framework.

### 4.4. Dynamic Optimization of Virtual Inertia and Damping

Unlike conventional adaptive-speed SGs, the proposed MPC directly determines the control input $J_{n}$. The optimization problem seeks [40]:

(55) $$J_{n}^{*}\;=\arg\min J_{\mathrm{MPC}}$$

subject to:

(56) $$J_{n\text{\_}\min}\left(\mathrm{SoC}\right)\leq J_{n}\left(k\right)\leq J_{n\text{\_}\max}\left(\mathrm{SoC}\right)$$

This mechanism allows inertia to be increased automatically during severe disturbances and reduced during normal operation. Consequently, synthetic $J_{n}$ becomes adaptive and optimal.

To avoid ambiguity between the optimization variable and the physical parameter VSG, the proposed MPC operates on a normalized inertia factor $J_{n}$, while the AEA-VSG block uses the corresponding physical virtual inertia $J_{v}$, indicated in $\mathrm{kg}\cdot m^{2}$. The ratio between the two quantities is defined as:

(57) $$J_{v}\left(k\right)\;=\;J_{\mathrm{scale}}*{\;J}_{n}(k)$$

where $J_{\mathrm{scale}}$ = 62.18 $\mathrm{kg}\cdot m^{2}$, Then:

(58) $$J_{v}\left(k\right)\;=\;62.18\;J_{n}(k)$$

and:

(59) $$J_{n}(k)\;=\frac{J_{v}\left(k\right)}{62.18}$$

The implemented normalization assigns the maximum normalized inertia factor of $J_{n}$ = 0.78 to the maximum observed physical virtual inertia of 48.5 kg·m^2^, resulting in an effective scale factor of approximately 62.18 kg·m^2^.

Similarly, the damping coefficient is optimized according to:

(60) $$D_{n\;}^{*}\;=\arg\min J_{\mathrm{MPC}}$$

Subject to:

(61) $$D_{n\text{\_}\min}\leq D_{n}\left(k\right)\leq D_{n\text{\_}\max}$$

Therefore, it is possible to establish:

(62) $$D_{\mathrm{scale}}\;=\;\frac{D_{v}}{D_{n}\left(t_{D}^{*}\right)}=\frac{38.2}{D_{n}\left(t_{D}^{*}\right)}$$

In this way, this can be obtained:

(63) $$D_{v}\left(k\right)\;=\;D_{\mathrm{scale}}{\;D}_{n}(t_{D}^{*})$$

The damping term $D_{v}$ suppresses post-fault oscillations while avoiding excessive control actions.

With this, we can formally define:

(64) $$z\left(k\right)=\left[\begin{array}{c} J_{n}(k)\\ D_{n}(k) \end{array}\right]$$

So their physical transformation will be:

(65) $$z\left(k\right)=\left[\begin{array}{c} J_{n}(k)\\ D_{n}(k) \end{array}\right]=\left[\begin{array}{cc} J_{\mathrm{scale}} & 0\\ 0 & D_{\mathrm{scale}} \end{array}\right]z\left(k\right)$$

or equivalently:

$$J_{\mathrm{scale}}\;=\;62.18\;J_{n}(k)$$

$$D_{\mathrm{scale}}\;=\;130.3\;D_{n}(k)$$

Separately, the adaptive controller uses:

(66) $$G_{J}\;\in \;\left[1,\;10\right]$$

(67) $$G_{D}\;\in \;\left[0.1,\;5\right]$$

$G_{J}$ and $G_{D}$ are controller parametrization gains and not MPC decision variables or dimensional VSG parameters.

### 4.5. Energy-Constrained Optimization

BESS sustainability is incorporated through explicit SoC constraints. The MPC optimization is subject to:

(68) $${\mathrm{SoC}}_{\min}\leq \mathrm{SoC}(k)\leq {\mathrm{SoC}}_{\max}$$

where:

(69) 20%≤SoC≤90%

In addition:

(70) $$-P_{\mathrm{BESS}\text{\_}\min}\leq P_{\mathrm{BESS}}(k)\leq P_{\mathrm{BESS}\text{\_}\max}$$

with $P_{\mathrm{BESS}\text{\_}\max}\;=\;1.0\;\mathrm{MW}.$ The BESS ramp-rate constraint defined as:

(71) $$\left|\frac{dP_{\mathrm{BESS}}}{\mathrm{dt}}\right|\leq {\mathrm{RR}}_{\max}$$

These limitations ensure physically feasible operation.

To ensure safe, reliable, and physically feasible BESS operation, a set of operating constraints is incorporated into both the energy-aware adaptive VSG framework and the MPC optimization process. These constraints represent the electrical, energy, and dynamic limits imposed by the BESS cells, the power converter, and the energy-management requirements. Specifically, limits are imposed on the SoC, terminal voltage, current, power exchange, stored energy, ramp rate, virtual inertia, and virtual damping to avoid excessive stress on the BESS, maintain converter integrity, and ensure long-term operational sustainability. The operating limits adopted in this study are summarized in Table 19.

### 4.6. MPC-Based Synthetic Inertia Allocation

The synthetic-*J* power command $\left(P_{\mathrm{SI}}\right)$ is calculated using the MPC-based virtual coefficient $J_{\mathrm{optimal}}$.

(72) $$P_{\mathrm{SI}}=2J_{n}^{*}\omega \frac{d\omega }{\mathrm{dt}}$$

The effective support power is converted into:

(73) $$P_{\mathrm{SI}}^{\mathrm{eff}}\;=\;W_{\mathrm{SoC}}\;P_{\mathrm{SI}}$$

where:

(74) $$W_{\mathrm{SoC}}\;=\;\frac{\mathrm{SoC}-{\mathrm{SoC}}_{\min}}{{\mathrm{SoC}}_{\max}-{\mathrm{SoC}}_{\min}}$$

This formulation enables adaptive allocation of synthetic *J* according to disturbance severity and the available BESS energy [41].

The proposed MPC-based synthetic-inertia allocation framework was implemented using a quadratic-programming formulation solved at each control interval through the MATLAB MPC Toolbox. The optimization problem was formulated as a constrained finite-horizon quadratic-cost minimization and was solved using an active-set quadratic-programming solver because of its numerical robustness and suitability for small- and medium-scale power-system applications.

After extensive preliminary tuning studies, a prediction horizon of $N_{p}\;=\;20$ steps and a control horizon of $N_{c}\;=\;10$ steps were selected, providing an appropriate balance between prediction accuracy and computational burden. The MPC optimization was executed with a sampling period of $T_{s}$ [ms], fast enough to capture frequency transients and synthetic-inertia dynamics while remaining compatible with practical digital-controller implementations.

The computational performance of the proposed controller was evaluated under all simulated operating conditions and disturbance scenarios. The average optimization time was approximately 2.8 ms per control cycle, while the maximum computation time observed during severe transient events remained below 6.5 ms. Because both values are significantly shorter than the adopted sampling interval, the proposed controller satisfies real-time implementation requirements and ensures closed-loop execution without computational overrun.

To ensure optimization feasibility under all operating conditions, soft constraints were introduced for frequency deviation and synthetic-inertia allocation variables through slack-variable penalization in the objective function (see Table 20). This strategy avoids infeasible solutions during severe disturbances while maintaining acceptable control performance. In addition, a constraint-smoothing mechanism based on gradual activation of power and inertia limits was incorporated to avoid abrupt control actions, improve numerical conditioning, and reduce chattering phenomena in the BESS power reference. Consequently, the proposed MPC framework maintains stable and feasible operation even under large disturbances, high-renewable-energy penetration levels, and low BESS SoC conditions.

**Figure 8:**
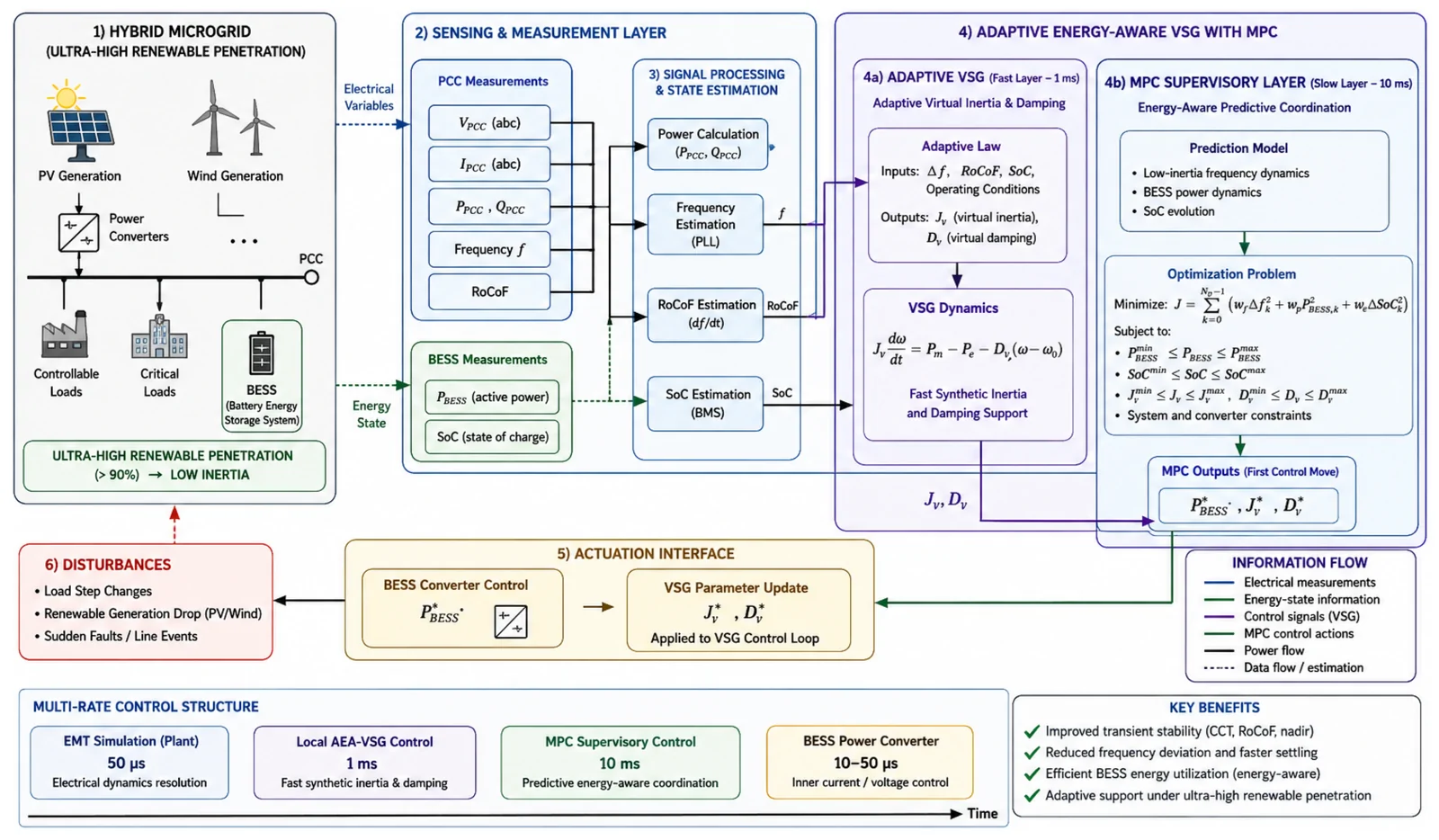
Proposed methodology.

With the methodology expressed in Section 3 and Section 4, the proposed MPC-driven adaptive energy-aware VSG is expected to provide the following benefits under ultrahigh-renewable-energy penetration conditions, as shown in Figure 8 and Table 21.

Compared with conventional fixed-parameter VSG approaches, the proposed framework enables simultaneous optimization of transient stability and energy-management objectives, making it particularly suitable for future renewable-dominated microgrids operating near 100% renewable-energy penetration.

## 5. Simulation Framework and Performance Evaluation Methodology

### 5.1. Simulation Environment and Computational Framework

The proposed MPC–VSG–BESS framework was evaluated through electromagnetic transient (EMT) simulations implemented in MATLAB–Simulink^®^ using the Simscape Electrical™ environment. The full simulation horizon was set to 60 s, with the main disturbances starting at t = 20 s. The 20 s pre-disturbance interval was used to establish the steady-state operating condition, while the remaining 40 s provided enough time after the disturbance to assess the transient evolution, recovery behavior, and energy response of the BESS.

A fixed-step discrete numerical solver was used with an electrical integration interval of 50 µs. This integration interval differs from the sampling periods of the control layers. This difference is crucial because the EMT grid needs to resolve the rapid dynamics of the electrical components and the converter, while supervisory controllers operate at lower sampling frequencies (see Table 22).

Therefore, the proposed control architecture follows a multi-rate sampling implementation. The local AEA-VSG controller operates with a 1 ms sampling period, while the finite-horizon supervisory MPC operates at 10 ms. The AEA-VSG layer provides the fast dynamic response needed for frequency regulation and RoCoF, while the MPC layer performs predictive coordination of the BESS power support and energy-dependent adaptation of the virtual inertia and damping parameters.

The system’s nominal frequency is 60 Hz and the voltage at the PCC is 13.8 kV. A base apparent power of 100 MVA is adopted for the system consistently throughout the per-unit formulation. The MPC uses a prediction horizon of $N_{p}$ = 20 samples and a control horizon of $N_{c}$ = 10 samples. With an MPC sampling period of 10 ms, these values correspond to a prediction window of 0.2 s and a control action horizon of 0.1 s.

The BESS is represented with a nominal power of 1 MW and an energy capacity of 2 MWh. The nominal power is distinguished from its transient power capacity. During severe contingencies, the BESS converter is allowed to operate temporarily above its continuous nominal power capacity, in accordance with the transient overload capacity implemented in the simulation model. Therefore, the power values recorded during the most severe disturbances should not be interpreted as the BESS’s continuous nominal power. The corresponding transient overload condition and its duration are explicitly considered when evaluating the feasibility of the BESS.

The same electrical grid model and the same numerical configuration were maintained for the different renewable-energy penetration scenarios. Consequently, the variations in the transient response are attributed to the composition of renewable generation, the severity of the disturbance, and the operation of the control, rather than to changes in the numerical integration parameters.

To isolate the effect of renewable penetration and the displacement of synchronous inertia, seven operational scenarios were established with renewable-energy penetration levels of 10%, 20%, 30%, 50%, 70%, 90%, and 100%. For each scenario, the total renewable contribution was defined based on the combined active power of PV units, DFIG, and PMSG, while diesel and CCGT units were progressively displaced as renewable penetration increased. The grid topology, the voltage level at the PCC, the nominal load, and the controller architecture remained unchanged across all scenarios. Consequently, the main variable between scenarios is the relative contribution of interconnected renewable generation through converters versus synchronous generation.

The BESS was treated independently from the renewable-penetration metric. Its nominal capacity remained at 1 MW/2 MWh in all scenarios, while its instantaneous active-power response was determined using the proposed control strategy based on MPC, subject to the corresponding power, energy, current, voltage, ramp rate, and SoC constraints. This separation prevents the storage device from being interpreted as a renewable generation source and allows for the assessment of its contribution to transient stability independently of the renewable-penetration definition as indicated in the Table 23.

### 5.2. Severe Contingency Definitions

The proposed hybrid microgrid was subjected to two categories of severe contingency: (i) a three-phase ground short-circuit fault with zero impedance on the 4.16 kV primary distribution bus, and (ii) the sudden loss of the main conventional generation unit. A three-phase fault is applied to the specified internal distribution bus, while the main electrical response variables used for system-level evaluation are monitored at the 13.8 kV PCC. Therefore, the voltage level at the fault point and the measurement voltage at the PCC should not be interpreted as the same electrical bus.

These contingencies were selected to evaluate the ability of the proposed MPC–VSG–BESS scheme to maintain transient stability in the face of severe disturbances and increasing penetration of renewable energies. Since the total simulation duration is 60 s, the model provides a 40 s window after the disturbance to assess the transient dynamics and subsequent recovery. It is important to mention that the CCT is defined as the maximum duration of the applied fault for which the post-fault system remains within prescribed transient-stability limits. Consequently, the CCT is expressed in milliseconds, as the fault duration sweep is conducted within the sub-second-scale transient interval. The CCT should be distinguished from the total post-fault recovery time, which can extend over several seconds.

Extreme Three-Phase Symmetric Short-Circuit Fault

This scenario evaluates transient voltage stability and the electromechanical coupling of the virtual rotor. At t = 20 s, a three-phase ground short-circuit fault with zero impedance is applied to the primary distribution bus at 4.16 kV. The fault remains active for a variable duration $\left(T_{\mathrm{fault}}\right)$ ranging from 50 ms to 250 ms to identify the absolute CCT threshold before the microgrid undergoes voltage collapse due to loss of synchronism. During the fault, the inner current loops of the BESS inverter must limit the output to a maximum safe thermal threshold of 1.2 pu, testing the MPC’s ability to handle strict output-saturation limits.

Abrupt Loss of Main Synchronous Generation Asset (Generation Trip)

This scenario evaluates active-power deficit management and frequency-regulation speed under different penetration levels. At 20 s, a sudden mechanical failure triggers the emergency isolation breaker of the 2 MW CCPP, instantly removing 20% of the online capacity of the proposed hybrid microgrid. This event produces a massive and immediate power mismatch between the remaining generation and the 10.5 MW ZIP load. The test evaluates how rapidly the adaptive framework increases J and D to arrest the fast frequency drop before localized underfrequency load-shedding (UFLS) relays, calibrated at 57.5 Hz, are activated.

#### 5.2.1. Case Study 1: Extreme Three-Phase Symmetric Short-Circuit Fault—Base-Case Validation

**Figure 9:**
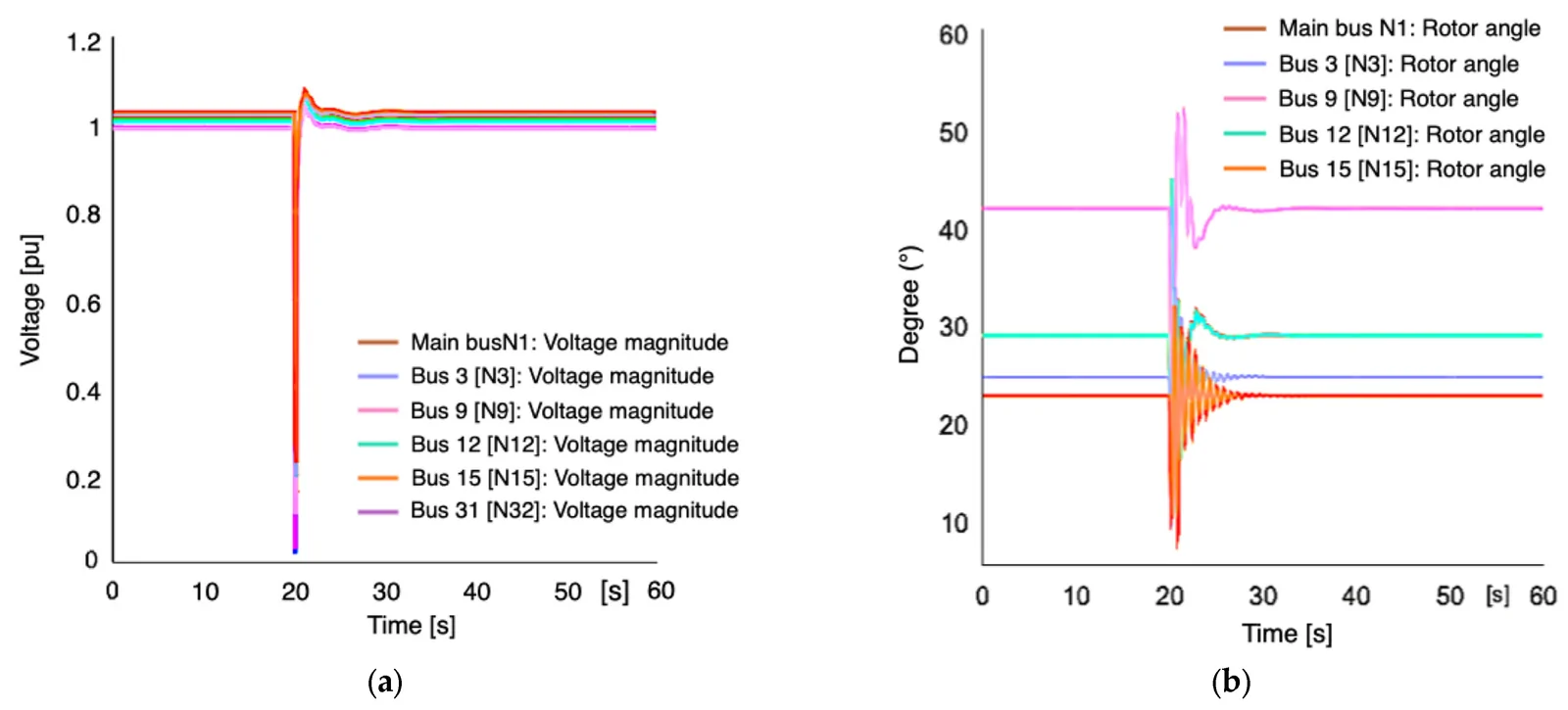
Variation in (**a**) voltage at buses of the microgrid and (**b**) rotor angles of the SGs.

**Figure 10:**
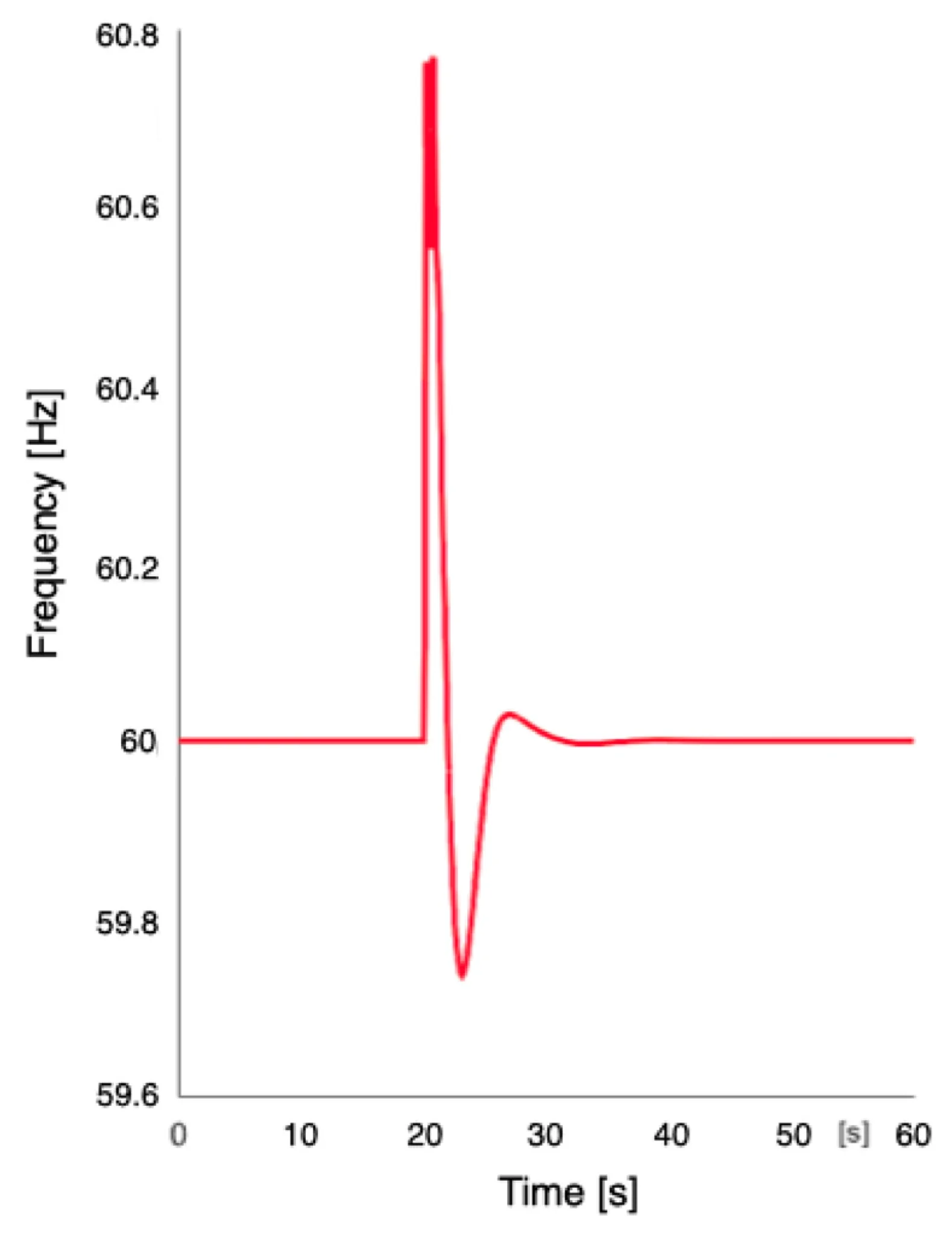
Frequency variation.

**Figure 11:**
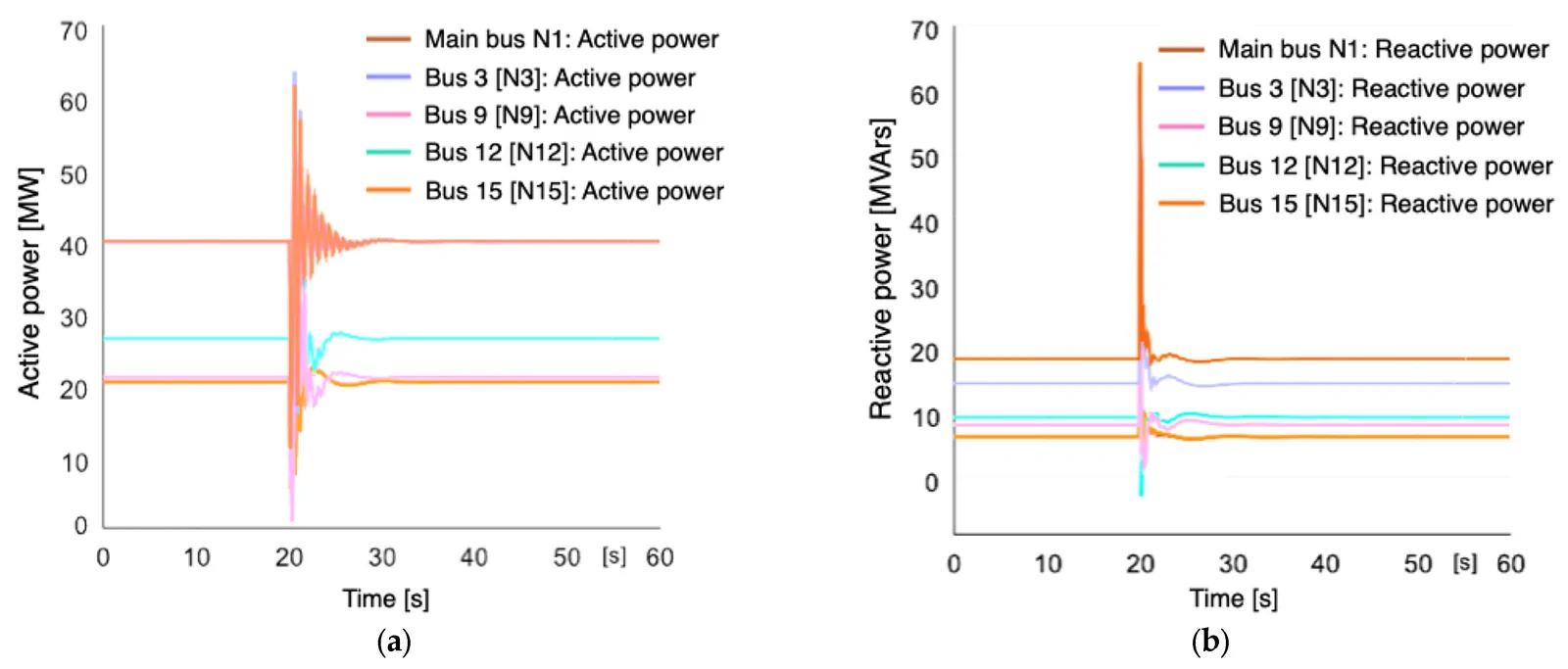
Variation in (**a**) active power and (**b**) reactive power of the SGs.

A three-phase short circuit occurred at the midpoint of node N1, with a duration of 150 ms and a short-circuit impedance of 0.1 Ω. The system response during this transient is illustrated in Figure 9, Figure 10 and Figure 11, which show that despite the initial oscillation in voltage, frequency, and rotor angle of the SGs at the beginning of the transient, the microgrid remained stable after the incident. The SGs demonstrated sufficient capability to restore grid stability after the disturbance. The maximum and minimum recorded frequencies were 60.77 Hz and 59.74 Hz, respectively. Over a 500 ms period, RoCoF reached 1.23 Hz/s. In this scenario, the CCT was determined to be 270 ms. This occurs because the frequency deviation exceeds the acceptable range of 54–64 Hz, which activates the protection relays and opens the circuit breakers at the terminals of the SGs, causing a blackout.

#### 5.2.2. Case Study 2: Extreme Three-Phase Symmetric Short-Circuit Fault—Renewable-Energy Penetration = 10%

In this scenario, the PV systems are assigned a total rated power of 0.3 MW, resulting in a rated power of 100 kW for each PV system. In addition, two WTs with a rated power of 1.2 MW each are activated and connected at node N16. The sum of renewable penetration from the PV systems and the WTs is equal to 10%.

**Figure 12:**
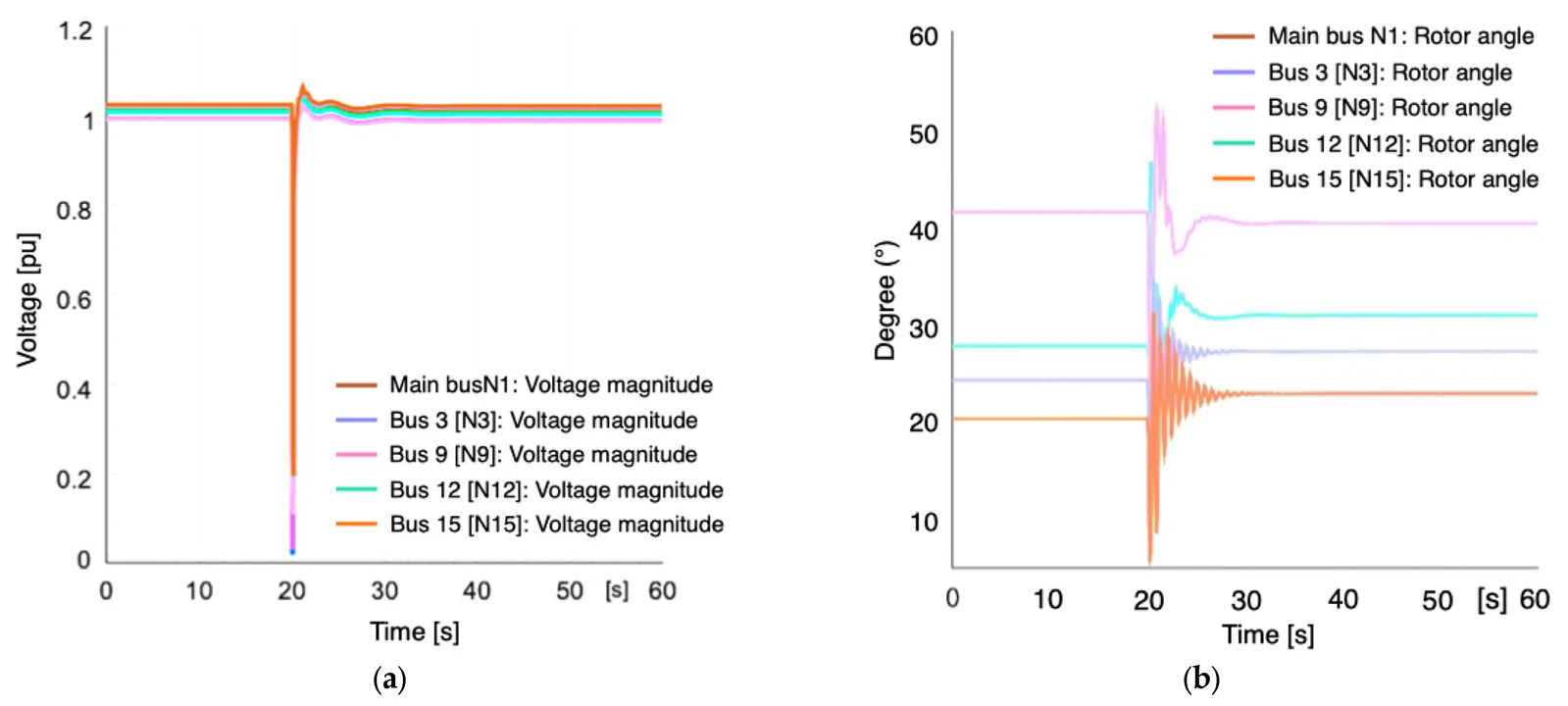
Variation in (**a**) voltage at buses of the microgrid and (**b**) rotor angles of the SGs.

**Figure 13:**
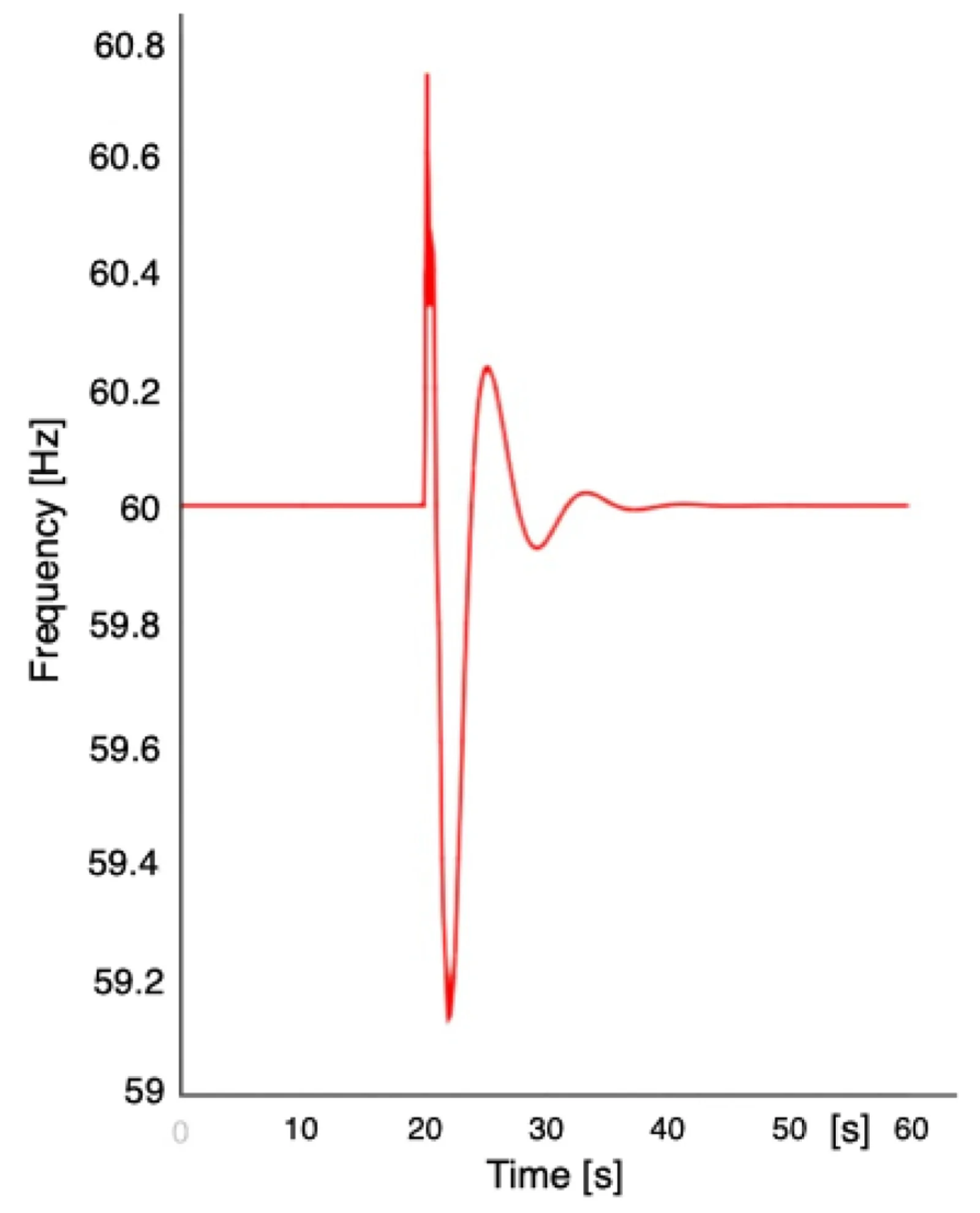
Frequency variation.

**Figure 14:**
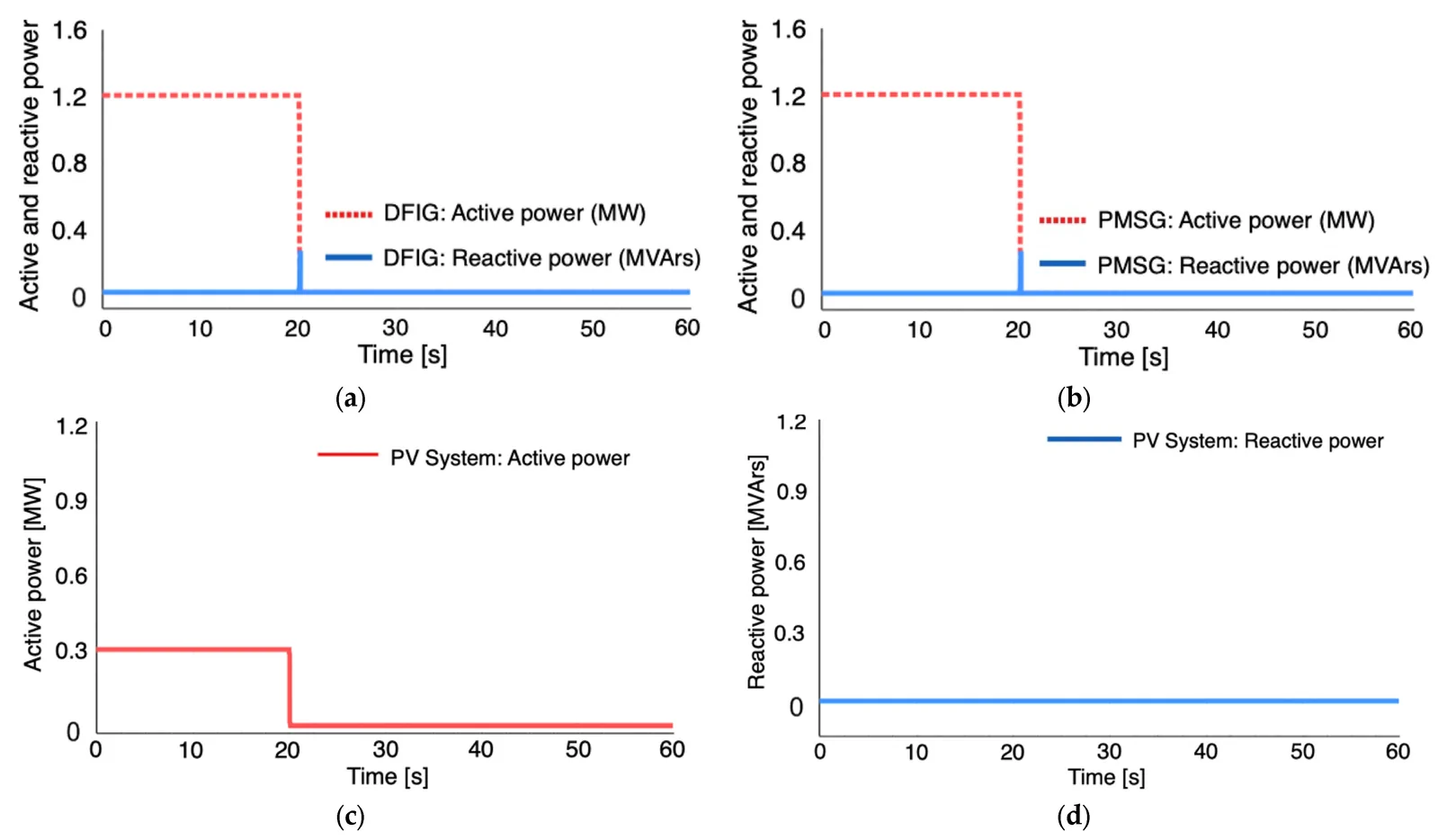
Variation in (**a**) DFIG output power, (**b**) PMSG output power, (**c**) active output power PV system, and (**d**) reactive output power PV system.

**Figure 15:**
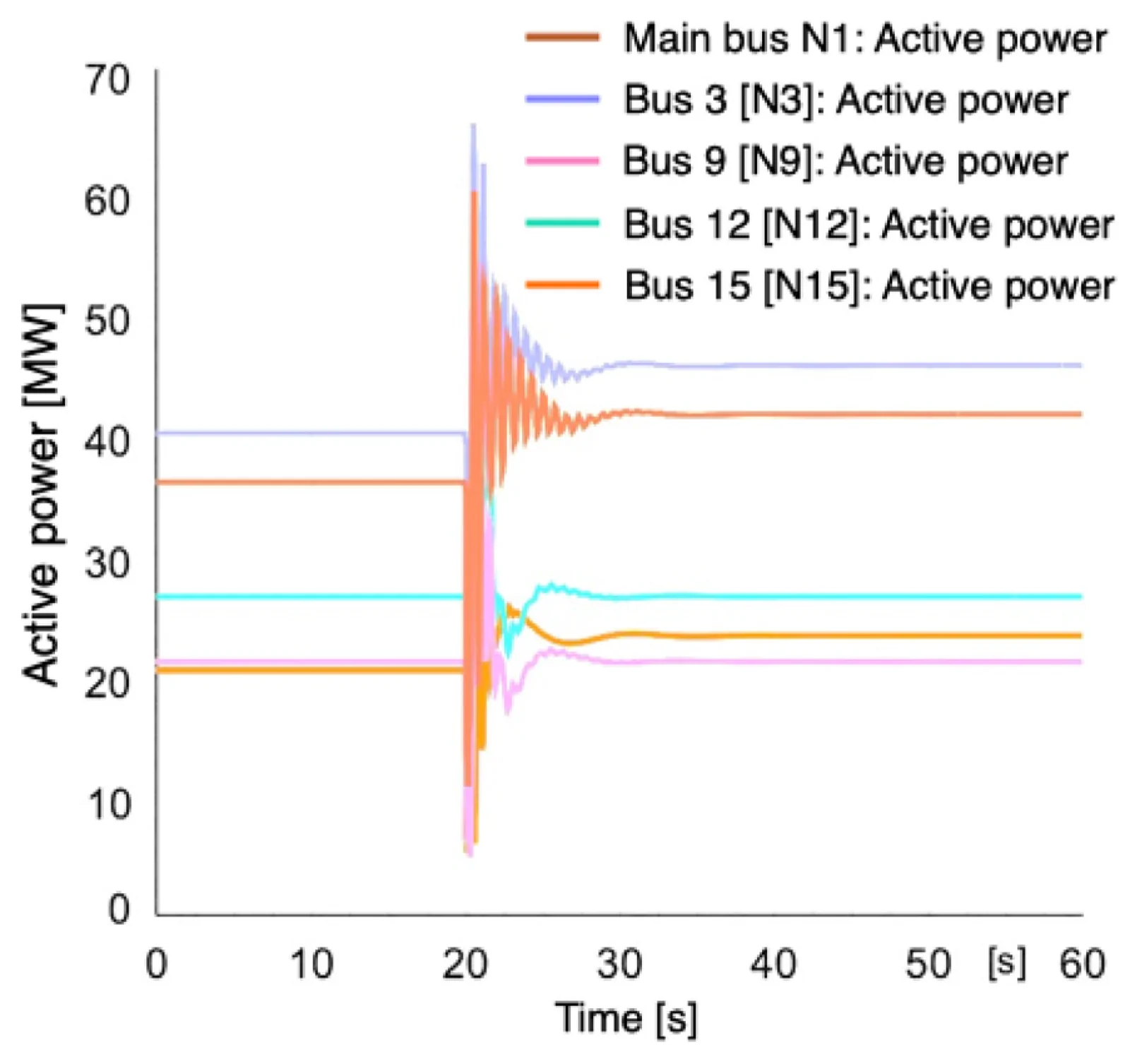
Variation in active power of the SGs.

Figure 12, Figure 13, Figure 14 and Figure 15 show the simulation results for this scenario. In Figure 12, it is observed that the electric hybrid microgrid remains stable when RES penetration reaches 10%. However, several key points should be noted.

As observed in Figure 14, the RESs disconnect from the hybrid microgrid after the disturbance because of a voltage drop below the 0.9 p.u. threshold for 100 ms, resulting in zero power injection into the electric hybrid microgrid. Consequently, the governors of the SGs are automatically activated to increase the power injected into the hybrid microgrid, thereby compensating for the lost capacity (Figure 15). Therefore, the power generated by the SGs after the incident is greater than that generated before the incident.

The parameters used to evaluate the transient stability of the hybrid microgrid during the transient process, such as the frequency nadir, decreased to 59.12 Hz (Figure 15) compared with the previously recorded 59.75 Hz (as shown in Figure 12). The CCT decreased from 270 ms in scenario 1 to 180 ms. Likewise, RoCoF increased to 1.38 Hz/s compared with the 1.3 Hz/s observed in case study 1.

#### 5.2.3. Case Study 3: Extreme Three-Phase Symmetric Short-Circuit Fault—Renewable-Energy Penetration = 20%

In this case study, the WTs are activated with a rated power of 2.4 MW, while the active power of the PV systems increases from 0.5 to 1 MW. The parameters used to evaluate the stability of the hybrid microgrid, such as maximum RoCoF, frequency nadir, and CCT, are 1.87 Hz/s, 58.74 Hz, and 170 ms, respectively.

**Figure 16:**
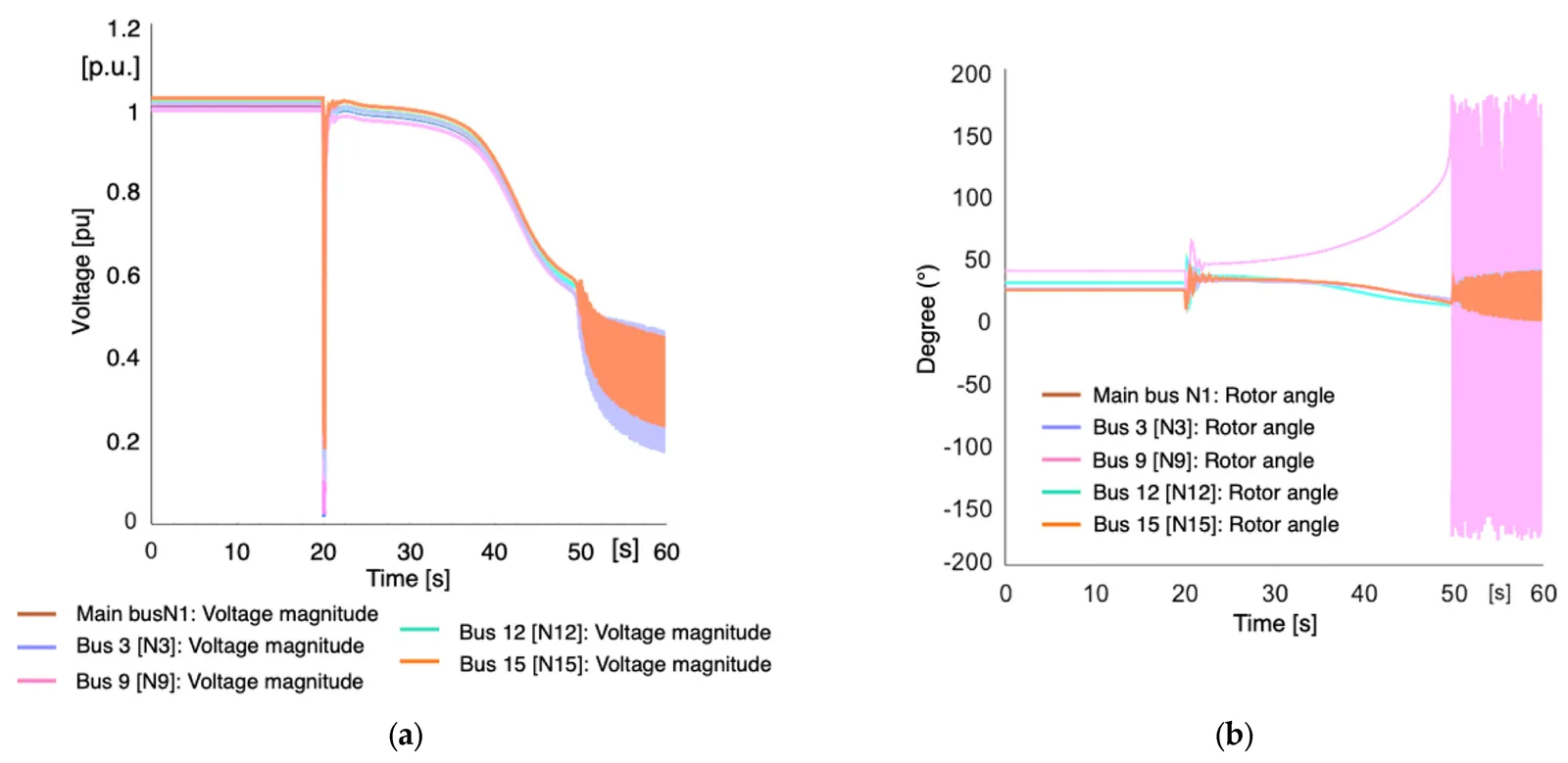
Variation in (**a**) voltage at any node of the electrical microgrid and (**b**) rotor angles of the SGs.

**Figure 17:**
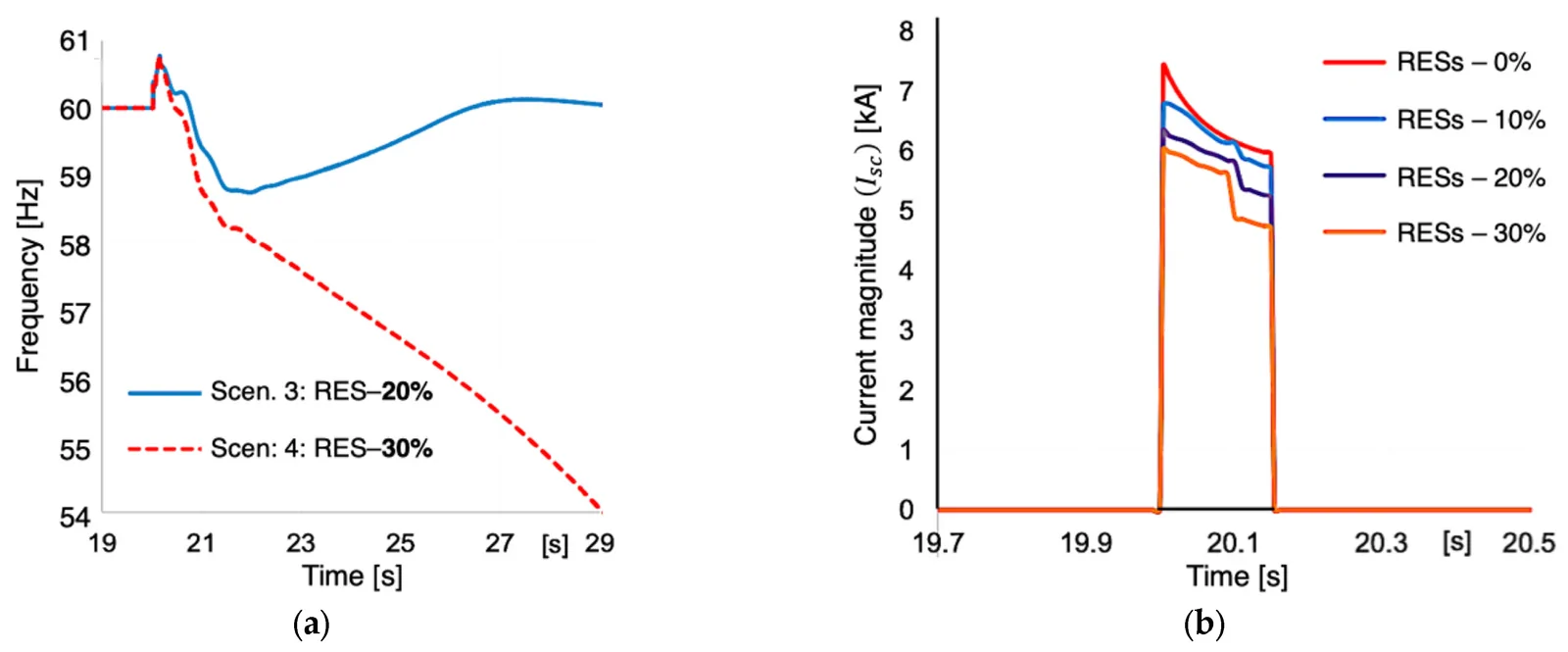
Variation in (**a**) frequency with 20% and 30% renewable penetration and (**b**) short-circuit current magnitude.

**Figure 18:**
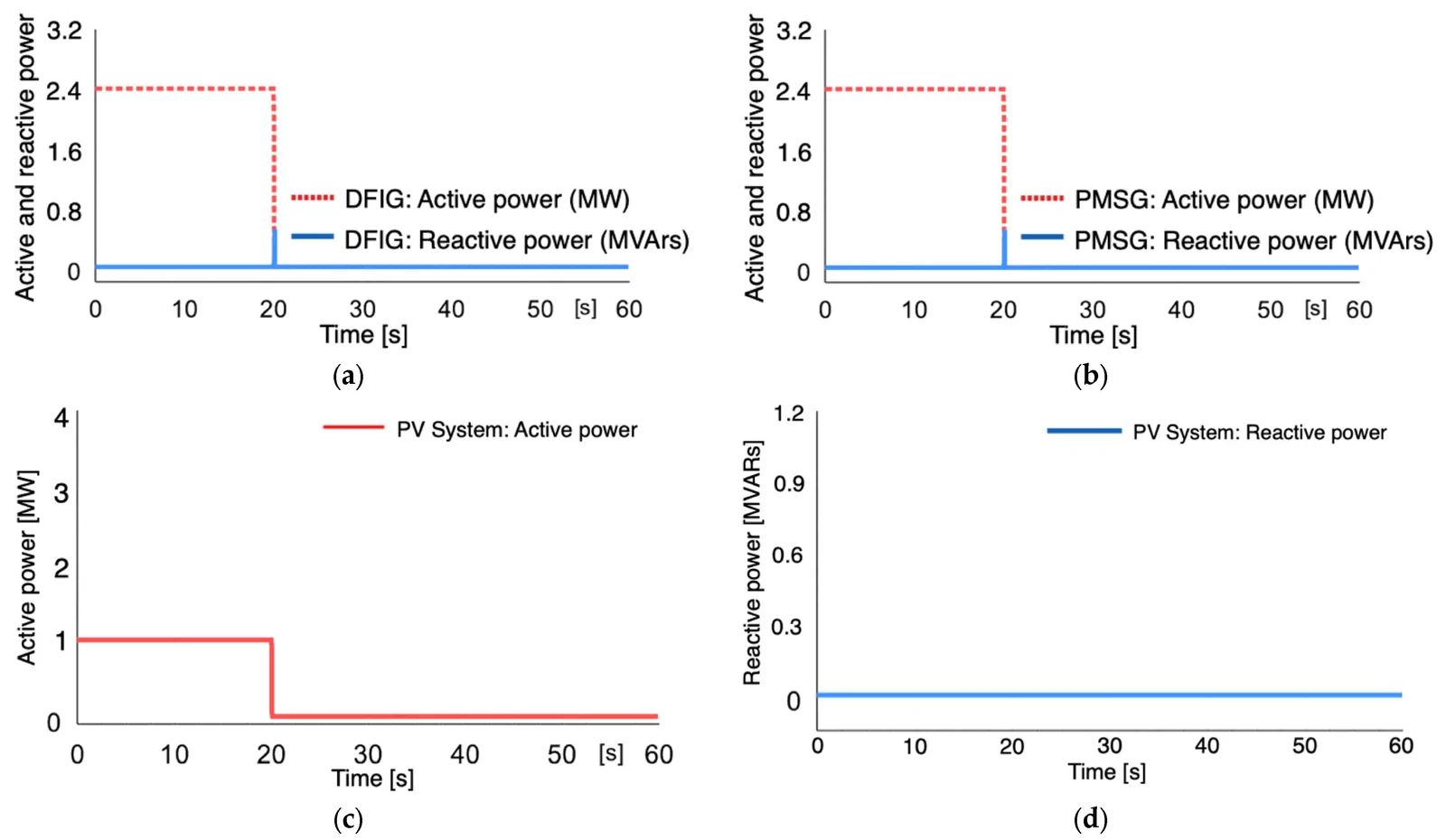
Variation in (**a**) DFIG output power, (**b**) PMSG output power, (**c**) active output power of the PV_1_ system, and (**d**) reactive output power of the PV_2_ system.

**Figure 19:**
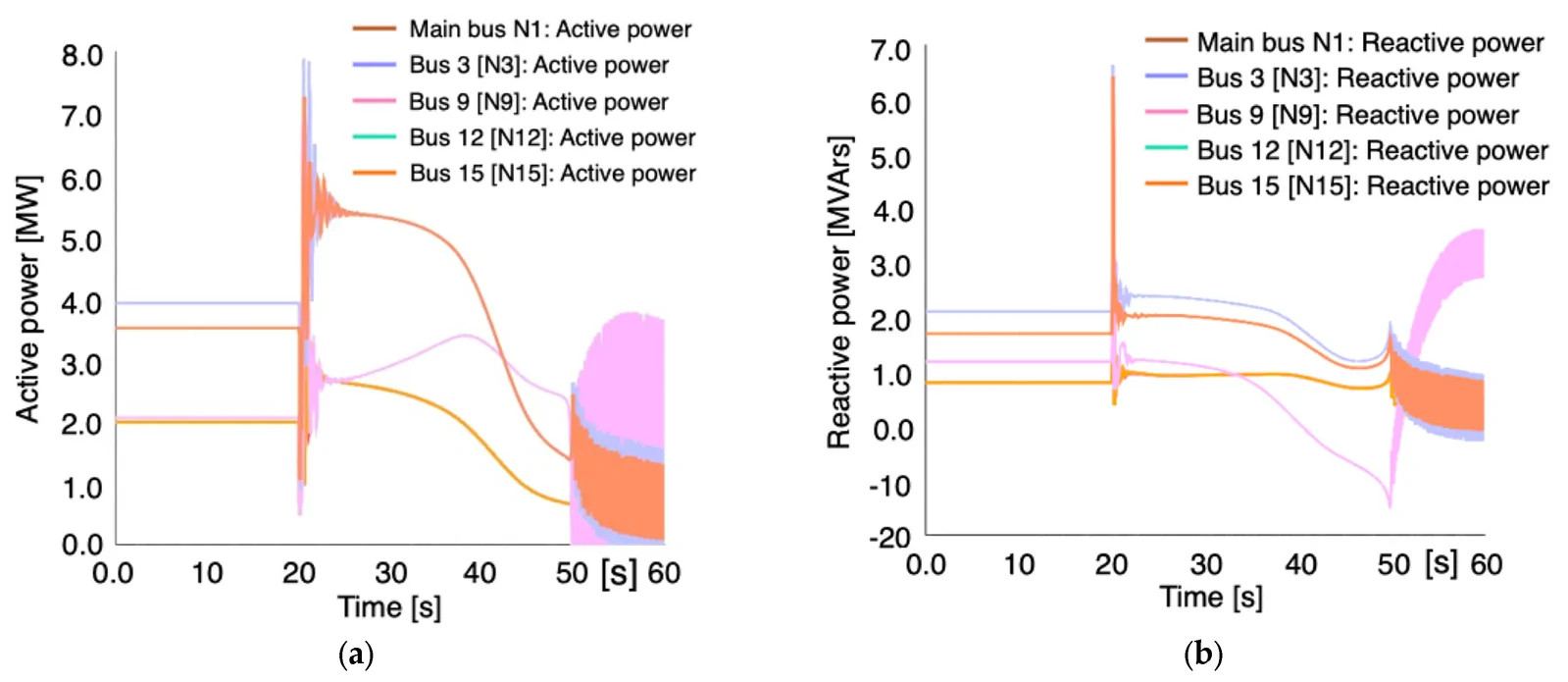
Variation in (**a**) active power and (**b**) reactive power of SGs.

It should be noted that if the duration of the three-phase short circuit at node N1 exceeds the CCT of 170 ms in this case study, the system will lose synchronism. This differs from the previous case, in which activation of the protection relay causes frequency and voltage drops, among other effects, in the electric hybrid microgrid. Figure 16, Figure 17, Figure 18 and Figure 19 show system instability after a three-phase circuit fault with 30% renewable penetration.

While the frequency decreases steadily (Figure 17), the voltage and rotor angle remain stable until 30 s (Figure 16), before dropping sharply and losing synchronism at 50 s (Figure 16a,b). This is explained by the fact that the RESs disconnected from the hybrid microgrid immediately after the incident (Figure 18), producing a generation deficit.

Consequently, the power generated by the SGs increases (Figure 19) to compensate for this deficit. However, this increase is insufficient to offset the power lost from the RESs, resulting in drops in frequency, voltage, and rotor angle.

Therefore, as the number of RESs integrated into the hybrid microgrid increases, the number of SGs decreases, limiting the current injected into the grid and into the short-circuit point. This factor must be considered when setting and coordinating the parameters of protection devices, especially in scenarios with high renewable penetration from PV systems or WTs.

To facilitate observation of the impact of RESs on the overall transient stability of the hybrid microgrid, Table 24 provides a summary of the key parameters used as indicators for this assessment.

Table 24 highlights the following points:

- RESs have a negative impact on the overall transient stability of the hybrid microgrid to which they are connected.
- The higher the renewable-energy penetration in the hybrid microgrid, the larger the oscillations in frequency, voltage, rotor angles, and related variables. In addition, the system may become unstable after a fault if renewable-energy penetration exceeds a certain threshold.

#### 5.2.4. Case Study 4: Main Generator Fault—Renewable Penetration = 50%

**Figure 20:**
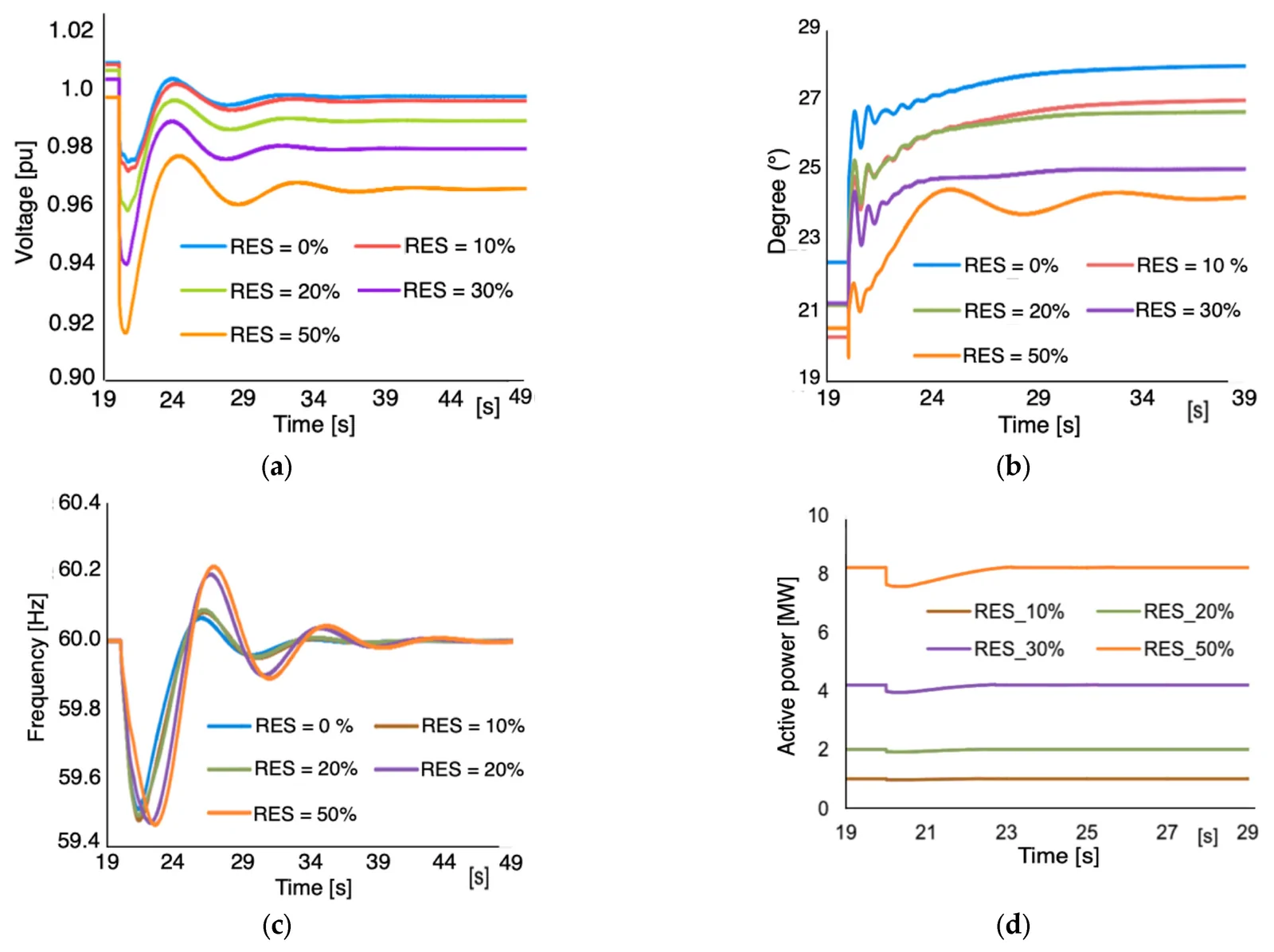
Variation in (**a**) voltage at node N1, (**b**) rotor angles of synchronous generator N15, (**c**) frequency, and (**d**) active output power of PV_1_.

**Figure 21:**
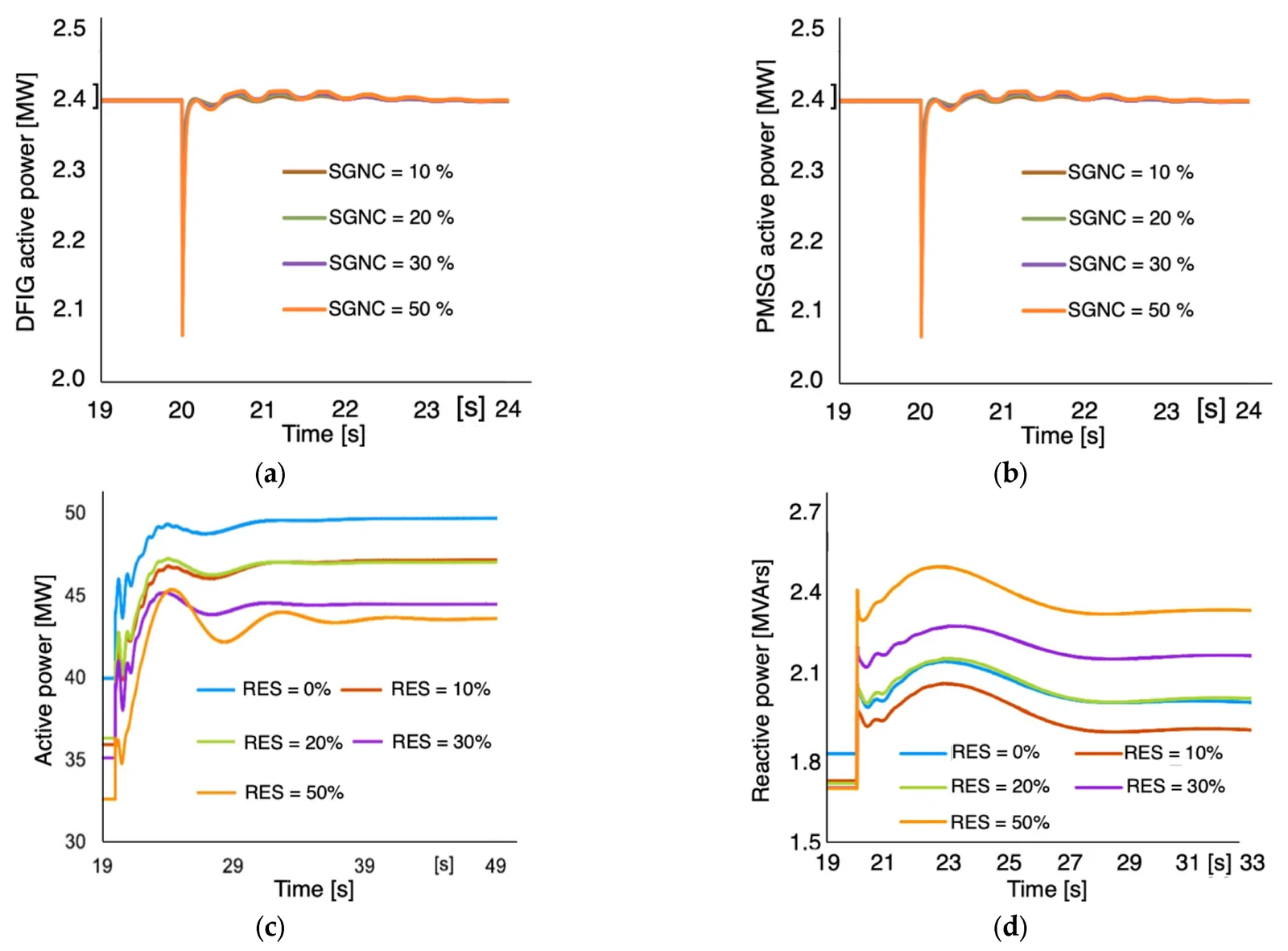
Variation in (**a**) active-power output of the DFIG and PMSG, (**b**) reactive power output of the DFIG and PMSG, (**c**) active-power output of synchronous generator N15, and (**d**) reactive power output of the SG N15.

For this analysis, synchronous generator N15, which acts as the reference generator, is suddenly disconnected after 20 s. The change in the behavior of the hybrid microgrid during this event is shown in Figure 20 and Figure 21.

Several observations can be drawn from these figures:

- Unlike the disturbance caused by a three-phase short circuit, the hybrid microgrid remains stable after the disconnection of N15, even with 50% renewable penetration (Figure 20a–c).
- As renewable penetration increases, the voltage and frequency oscillations, among others, after the N15 contingency also intensify (Figure 20a,c).

During the main-generator outage, the voltage at the PCC between the hybrid microgrid and the main grid fluctuates between 0.9 and 1.1 p.u. (see Figure 20a). This fluctuation does not affect the connection between the RESs and the main grid, because power is continuously injected into the hybrid microgrid from the PV systems and WTs, as illustrated in Figure 20d and Figure 21a,b. This continuous power injection is a factor contributing to system transient stability, ensuring that there is no shortage of power production, unlike what occurs during a short-circuit fault.

After the outage of generator N15, the remaining SGs in the hybrid microgrid immediately increase their power output to compensate for the loss caused by the inactivity of generator N15 (see Figure 21c,d). These generators have sufficient capacity to compensate for the reduction in N15 power production, allowing the system to quickly reach a balance between generation and energy consumption, thereby maintaining stability.

#### 5.2.5. Case Study 5: Main Generator Fault—Renewable Penetration = 70%

**Figure 22:**
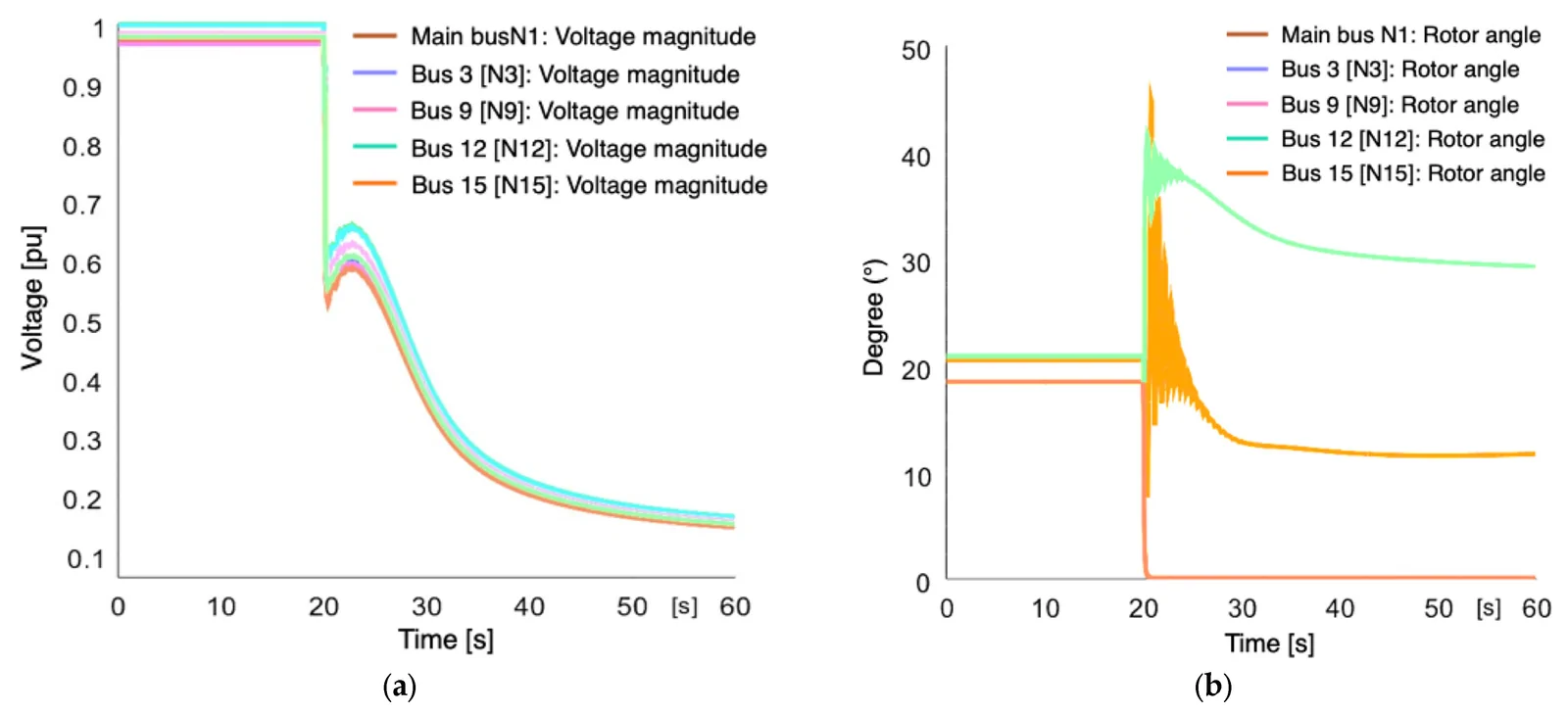
Variation in (**a**) voltage at any node of the microgrid and (**b**) rotor angles of the SGs.

**Figure 23:**
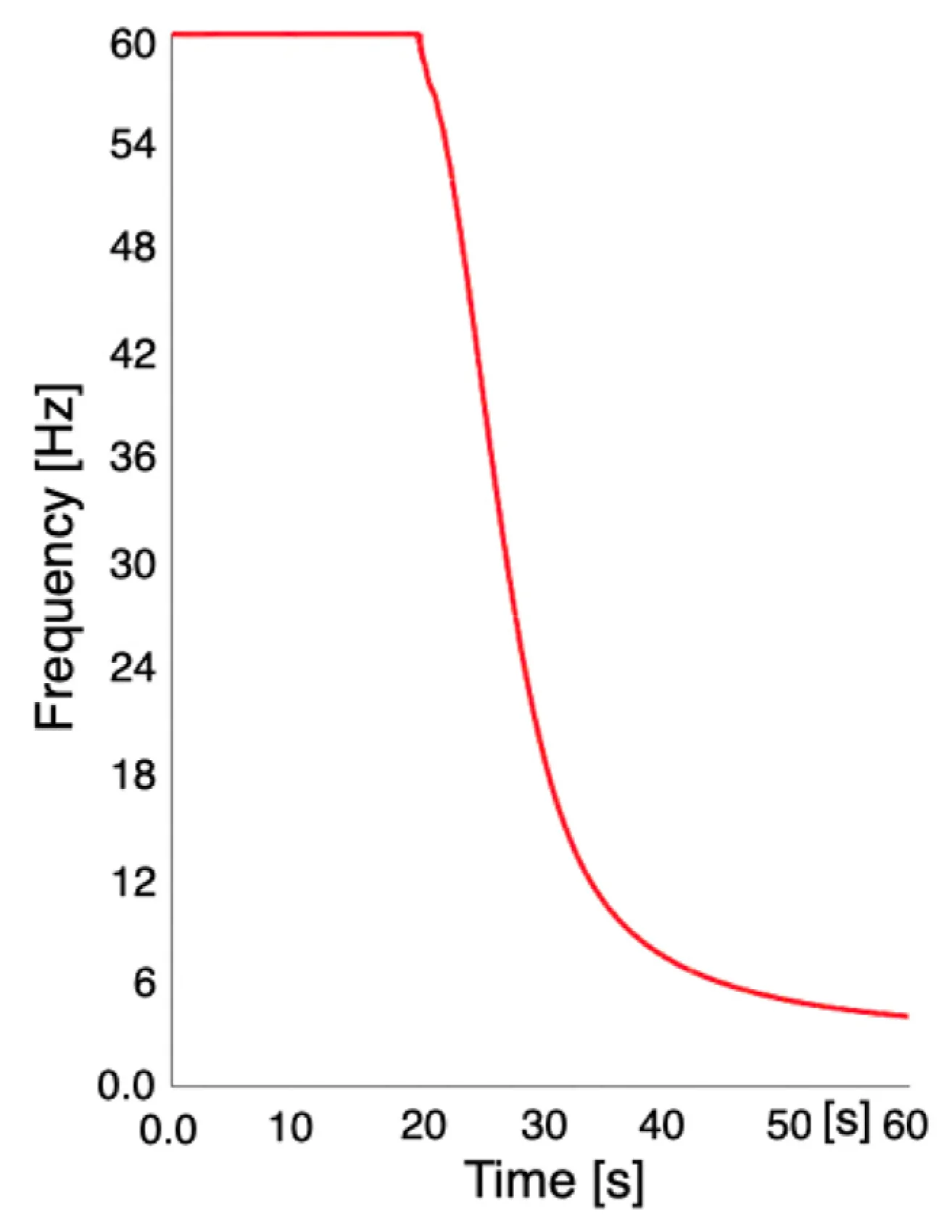
Frequency variation.

**Figure 24:**
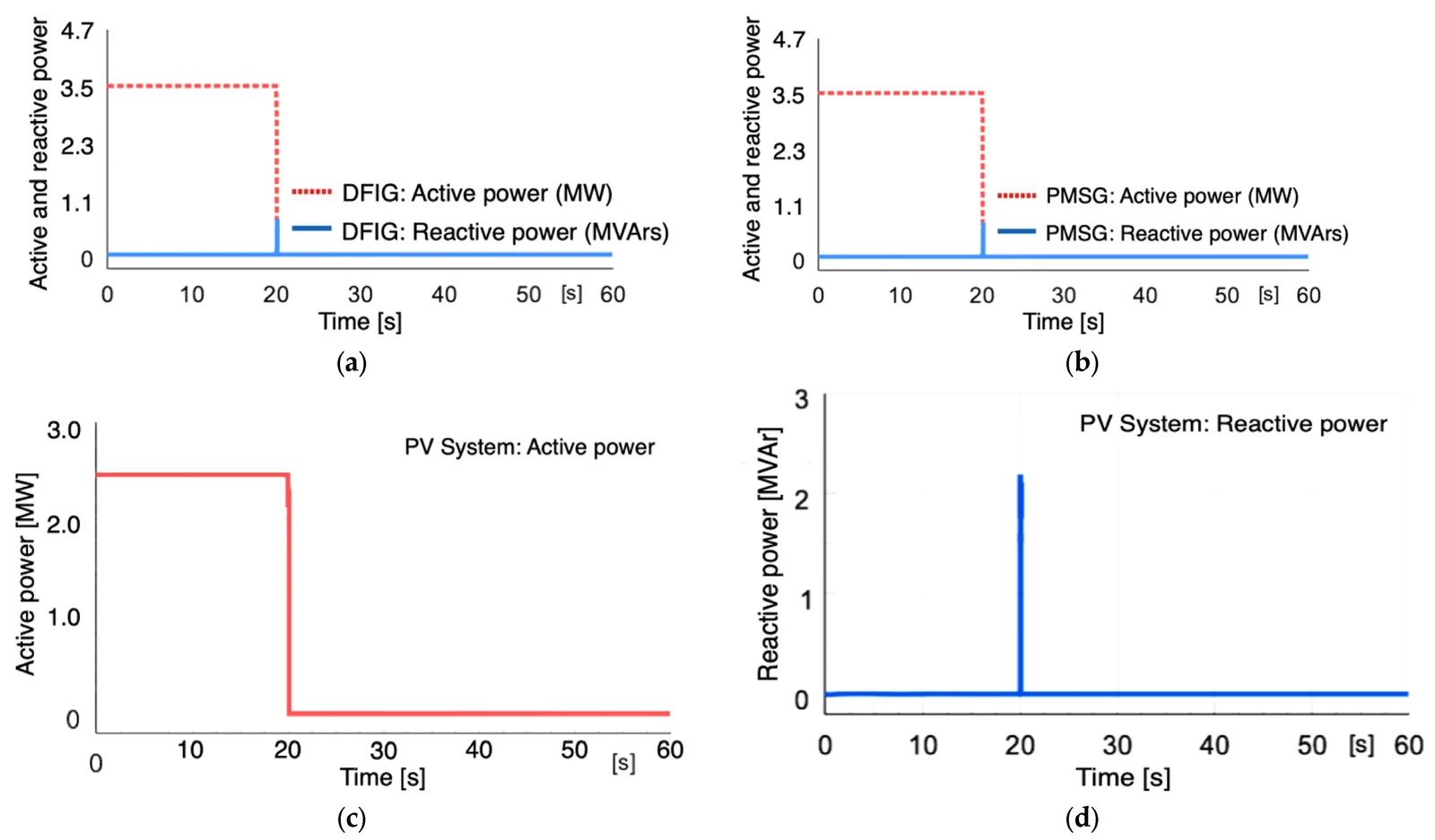
Variation in (**a**) DFIG output power, (**b**) PMSG output power, (**c**) active output power of the PV_1_ system, and (**d**) reactive output power of the PV_2_ system.

**Figure 25:**
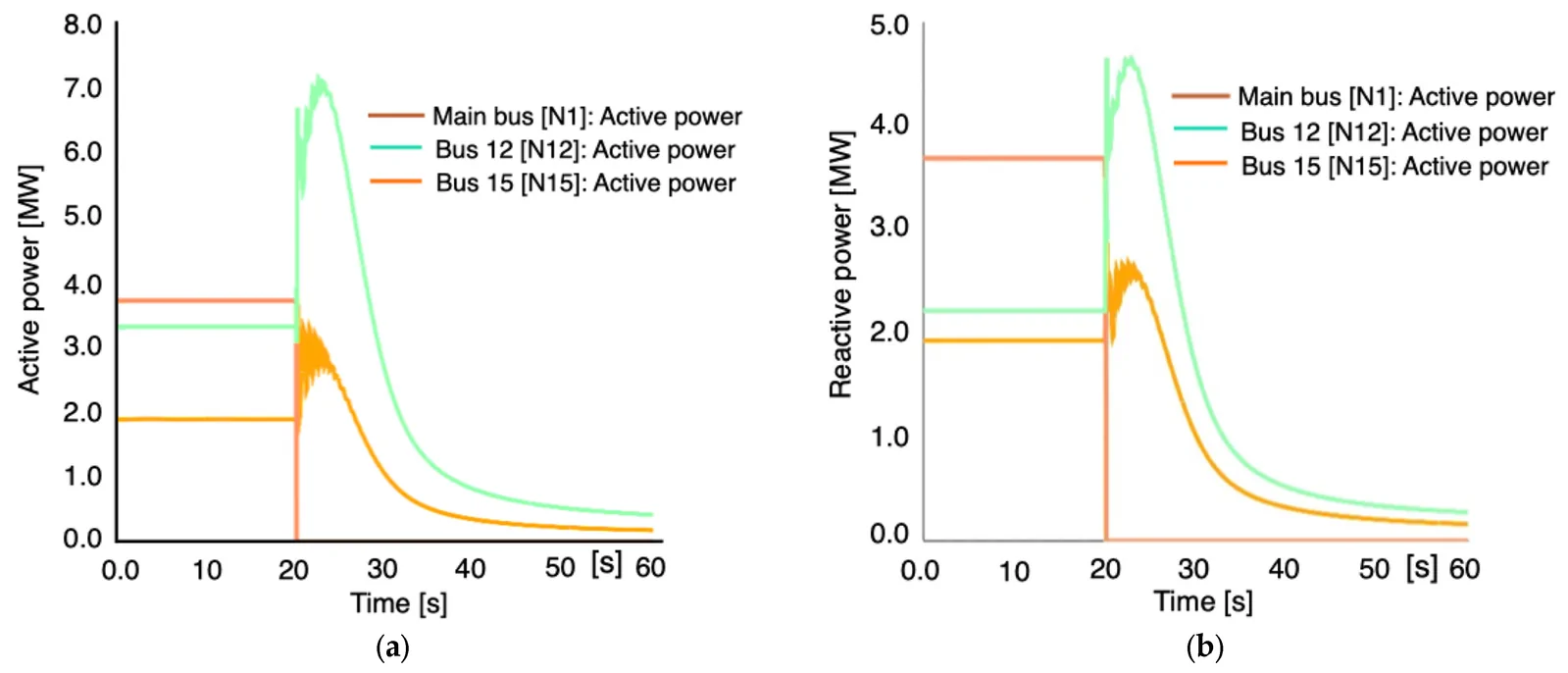
Variation in (**a**) active power and (**b**) reactive power of the SGs.

The simulation results are illustrated in Figure 22, Figure 23, Figure 24 and Figure 25. Although the hybrid microgrid remains stable in terms of rotor angles (Figure 22b), it experiences voltage drops (Figure 22a) and frequency drops (Figure 23) after the sudden shutdown of synchronous generator N15. This event, which occurs at 20 s, causes voltage and frequency to fall below the critical threshold of 0.9 p.u. (Figure 22a), leading to disconnection between the RESs and the main grid (Figure 24), thereby aggravating the shortage of electrical generation.

Although the governors of the remaining SGs, N11 and N16, rapidly adjust power generation to compensate for the loss of the PV systems, WTs, and synchronous generator N15, their reserve is insufficient to counteract the amount of lost power, leading to the inevitable collapse of frequency and voltage, as demonstrated by the simulation results.

To facilitate observation of the impact of the RESs (PV system and WTs) on the overall transient stability of the electric hybrid microgrid, Table 25 provides a summary of the key parameters used as indicators for this assessment.

From the perspective of transient stability, the literature suggests that the safe limit for RES integration in the hybrid microgrid is approximately 30%. Higher penetration levels could induce dynamic instability in microgrids after a three-phase short-circuit fault.

Consequently, increasing renewable participation would require mitigation measures such as modifying the grid topology, strengthening converter control, and integrating additional ESS capacity.

### 5.3. Results and Discussion

#### Progressive Degradation of Transient Stability with Increasing RES Penetration

Before evaluating the proposed controller, it is necessary to clearly establish the relationship between the increase in renewable penetration and the deterioration of transient-stability margins. For this purpose, the microgrid was evaluated under different levels of RES penetration, maintaining the system demand and progressively modifying the participation of the SGs. The redistribution of active power among diesel units, combined cycle generation, and RES is documented in Table 23. In particular, the renewable contribution increases from 1.05 MW at 10% penetration to 10.50 MW in the 100% scenario, while the contribution of the SGs decreases progressively until reaching a condition of zero physical inertia in the case of 100% RES.

This progressive replacement of synchronous generation with converter-based resources directly modifies the dynamic characteristics of the microgrid. As shown in Table 25, the system’s equivalent inertia decreases from 4.27 s in the non-renewable penetration case to 1.25 s in the 70% RES scenario. Simultaneously, the RoCoF increases from 1.23 to 0.88 Hz/s in the considered stable cases, while the frequency nadir decreases from 59.74 to 59.45 Hz. The most significant effect is observed in the CCT, which drops from 270 ms for the 0% RES case to only 10 ms for 50% RES. For 70% RES, the conventional system loses stability under the considered contingency.

These results show that the increase in renewable penetration not only alters the steady-state energy balance but also transforms the dynamic properties of the system. The decrease in synchronous inertia reduces the system’s ability to manage instantaneous power imbalances, and the lowering of the CCT restricts the available time to respond to severe disturbances. Therefore, the data in Table 25 represent a deterioration scenario in stability, serving as a reference to evaluate the effectiveness of the MPC–VSG–BESS framework in recovery.

### 5.4. Application of the MPC–VSG–BESS Framework

To maintain consistency with the control formulation presented in Section 4, the results are presented using VSG parameters, both normalized and dimensional. The MPC optimization variables are denoted $J_{n}$ y $D_{n}$, while the corresponding physical parameters of the VSG are obtained as $J_{v}\left(k\right)\;=\;J_{\mathrm{scale}}J_{n}(k)$ y $D_{v}\left(k\right)\;=\;D_{\mathrm{scale}}D_{n}(k)$. The normalized variables define the optimization space, while the dimensional parameters represent the actual virtual inertia and damping applied to the VSG controller.

To evaluate the effectiveness of the proposed control architecture—which considers energy efficiency—in conditions of high and ultrahigh penetration of renewable energies, the MPC–VSG–BESS scheme was applied to scenarios with penetration levels of 70%, 90%, and 100%. These cases represent a progressive reduction in the contribution of synchronous generation and consequently increasingly demanding requirements regarding converter-based synthetic inertia, damping support, and BESS energy management.

In the proposed architecture, the MPC operates on the normalized control variables $J_{n}$ y $D_{n}$, while the corresponding physical parameters of the VSG are obtained through the implemented scaling relationships ${J_{v}}^{*}\left(k\right)=\;J_{\mathrm{scale}}{J_{n}}^{*}\left(k\right)$ y ${D_{v}}^{*}\left(k\right)\;=\;D_{\mathrm{scale}}{D_{n}}^{*}\left(k\right)$.

The SoC-dependent energy control mechanism does not directly prescribe ${J_{n}}^{*}\left(k\right)$. Instead, it defines the permissible upper limit $J_{n\text{\_}\max}^{\mathrm{EA}}\left[\mathrm{SoC}\left(k\right)\right]$, within which the MPC determines the optimal inertia contribution based on the anticipated transient response, BESS operational constraints, and control objectives. Consequently:

(75) $$J_{n}^{*}\left(k\right)\leq J_{n\text{\_}\max}^{\mathrm{EA}}\left[\mathrm{SoC}\left(k\right)\right]$$

This distinction is essential for interpreting the following simulation results, as the level of renewable-energy penetration, the BESS SoC, the permissible inertia limit, and the MPC’s instantaneous decision represent different magnitudes.

#### 5.4.1. Case Study 6: Main Generator Fault—Renewable Penetration = 70% Applying MPC–VSG–BESS

In this scenario, the hybrid microgrid operates with three SGs (N1, N12, and N15) along with the renewable resources and BESS defined for the operational condition of 70% renewable-energy penetration. Two control configurations are evaluated: (i) optimization based solely on MPC, in which the parameters of the predictive control are optimized while the BESS controller maintains its reference configuration; and (ii) coordinated MPC–VSG–BESS optimization, in which both the predictive control variables and the BESS control parameters are optimized. The goal of this comparison is to isolate the contribution of the coordinated BESS control to the transient response. These results are shown in Table 26, Table 27 and Table 28.

**Figure 26:**
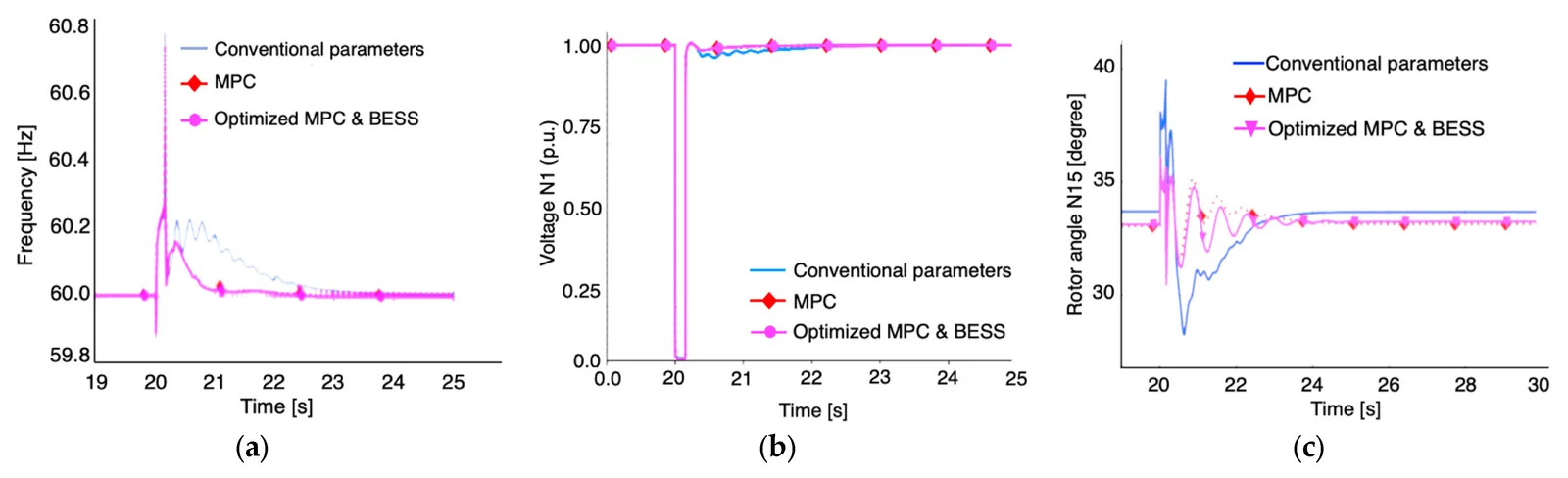

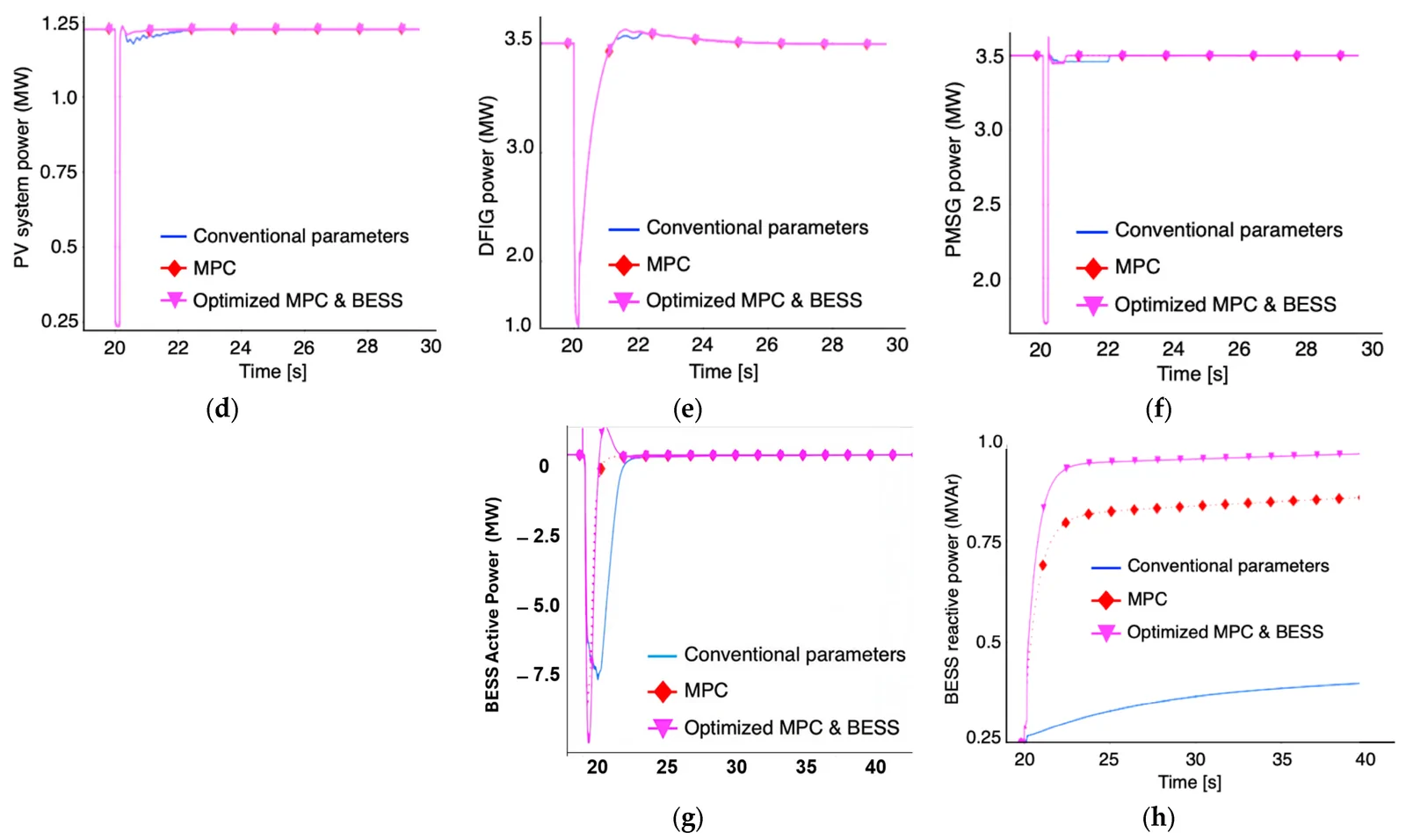
Variation in (**a**) frequency, (**b**) voltage N15, (**c**) rotor angle N15, (**d**) PV power at N1, (**e**) DFIG power, (**f**) PMSG power, (**g**) BESS active power, and (**h**) BESS reactive power.

**Figure 27:**
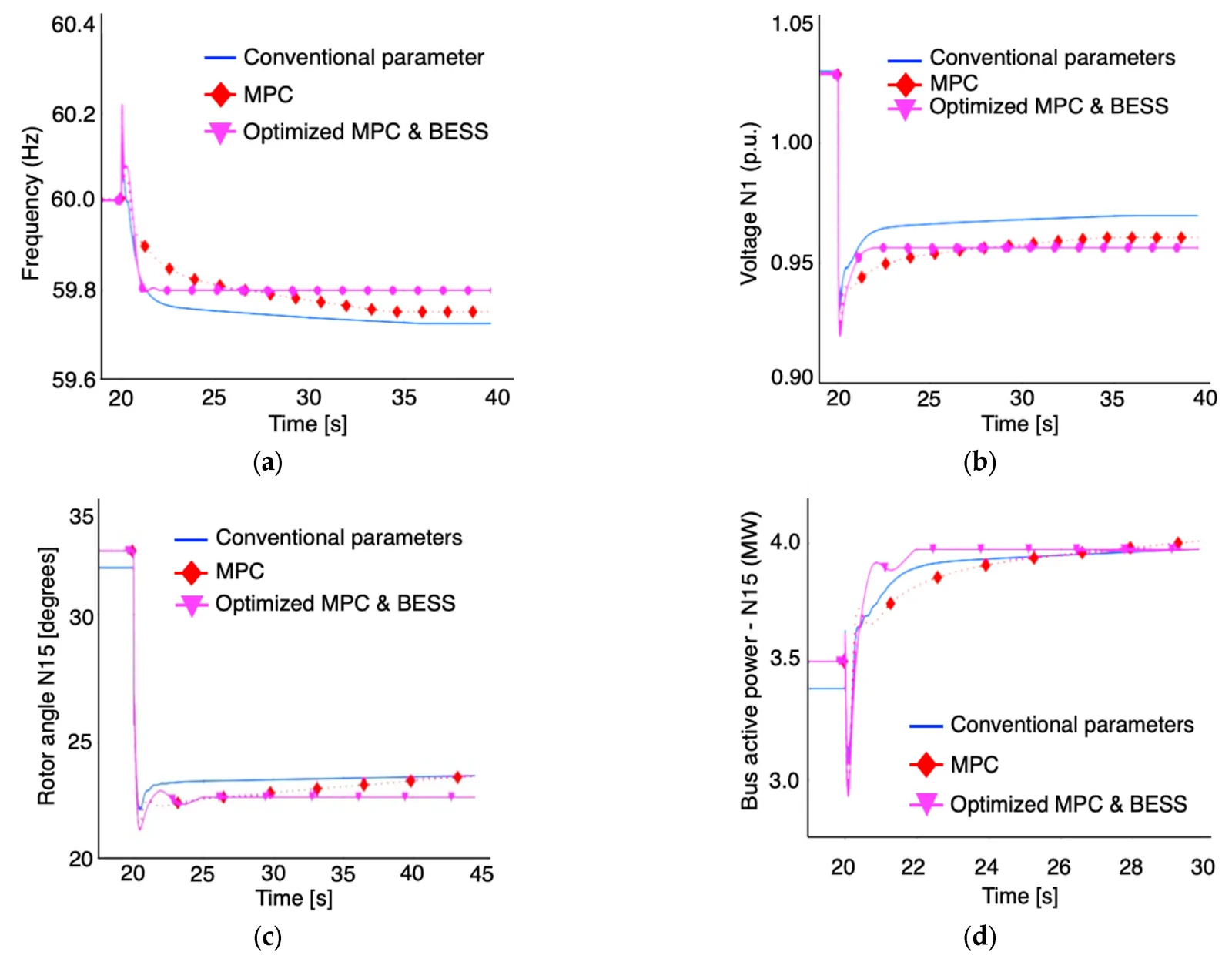

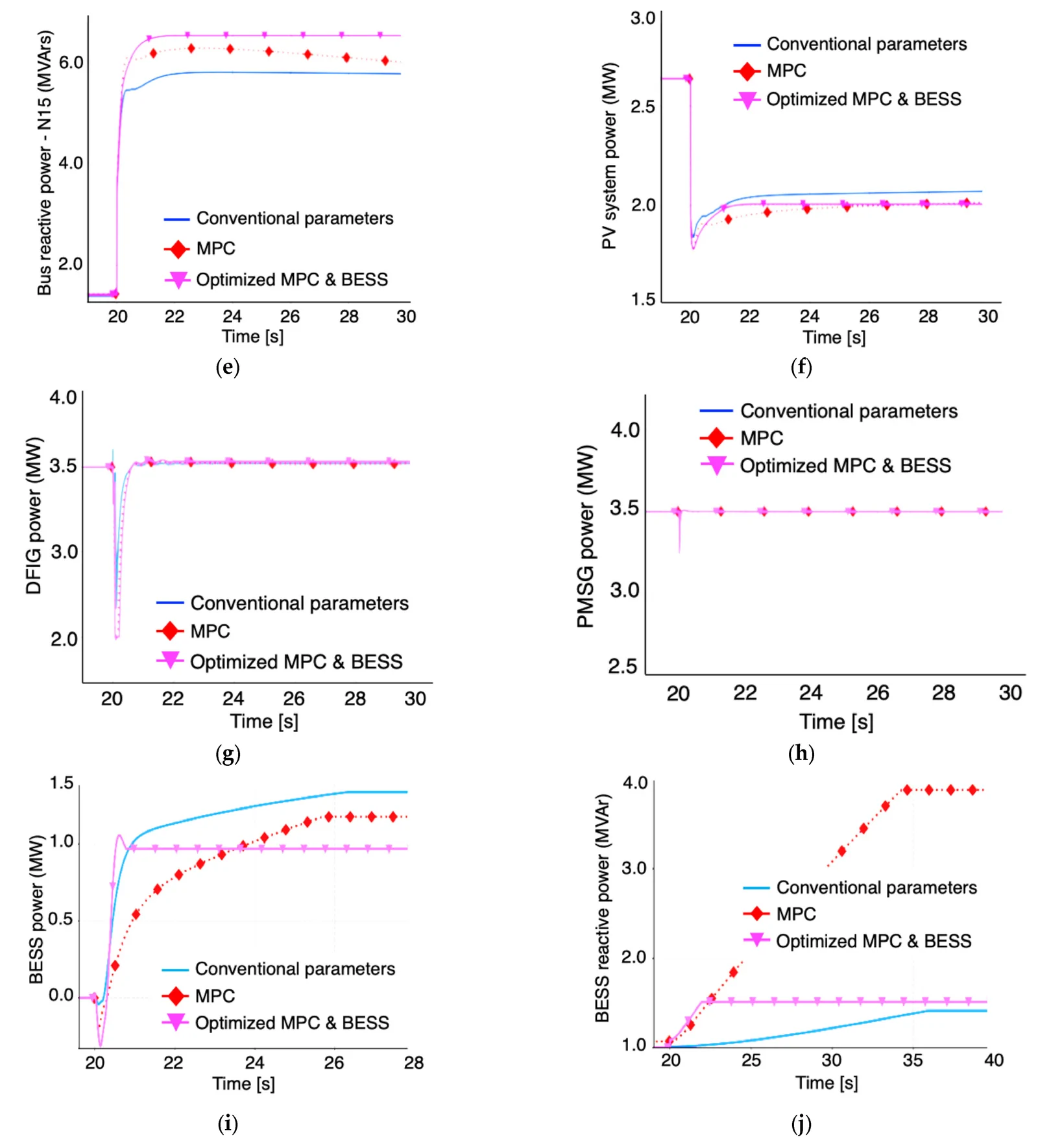
Variation in (**a**) frequency, (**b**) voltage N15, (**c**) rotor angle N15, (**d**) bus active power N15, (**e**) bus reactive power N15, (**f**) PV system power, (**g**) DFIG power, (**h**) PMSG power, (**i**) BESS active power, and (**j**) BESS reactive power.

Figure 26 shows the response of the proposed electric hybrid microgrid after a three-phase short circuit at node N1, while Figure 27 illustrates the transient behavior of the microgrid during the disconnection of generator N12. Figure 26 and Figure 27 reveal several important findings, as follows.

- The transient period of the proposed hybrid microgrid is significantly reduced with the optimal BESS and MPC controller parameters, particularly with respect to frequency and voltage stabilization after a three-phase short circuit. The transient time decreases from more than 4 s with the default parameters to only 1 s when the optimal parameters are applied.
- Improvements in minimum and maximum frequencies, as well as in rotor angle, are particularly evident during the transient process, especially after the three-phase short circuit. The frequency decreases from 60.78 Hz with the default parameters to 60.73 Hz with the optimized MPC configuration, and to 60.27 Hz when both the MPC controller and the BESS are optimized. The minimum frequencies are 59.88 Hz, 59.90 Hz, and 59.90 Hz, respectively. The maximum rotor angle for N15 decreases from 39.47° with the default configuration to 36.00° with the optimized parameters. This reduction is crucial in other scenarios, presented later, where these values approach their operating limits.
- To stabilize the hybrid microgrid after these incidents, the minimum required active and reactive power from the BESS is 0.75 MVA and 1.5 MVA, respectively, for the optimized MPC configuration and the optimized MPC and BESS controller parameters. The coordinated MPC–BESS optimization reduces the power-support demand required from the BESS while maintaining the desired transient response, indicating a more efficient utilization of the available storage resource, which is essential not only from a cost perspective but also for technical efficiency.

The distinction between the normalized MPC variables and the physical parameters of the VSG is important for interpreting the simulation results. The values ${J_{n}}^{*}$ and ${D_{n}}^{*}$ represent the normalized optimization variables selected by the MPC, while ${J_{v}}^{*}$ and ${D_{v}}^{*}$ represent their corresponding physical values applied to the VSG through the scale relations ${J_{v}}^{*}=\;J_{\mathrm{scale}}{J_{n}}^{*}$ y ${D_{v}}^{*}=\;D_{\mathrm{scale}}{D_{n}}^{*}$. The energy-sensitive and SoC-dependent function does not directly prescribe superscript base, ${J_{n}}^{*}$. Instead, it sets an allowable upper limit $J_{n\text{\_}\max}^{\mathrm{EA}}$ (SoC).

Consequently, the optimized MPC solution can vary within this feasible region depending on the expected frequency dynamics, the severity of the disturbance, and the operational constraints of the BESS.

#### 5.4.2. Case Study 7: Main Generator Fault—Renewable Penetration = 90% Applying MPC–VSG–BESS

**Figure 28:**
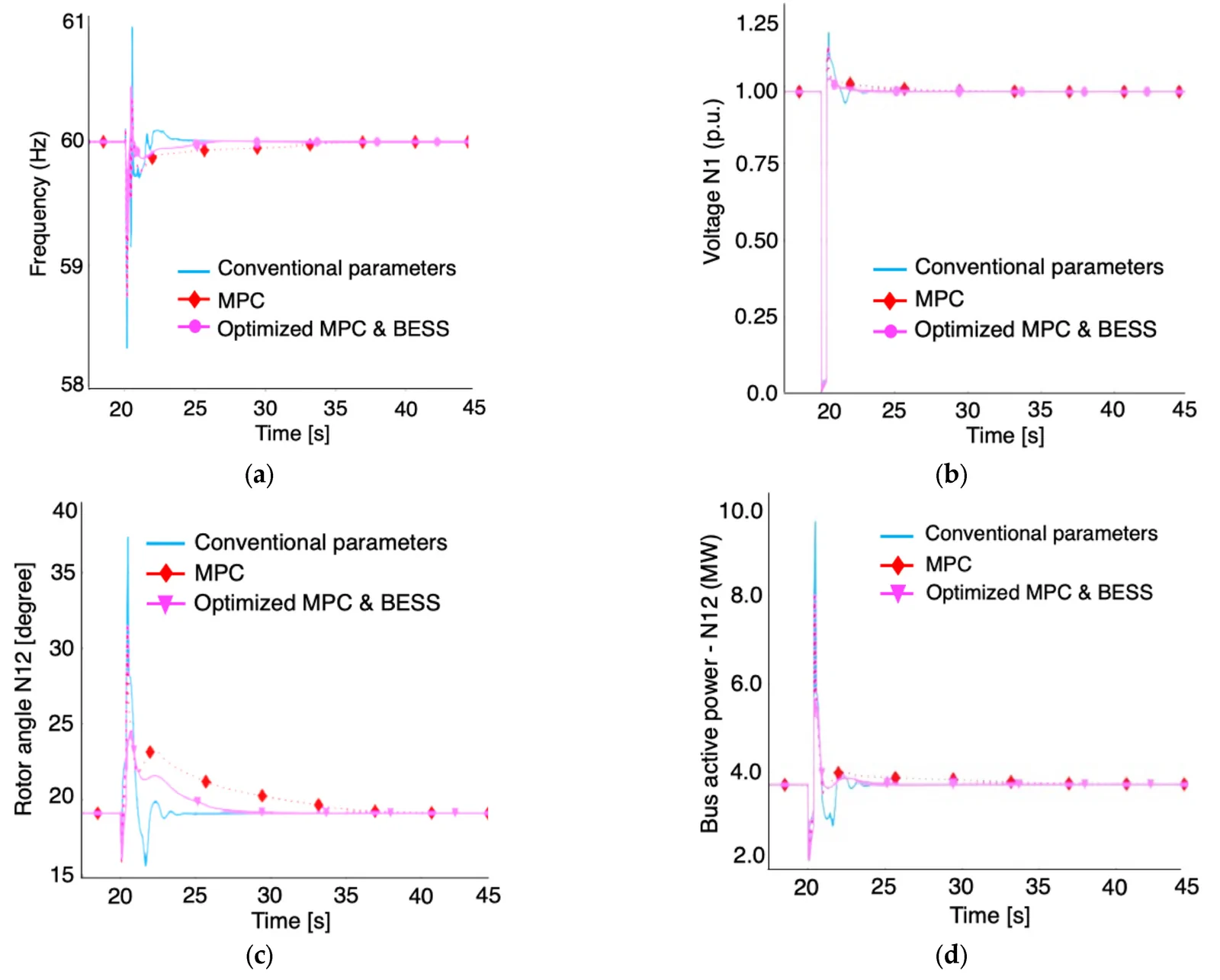
Variation in (**a**) frequency, (**b**) voltage at N1, (**c**) rotor angle of N12, and (**d**) active power at N12.

**Figure 29:**
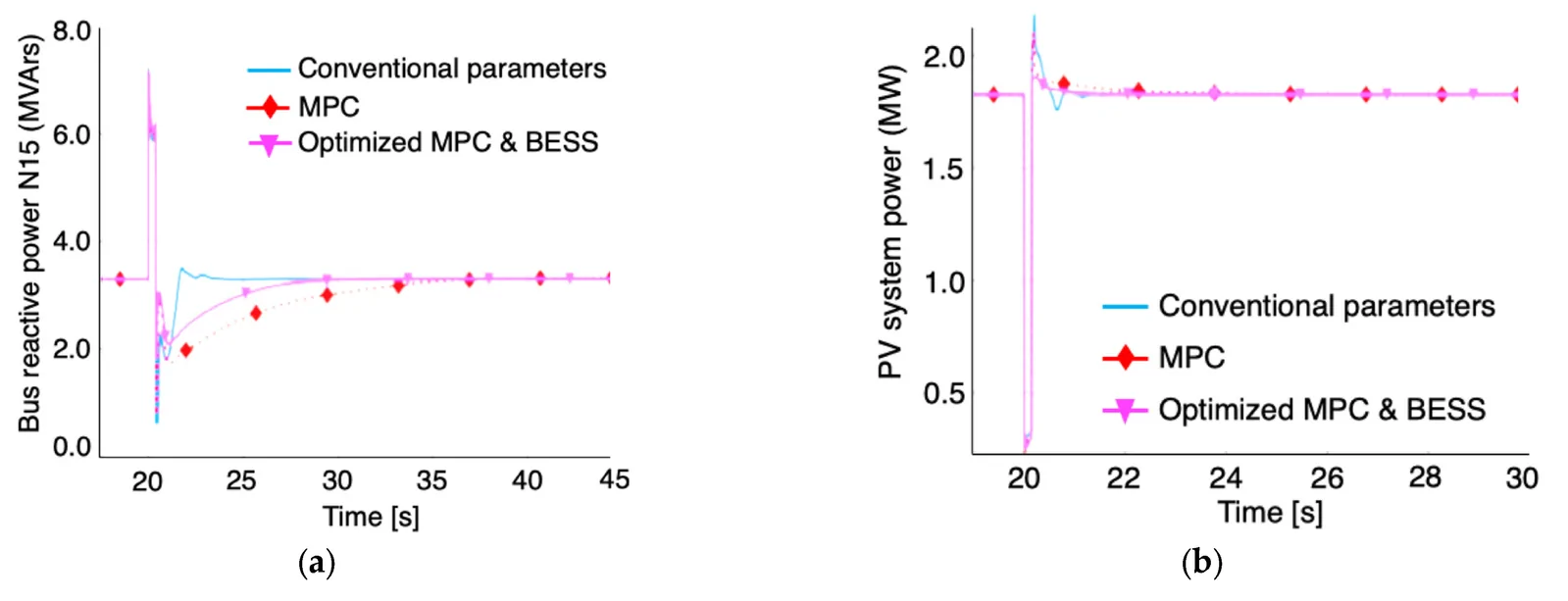

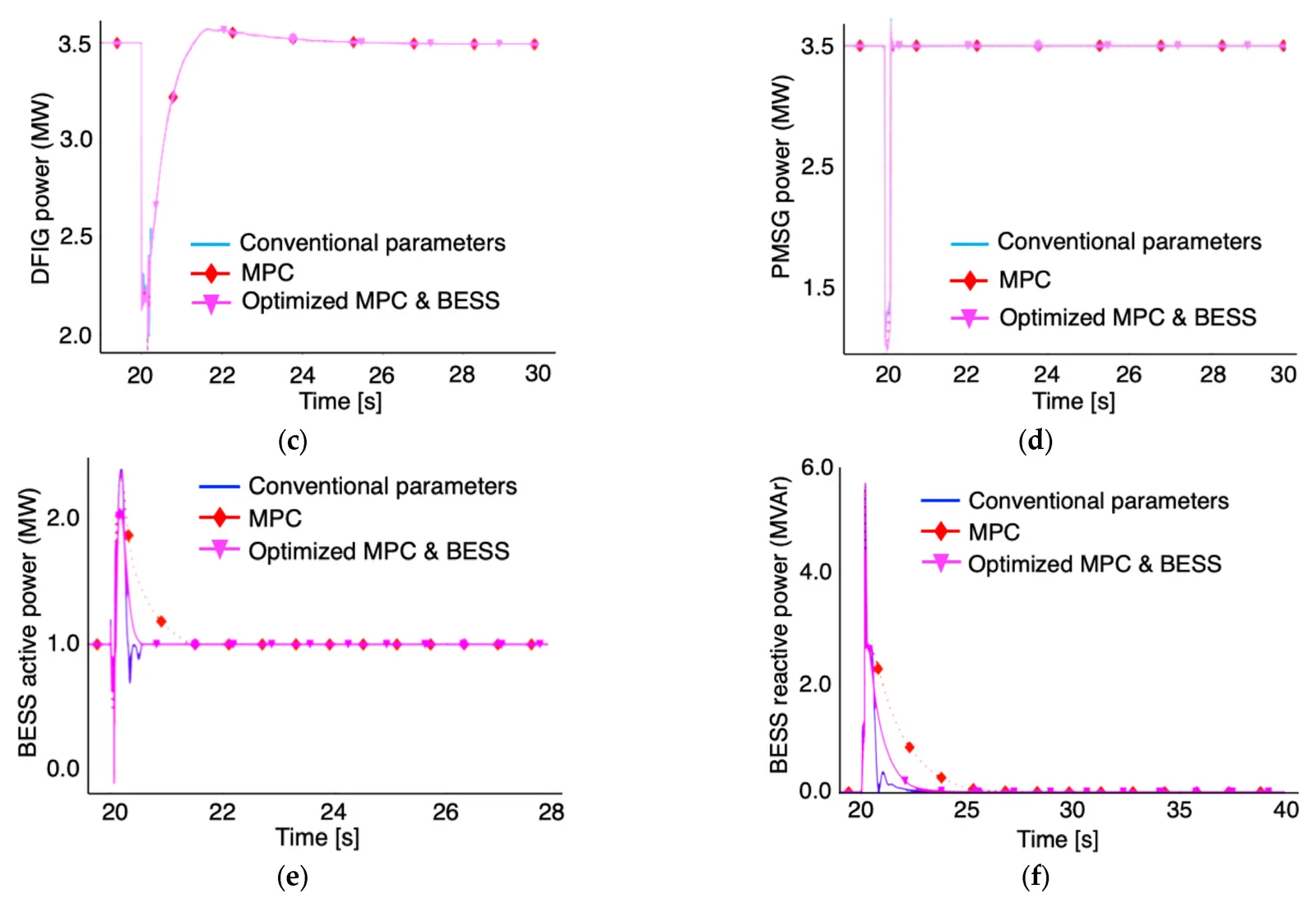
Variation in (**a**) reactive power in N12, (**b**) PV power in N1, (**c**) DFIG power, (**d**) PMSG power, (**e**) BESS active power, and (**f**) BESS reactive power after the three-phase short circuit.

**Figure 30:**
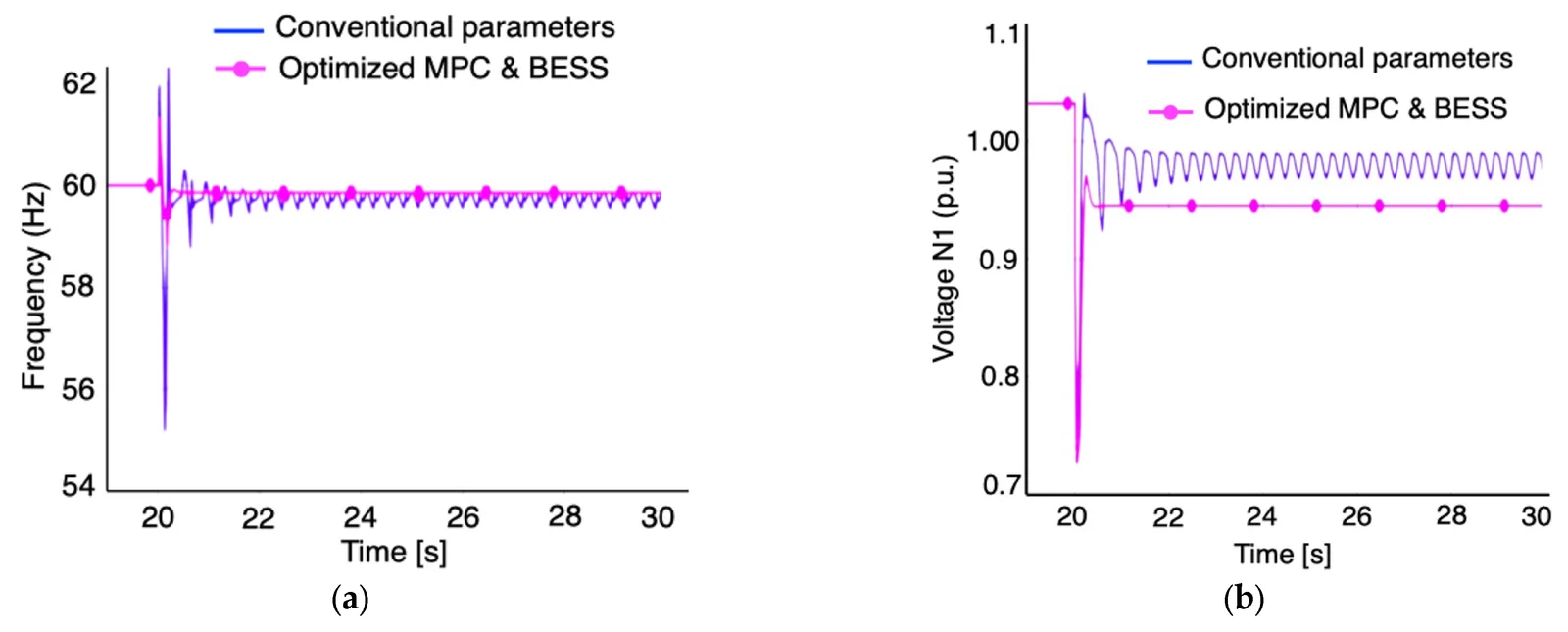
Variation in (**a**) frequency and (**b**) voltage at N1 after the output of synchronous generator at N12.

**Figure 31:**
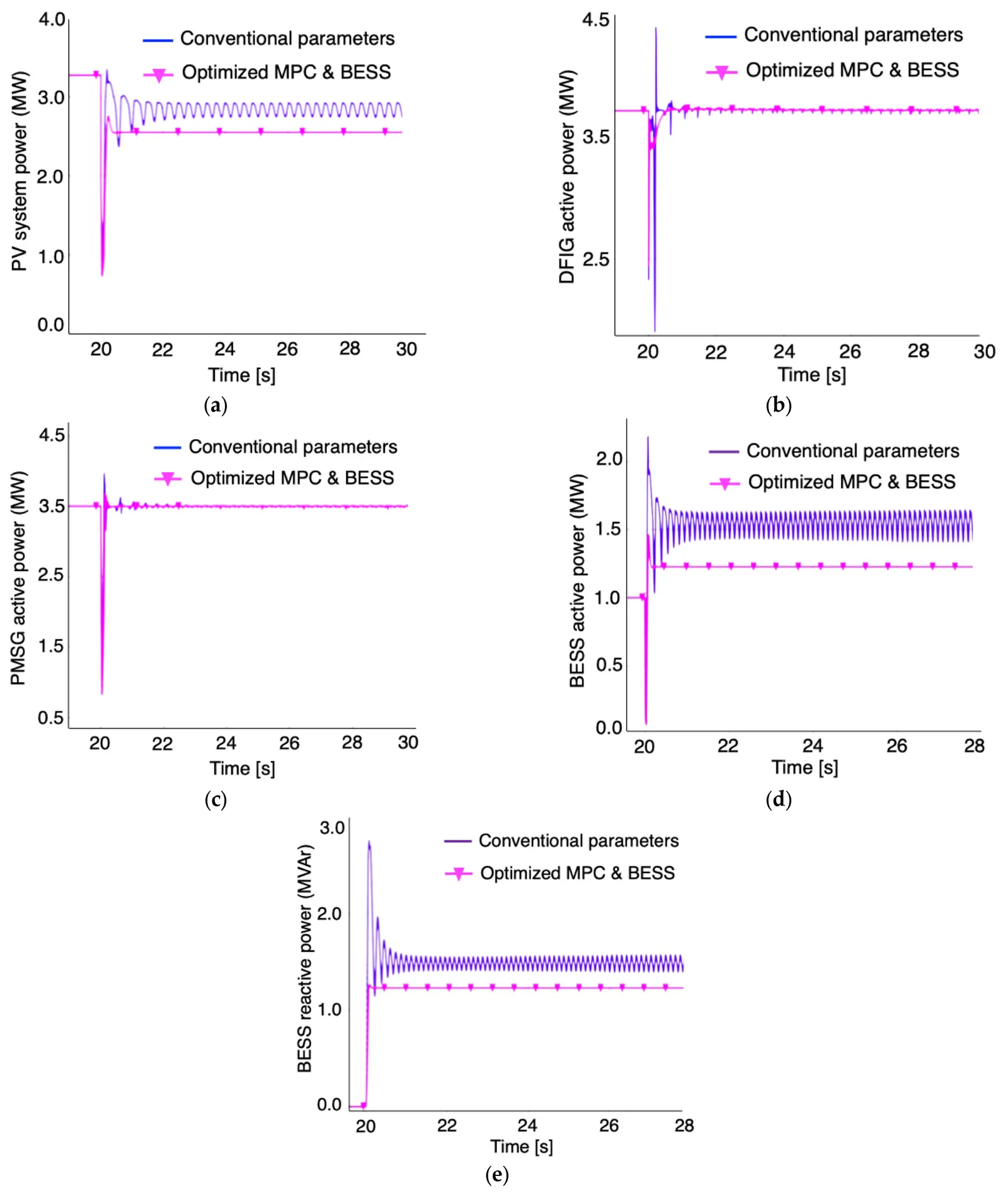
Variation in (**a**) PV system power at N1, (**b**) DFIG power, (**c**) PMSG power, (**d**) BESS active power, and (**e**) BESS reactive power after the disconnection of synchronous generator N12.

In this scenario, only synchronous generator N12 is operational. Similar to the previous section, applying MPC control helps identify the optimal BESS controller parameters, as indicated in Table 29 and Table 30. Figure 28, Figure 29, Figure 30 and Figure 31 compare the dynamic response of the proposed hybrid microgrid after a three-phase short circuit at node N1 and the sudden disconnection of synchronous generator N12 in several cases.

The effectiveness of applying MPC-derived optimal parameters to the BESS controller is clearly demonstrated in this case study in several key areas, as follows.

- **Frequency:** During a three-phase short circuit at node N1, the minimum and maximum frequencies are 58.33 Hz and 60.92 Hz, respectively, with the default parameters. On the other hand, with optimal parameters obtained using MPC, these values improve to 59.76 Hz and 60.44 Hz, and further to 59.26 Hz and 60.32 Hz when the MPC and BESS controller are applied optimally, as shown in Figure 28. It should be noted that during the outage of the SG connected at node N12, frequency stabilization is achieved only when the BESS is optimally configured (see Figure 30). The coordinated MPC–BESS setup significantly reduces the frequency excursion compared to the default controller. Although the minimum frequency with the fully coordinated setup is lower than that achieved through exclusive optimization with MPC, the coordinated strategy provides the stabilization needed after contingencies, especially during the disconnection of the SG. This result indicates that the goal of the coordinated controller is not simply to maximize the minimum instantaneous value of the frequency nadir, but to achieve a feasible transient response that simultaneously satisfies the operational constraints of the BESS.
- **Voltage:** The voltage response provides stronger evidence of the advantages of coordinated control. The maximum voltage deviation decreases from 1.17 p.u. with the default configuration to 1.13 p.u. through MPC optimization and to 1.02 p.u. with MPC–BESS coordinated control. In this way, the coordinated setup significantly reduces the voltage deviation after the fault and keeps the response below the upper operational threshold of 1.15 p.u. considered in the simulation.
- **Rotor angle:** As with the improvements in frequency and voltage, optimization of the MPC and BESS parameters is the most effective way to limit rotor-angle fluctuations in the SGs, as indicated in Figure 28c. This improves the transient stability, security, and reliability of the system.
- **Other aspects:** The optimal parameters obtained from the MPC for the BESS controller ensure stable operation of the hybrid-microgrid components after the outage of synchronous generator N12 (see Figure 31) and significantly reduce fluctuations after a three-phase short circuit, as shown in Figure 28 and Figure 29. Such stability cannot be achieved with the default configuration or by optimizing only the MPC, especially in the case of the SG N12 outage. In the event of the outage of synchronous generator N12, the hybrid microgrid depends on the BESS as the reference node to maintain operation of the electric hybrid microgrid, making BESS optimization essential for stabilizing the microgrid variables after disturbances. In addition, the installed BESS capacity is 1/2 MVA for both the default and optimal power-support system parameters, but decreases to 0.4 MVA when both the MPC and the BESS are optimized, as shown in Figure 31e. This demonstrates that using optimal parameters for both the MPC and the BESS not only stabilizes the electric microgrid but also reduces the required installed BESS capacity.

The scenario of 90% renewable penetration demonstrates the importance of coordinated support through BESS more clearly than the 70% scenario. With only one SG remaining, the available physical inertia and frequency support based on speed regulators become insufficient after the generator disconnects. Consequently, the BESS becomes the predominant resource for fast response, while the MPC determines its operational trajectory and the VSG mechanism provides the synthetic electromechanical response. The resulting behavior highlights the complementary nature of prediction, virtual inertia, and energy storage, rather than suggesting that any of these elements alone is sufficient.

#### 5.4.3. Case Study 8: Main Generator Fault—Renewable Penetration = 100% Applying MPC–VSG–BESS

The case of 100% renewable-energy penetration represents the most critical operational condition considered in this study, as the SGs are not in operation. Consequently, the microgrid lacks physical rotational inertia, and its transient behavior depends entirely on the connected resources via converters and the grid formation reference provided by the BESS. Under this condition, the BESS sets the frequency and voltage reference during the transient event, while the proposed MPC–VSG coordination regulates the support of synthetic inertia and damping available based on the instantaneous operational state and energy constraints.

**Figure 32:**
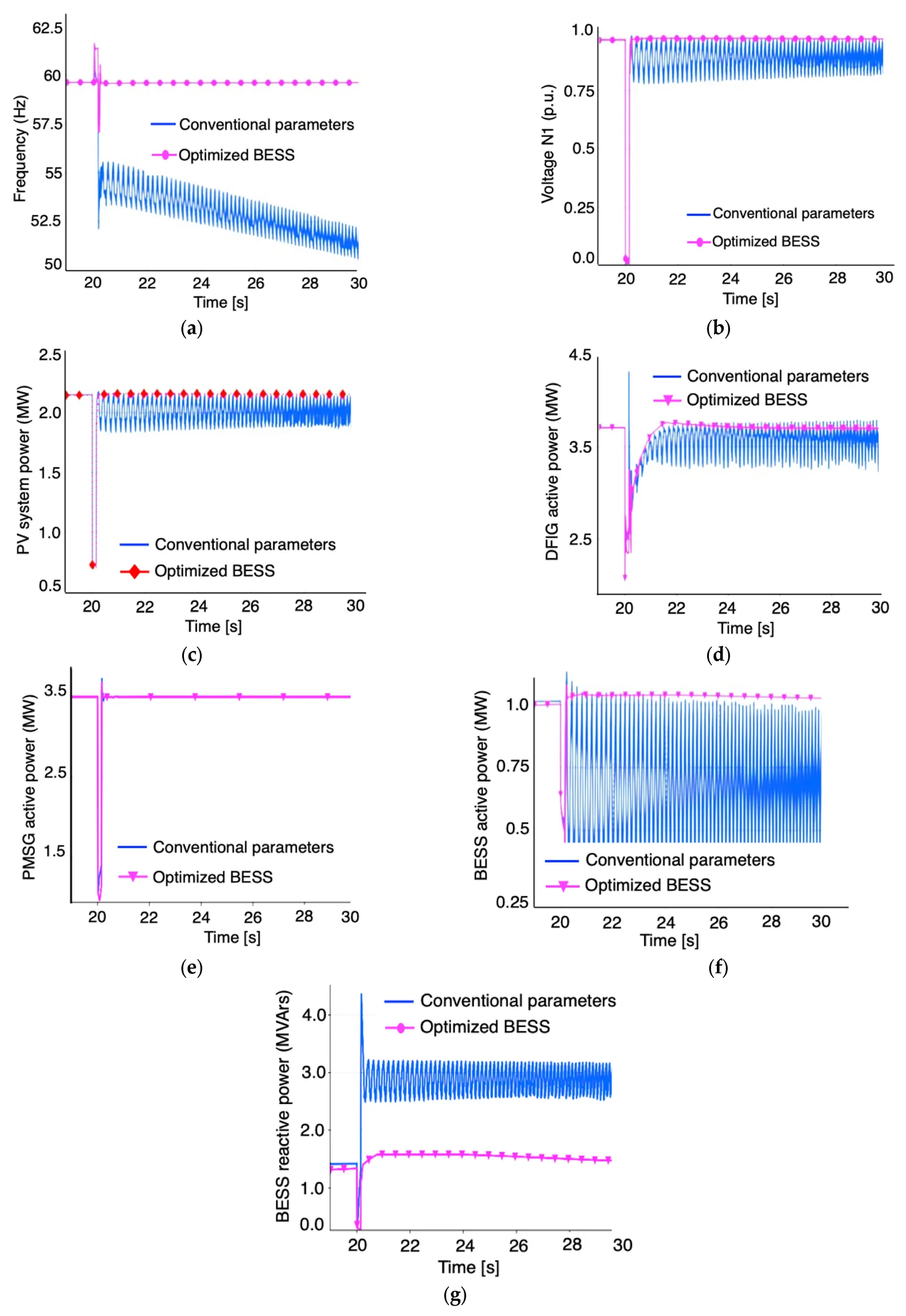
Variation in (**a**) frequency, (**b**) N1 voltage, (**c**) PV system power, (**d**) DFIG power, (**e**) PMSG power, (**f**) BESS active power, and (**g**) BESS reactive power after the short circuit.

The results of the simulation in Figure 32 demonstrate that the proposed architecture remains capable of establishing and maintaining the electrical reference during the three-phase fault. This result is particularly relevant because the 100% RES case represents an operating condition with zero physical inertia in which neither the inertia nor the regulator response typical of conventional SGs are available. The results of case studies 6–8 are summarized in Table 31.

### 5.5. Results and Discussion

#### 5.5.1. Performance of the MPC–VSG–BESS Framework Under High-RES Penetration

Once the progressive degradation of the conventional system was established, the behavior of the MPC–VSG–BESS framework was evaluated for renewable penetration of 70%, 90%, and 100%. These three scenarios represent progressively more demanding conditions, characterized by a reduction in synchronous generation and therefore greater dependence on synthetic inertia and the fast support provided by the BESS. In this evaluation, the MPC optimizes the normalized variables $J_{n}$ and $D_{n}$, while the physical parameters applied to the VSG are obtained through the scale relationships $J_{v}=J_{\mathrm{scale}}J_{n}$ and $D_{v}=D_{\mathrm{scale}}D_{n}$. It is important to highlight that the energy awareness mechanism does not directly prescribe ${J_{n}}^{*}$, but instead modifies the allowable upper limit $J_{n\text{\_}\max}^{\mathrm{EA}}\left[\mathrm{SoC}\left(k\right)\right]$, within which the MPC determines the optimal action.

##### 70% RES

For the 70% RES scenario, the comparison between conventional parameters, the optimized configuration using MPC, and the coordinated MPC–BESS configuration shows a progressive improvement in transient response. With conventional parameters, the frequency reaches a maximum of 60.78 Hz and a minimum of 59.88 Hz. The application of MPC reduces the maximum to 60.73 Hz and produces a minimum of 59.90 Hz, while the coordinated MPC–BESS optimization further reduces the maximum to 60.27 Hz, maintaining the minimum at 59.90 Hz. At the same time, the maximum rotor angle of N15 decreases from 39.47° to 36.00°.

The result is particularly significant when considering the magnitude of the maximum deviation with respect to the nominal frequency. For the conventional case:

$$\Delta f_{\max\text{\_}\mathrm{conv}}=60.78-60=0.78\;\mathrm{Hz}$$

while for the MPC–BESS configuration:

$$\Delta f_{\max,\;\mathrm{MPC}–\mathrm{BESS}}=60.27-60=0.27\;\mathrm{Hz}$$

Therefore, the relative reduction of the maximum frequency excursion is:

$$\eta_{\Delta f\text{\_}\max}=\frac{0.78-0.27}{0.78}\times 100=65.4\%$$

Likewise, the reduction in maximum rotor angle is:

$$\eta_{\delta }=\frac{39.47-36.00}{39.47}\times 100=8.8\%$$

These results indicate that the contribution of the BESS is not limited to directly compensating for power imbalance, but when coordinated with the adaptation of *J* and *D*, it favorably modifies the system’s dynamic trajectory. Additionally, the transient period is reduced from over 4 s with conventional parameters to approximately 1 s with optimized parameters.

##### 90% RES

The 90% RES scenario represents a condition of very low physical inertia. Under conventional parameters, the frequency ranges between 58.33 and 60.92 Hz. With MPC optimization, these values improve to approximately 59.76 and 60.44 Hz, respectively. The MPC–BESS coordination provides an additional response, with a frequency interval of about 59.26–60.32 Hz.

The maximum deviation from the nominal frequency changes from:

$$\Delta f_{\mathrm{nadir}\text{\_}\mathrm{conv}}=60-58.33=1.67\;\mathrm{Hz}$$

to:

$$\Delta f_{\mathrm{nadir},\;\mathrm{MPC}–\mathrm{BESS}}=60-59.26=0.74\;\mathrm{Hz}$$

Therefore:

$$\eta_{\mathrm{nadir}}=\frac{1.67-0.74}{1.67}\times 100=55.7\%$$

Similarly, the maximum frequency increase decreases from 0.92 Hz in the conventional case to 0.32 Hz with MPC–BESS:

$$\eta_{\max}=\frac{0.92-0.32}{0.92}\times 100=65.2\%$$

These results show that the benefit of the proposed framework remains even when renewable penetration reaches 90%, a condition in which the available synchronous generation is considerably lower. The combined effect of predictive inertia adaptation and fast support from the BESS allows for simultaneous reduction of both the frequency nadir and the overshoot after the disturbance.

##### 100% RES and Zero Physical Inertia

The 100% RES scenario represents the most demanding case considered in this study, due to the absence of SGs and consequently conventional physical inertia. Under these conditions, the conventional controller fails to maintain the frequency within the safety limit: the frequency drops to 56.78 Hz, falling below the UFLS threshold of 57.50 Hz. In contrast, the MPC–VSG–BESS controller limits the nadir to 59.26 Hz and maintains the RoCoF at −0.32 Hz/s.

The magnitude of the frequency deviation from 60 Hz decreases by:

$$\Delta f_{\mathrm{conv}}=60-56.78=3.22\;\mathrm{Hz}$$

to:

$$\Delta f_{\mathrm{MPC}–\mathrm{VSG}\text{–}\mathrm{BESS}}=60-59.26=0.74\;\mathrm{Hz}$$

As a result, the relative reduction of the nadir deviation reaches:

$$\eta_{\mathrm{nadir}}=\frac{3.22-0.74}{3.22}\times 100=77.0\%$$

This result constitutes one of the primary pieces of quantitative evidence regarding the proposed framework’s ability to operate under conditions of zero physical inertia. Furthermore, the controller adapts the virtual parameters during the contingency. The maximum observed value of virtual inertia reaches $J_{v}=48.5\;\mathrm{kg}\cdot m^{2}$, while virtual damping increases to $D_{v}=38.2$ Nm · s/rad. The initial increase in $J_{v}$ enables a synthetic inertial response during the initial rise in RoCoF, whereas the subsequent increase in $D_{v}$ helps to damp power and frequency oscillations. The reported settling time is less than 1.82 s.

##### CCT Enhancement

Evaluation through a three-phase ground short circuit provides a second independent measure of stability improvement. In the case of 100% RES, the conventional configuration shows an almost instant collapse, corresponding to a CCT of 0 ms. With the MPC–VSG–BESS framework, the CCT increases to 185 ms. For the 50% RES scenario, a CCT of up to 245 ms is also reported.

A percentage of improvement relative to the 0 ms case is not calculated because a relative variation with a zero denominator is mathematically undefined. Instead, the result is expressed as an absolute extension of the fault clearance margin of 185 ms in the 100% RES scenario.

This result should be interpreted together with the definition of CCT used in the manuscript: the three-phase fault is applied at the primary distribution bus of 4.16 kV, while the response variables are monitored at the 13.8 kV PCC. Therefore, the voltage level at the fault point and the measurement voltage level do not represent the same electrical bus.

Based on the above results, Table 32 presents a quantitative comparison between the different control configurations.

## 6. Critical Discussion of Related Works

To contextualize the contributions of the proposed MPC-driven energy-aware adaptive VSG framework, this section evaluates state-of-the-art transient-stability control strategies published in recent years. Recent literature focused on ultrahigh-penetration RES-based hybrid microgrids is generally divided into three paradigms: (i) fixed or heuristic adaptive VSG schemes, (ii) conventional MPC frameworks for frequency regulation, and (iii) BESS-centered energy-management strategies.

### 6.1. Fixed and Heuristic Adaptive VSG Paradigms

The earliest implementations of virtual SGs were mainly based on fixed virtual inertia (*J*) and damping (*D*) coefficients. Although they were effective with low-RES penetration (10% to 30%), fixed topologies could not adapt to the nonlinear dynamics of inverter-dominated grids, where total system inertia $\left(H_{\mathrm{sys}}\right)$ was reduced to zero.

To solve this problem, several heuristic self-tuning techniques emerged. Fuzzy-logic controllers and alternating-inertia architectures were proposed to adaptively modify the parameters according to the magnitude of the frequency deviation (Δω) and its derivative (dω/dt).

Although these heuristic methods decouple *J* and *D* during standard load steps, they lack mathematical optimization. They cannot predict future states over a finite horizon, which frequently generates underdamped post-fault oscillations. In addition, they treat the primary energy source as an ideal DC bus, completely ignoring the electrochemical current and SoC limits of the BESS cells.

### 6.2. Standard MPC in Grid-Forming Inverters

With the rise of high-speed digital signal processors, MPC has been widely adopted for primary and secondary frequency control. Linear-quadratic MPC frameworks have been implemented to solve online optimization routines, successfully reducing frequency-nadir and RoCoF peaks under small generation-load mismatches.

However, applying standard MPC to grid-forming converters introduces specific computational and structural challenges. Nonlinear MPC configurations that capture the complete SG dynamics require substantial online execution windows, making them impractical for real-time applications with fast sampling (*T_s_* ≤ 10 ms). To avoid this, researchers usually linearize grid models around highly generalized operating points, ignoring localized transient voltage–frequency coupling factors.

Standard predictive strategies omit transient degradation of the limit of the underlying hardware storage medium. In a 100% renewable microgrid, applying aggressive virtual-*J* injections without monitoring the instantaneous BESS polarization voltage V_p_ or SoC parameters can cause deep depletion of the internal cells. This entails the risk of activating low-voltage converter disconnection during the fault ride-through period, potentially causing catastrophic cascading blackouts.

### 6.3. BESS-Conscious and Degradation-Aware Microgrid Controls

To protect the life cycle of electrochemical assets, recent studies have integrated health metrics and dynamic SoC recovery mechanisms into microgrid dispatch loops. These strategies adjust active-power setpoints as functions of depth-of-discharge (DoD) limits and thermal-management parameters.

These BESS-centered controls operate exclusively at the tertiary or secondary control layers, using long dispatch intervals (from minutes to hours). Consequently, they are structurally decoupled from the sub-second electromechanical dynamics of the grid. When a severe three-phase short circuit occurs, these algorithms cannot dynamically adjust the virtual rotor angle or the instantaneous converter references, preventing extension of the system CCT.

### 6.4. Knowledge Gap and Research Contribution

A clear gap exists in the contemporary literature: the lack of a unified control framework that structurally connects the transient electromechanical objectives of the grid (nadir, RoCoF, CCT) with the electrochemical operating constraints (SoC, Vp) in real time. The proposed framework addresses this gap by implementing a successive-linearization MPC (SL-MPC) scheme.

By introducing an asymmetric nonlinear energy envelope directly into the constraint matrix of the predictive optimizer, the control actions (J*(*k*), D*(*k*)) are dynamically adapted according to the real-time BESS capability. This ensures maximum transient-stability improvement when the BESS is in good condition while providing autonomous and safe parameter limitation when the BESS approaches its lower operating limits.

Table 33 compares studies related to microgrid control for transient-stability studies.

### 6.5. Practical Implementation and Hardware-in-the-Loop Considerations

Although the proposed MPC–VSG–BESS framework has been evaluated through detailed EMT simulations, this study did not aim to provide experimental validation or real-time hardware validation. The numerical validation aimed to assess the dynamic feasibility of the proposed control architecture under severe contingencies, including three-phase faults, disconnections of conventional generation, and high-renewable-energy penetration conditions. The results demonstrate that the proposed controller can coordinate virtual inertia, virtual damping, and active-power support from the BESS while simultaneously meeting the modeled electrical and energy constraints.

From an implementation perspective, the proposed architecture is compatible with a hierarchical real-time control platform. The local AEA-VSG layer operates at a faster sampling frequency than the MPC supervisor. The latter solves a finite-horizon quadratic optimization problem with a 10 ms sampling interval. The reported average and maximum MPC execution times—approximately 2.8 ms and 6.5 ms, respectively—remain below the 10 ms control interval, indicating that the optimization problem is computationally compatible with the chosen control rate under the simulated conditions.

However, real-time implementation introduces additional effects that cannot be fully represented by the current offline simulation framework. These effects include analog–digital conversion, sensor noise, signal quantization, communication delays, computational jitter, PWM synchronization, controller execution latency, and finite-resolution measurements of current, voltage, and BESS state of charge (SoC). Such effects can alter the effective phase margin and the transient response of the adaptive VSG, and therefore must be explicitly evaluated before deployment in a physical system.

An appropriate subsequent validation step would be a controller-hardware-in-the-loop (CHIL) or power-hardware-in-the-loop (PHIL) implementation, where the detailed microgrid plant runs in real time using an RTDS or OPAL-RT platform, while the proposed MPC–VSG controller is deployed on a real-time control processor or digital signal processor. This setup would allow testing the control law under realistic sampling, communication, detection, and execution constraints without initially exposing the experimental BESS converter to severe fault conditions.

The HIL framework also enables systematic evaluation of the controller against communication delays, sensor noise, parameter uncertainties, converter saturation, and recurrent disturbances. In a later PHIL stage, the controller could be coupled to a scaled BESS converter or power electronics interface to experimentally verify active-power response, adaptive trajectories of *J* and *D*, constraint adaptation based on SoC, and frequency-support capacity during transients.

Consequently, HIL/PHIL validation and experimental testing are considered an important extension of this work, rather than a prerequisite for the numerical proof of concept presented here. This limitation is explicitly acknowledged, and real-time validation using RTDS or OPAL-RT is identified as a priority for future research.

## 7. Conclusions and Future Work

### 7.1. Conclusions

The finite-horizon, energy-aware MPC framework successfully coordinates the adaptive inertia of the VSG, virtual damping, and active-power support through BESS to improve transient stability in hybrid microgrids dominated by renewable sources. The results demonstrate that the benefit of the proposed strategy stems from the predictive coordination of virtual inertia with the energy availability of the BESS, rather than from simply adaptively tuning the *J* and *D* parameters.

In the most critical 100% RES scenario, the conventional control setup shows a minimum frequency nadir value of 56.78 Hz, while the proposed MPC–VSG–BESS scheme maintains this value at 59.26 Hz. This represents a 77.0% reduction in the magnitude of the nadir deviation. The strategy also increases the CCT from 0 ms to 185 ms under the severe fault condition considered. During the transient dynamic analisys, the maximum observed virtual inertia and damping values are 48.5 kg·m^2^ and 38.2 N·m·s/rad, respectively. These results demonstrate the ability of the proposed scheme to provide synthetic inertia support and damping when physical synchronous inertia is unavailable.

Furthermore, the energy-sensitive mechanism prevents the indiscriminate deployment of synthetic inertia when the BESS’s energy availability is limited. In the case of low-SoC sensitivity—with an initial SoC of 22%—the permissible virtual inertia limit is reduced to 12.4 kg·m^2^. This result confirms that the proposed controller links transient-stability support to the available energy resource, rather than treating the BESS as an unrestricted power source.

A limitation of this study is that the proposed controller has only been validated through detailed EMT simulations and has not yet been tested using real-time HIL systems or physical power hardware. Consequently, effects related to implementation, such as sensor noise, communication delays, computational fluctuations, and converter hardware limitations, have not been experimentally quantified. However, the computational results indicate that the MPC runtime remains below the selected monitoring sampling interval of 10 ms, laying the groundwork for future real-time implementation. Therefore, CHIL/PHIL validation using RTDS or OPAL-RT is identified as the next experimental step for the proposed framework.

### 7.2. Key Research Findings

The comprehensive simulations performed in MATLAB–Simulink^®^ using a gradual RES-penetration matrix (from 10% to 100%) yielded the following definitive scientific conclusions.

Superior frequency containment with zero inertia: In the worst case, with disconnection of 20% of the generation capacity and 100% RES penetration (with no physical spinning reserves), the proposed controller arrested the frequency drop at a safe minimum of 59.26 Hz and limited RoCoF to −0.32 Hz/s. By contrast, conventional droop controls failed completely, causing a catastrophic frequency drop below the underfrequency load-shedding (UFLS) threshold (57.50 Hz) to 56.78 Hz, which caused localized blackouts in the hybrid microgrid.

Sub-second parameter-decoupling optimization: Real-time optimization tracking confirmed that the controller effectively decouples the control-action variables. After detecting a contingency, the system drives *J*(*t*) to its maximum limits (48.5 kg·m^2^) to absorb the initial RoCoF burst, subsequently damping the post-fault power oscillations through a sharp increase in *D*(*t*) up to 38.2 N·m·s/rad, reducing the system stabilization time to less than 1.82 s.

CCT extension: During a severe symmetric three-phase-to-ground short-circuit fault with 100% RESs, the proposed system maintained the stiffness of the grid-forming voltage vector, extending the absolute CCT from instantaneous collapse of 0 ms (with traditional voltage sag) to a robust 185 ms (and up to 245 ms with 50% RESs). This extension provides a vital safety margin for physical overcurrent protection coordination devices to clear severe faults without causing widespread converter disconnections.

Bidirectional safety nexus verification: When evaluated under a depleted-energy limit state (SoC_init_ = 22%), the energy-aware prefilter successfully adapted the optimization limits, restricting the maximum inertia to 12.4 kg·m^2^. This parameter limitation protected the BESS assembly from excessive chemical-diffusion stress and polarization-voltage collapse, demonstrating the bidirectional reliability of the multilayer design.

### 7.3. Future Work

Although the proposed framework has demonstrated high effectiveness and mathematical convergence in a simulation environment, future research will focus on the following milestones.

The next stage of the research will implement the proposed MPC–VSG–BESS controller in a CHIL environment using an RTDS or OPAL-RT real-time digital simulator coupled to a real-time control processor/DSP. This stage will evaluate the effects of communication delays, sensor noise, computational latency, sampling synchronization, and parameter uncertainty on the proposed adaptive control strategy. Subsequently, a PHIL configuration involving a scaled BESS converter will be considered to experimentally verify active-power support, SoC-dependent constraint adaptation, and transient frequency response. This progression will provide an experimental bridge between the EMT simulation results reported in this work and future laboratory-scale validation.

Multi-asset coordination architecture: Extension of the predictive model to integrate the dynamic transient capabilities of hydrogen fuel cells and distributed supercapacitors, establishing a multilevel energy-aware hybrid storage layer.

Machine-learning-enhanced linearization: Use of physics-informed neural networks (PINNs) to perform online calculations of the linearization matrix, further reducing the computational burden of the QP solver in highly unbalanced hybrid-microgrid topologies.

## Figures and Tables

**Table 1:** Critical comparison of existing control paradigms for low-inertia renewable-dominated microgrids.

Control Paradigm	Adaptive *J*, *D*	Predictive Optimization	Explicit BESS Energy/SoC Constrains	Transient-Stability Objective	Computational Burden	Real-Time Feasibility	Scalability	Experimental/HIL Validation
Fixed-parameter VSG	No	No	No	Limited	Low	High	High	Relatively mature
Heuristic/adaptive VSG	Yes	No	Usually limited	Yes	Low–moderate	High	Moderate–high	Variable
BESS-only frequency support	No	No	Yes	Partial	Low	High	High	Relatively mature
Conventional MPC	Potentially	Yes	Often partial	Yes	Moderate–high	Conditional	Moderate	Increasing
MPC-based VSG	Yes	Yes	Partial/variable	Yes	Moderate–high	Conditional	Moderate	Limited
VSG–BESS coordinated control	Yes	Usually no	Yes	Yes	Moderate	High	Moderate	Limited
**Proposed** **MPC–VSG–BESS**	**Yes**	**Yes**	**Yes**	**Explicitly** **included**	**Moderate**	Yes *	**Moderate**	**Simulation-based**

* The proposed controller demonstrates computational feasibility in the present MATLAB–Simulink implementation, with an average computation time of 2.8 ms and a maximum below 6.5 ms for a 10 ms control period.

**Table 2:** Positioning of the proposed framework with respect to existing adaptive VSG and MPC-based approaches.

Approach	Adaptive *J*, *D*	Finite-Horizon Prediction	SoC-Dependent Feasible *J* Region	Coordinated BESS Power	Future Energy Availability	High RES/Near-100% Scenario	Transient CCT Assessment
Fixed VSG	×	×	×	×	×	Limited	Limited
Heuristic adaptive VSG	✓	×	×/limited	Partial	×	Partial	Variable
BESS frequency support	×	×	×	✓	Partial	✓	Variable
Conventional MPC	Partial	✓	Partial	✓	Partial	✓	✓
Adaptive VSG–MPC	✓	✓	Not necessarily explicit	✓	Not necessarily explicit	✓	✓
**Proposed** **MPC–VSG–BESS**	**✓**	**✓**	**✓**	**✓**	**✓**	**✓ 70–100%**	✓

**Table 3:** Technical specifications of the proposed hybrid microgrid.

Component	Technology/Model	Rated Capacity	Voltage Level	Main Characteristics	Operational Function
PV system	Utility-scale monocrystalline PV array with two-stage grid-connected VSI	2 MW	0.48/13.8 kV	DC-link architecture with MPPT control; average efficiency: 97%; inverter switching frequency: 10 kHz	Renewable active-power generation and grid support
Wind turbine	Variable-speed type III and IV wind turbine with full-scale B2B converter	4 MW	0.69/13.8 kV	DFIG and PMSG; vector control strategy; pitch angle regulation	Renewable power generation and frequency support
Diesel generator	Medium-speed synchronous diesel generator	1 MW	13.8 kV	4-pole synchronous machine; governor and excitation system included; inertia constant (H = 4.5 s)	Dispatchable generation and inertial support
Combined-cycle generation unit (CCGT)	Gas turbine combined-cycle synchronous generator	2 MW	13.8 kV	High-efficiency thermal generation unit; IEEE type 1 excitation system; inertia constant (H = 5.5 s)	Base-load support and transient stabilization
BESS	Lithium-ion BESS with bidirectional voltage-source converter	1 MW/2 MWh	1 kV DC–13.8 kV AC	SoC operating range: 20–90%; response time < 50 ms; MPC-based EMS	Fast frequency response and transient support
Adaptive VSG controller	Grid-forming inverter-based VSG architecture	--	--	Adaptive inertia and damping emulation based on RoCoF, frequency deviation, and SoC	Virtual inertia and transient frequency stabilization
	Integrated in BESS converter				
Utility grid interconnection	Infinite bus equivalent model	External grid	69/13.8 kV	Grid-connected operation through transformer coupling	Frequency reference and power exchange
Local load demand	Aggregated dynamic ZIP load model	10.5 MW	13.8 kV	Combination of industrial, commercial, and residential demand characteristics	System loading condition

**Table 4:** Electrical infrastructure parameters.

Element	Parameter	Value
PCC voltage level	$V_{PCC}$	13.8 kV
Utility grid voltage	$V_{grid}$	69 kV
Nominal frequency	$f_{0}$	60 Hz
Transformer impedance	$Z_{T}$	6% p.u.
Distribution feeder R/X ratio	R/X	0.35
Average feeder length	$L_{f}$	2.5 km
Short-circuit ratio	${\mathrm{Ratio}}_{\mathrm{sc}}$	3.2
Base power	$S_{base}$	100 MVA

**Table 5:** Synchronous condenser vs. synthetic inertia.

Characteristic	Synchronous Condenser	Converter-Based Synthetic Inertia	Preferred Application
Physical inertia	Yes; genuine rotating kinetic energy	No physical rotating inertia; emulated dynamically	Synthetic inertia: low-inertia systems requiring fast controllability
Response speed	Fast electromechanical response; limited by machine/excitation dynamics	Very fast; limited mainly by converter/control bandwidth	Synthetic inertia for sub-second transient support
Short-circuit contribution	High relative contribution to system short-circuit strength	Limited; converter current is constrained	Synchronous condenser where fault strength is critical
Voltage support	Strong reactive-power/voltage support through excitation	Can provide converter-based voltage support, but limited by converter ratings	Synchronous condenser for grid-strength/voltage reinforcement
Energy source	Mechanical rotating energy; no BESS energy depletion	Requires available DC-side/BESS energy for sustained active-power support	Synthetic inertia when energy-aware scheduling is required
Controllability	Primarily excitation- and operating state-dependent	Directly programmable through converter control	Synthetic inertia for adaptive, predictive operation
Main limitation	Rotating machinery, cost, footprint and maintenance	Power/energy/current limits and no restoration of physical fault current	Technology selected according to the required service

**Table 6:** Measurement variables and their functions in the proposed AEA-VSG–MPC.

Measurement/ Estimated Variable	Source/Interface	Function in the Proposed Framework	Control Layer
$V_{\mathrm{PCC}}$	PCC voltage measurement	Electrical operating-condition monitoring	Measurement/ local control
$I_{\mathrm{PCC}}$	PCC current measurement	Current and power-flow monitoring	Measurement
$P_{\mathrm{PCC}}$	Derived from $V_{\mathrm{PCC}}$ and $I_{\mathrm{PCC}}$	Active-power imbalance assessment	AEA-VSG–MPC
$Q_{\mathrm{PCC}}$	Derived from $V_{\mathrm{PCC}}$ and $I_{\mathrm{PCC}}$	Reactive-power operating-condition assessment	Local control
*f*	Frequency estimation from electrical measurement	Frequency-deviation assessment	AEA-VSG–MPC
RoCoF	Derived from *f*	Disturbance severity and adaptive-support indicator	AEA-VSG–MPC
$P_{\mathrm{BESS}}$	BESS converter	Feedback of active-power support	AEA-VSG–MPC
SoC	BESS state estimation	Energy availability and MPC constraint	MPC

**Table 7:** Initial conditions of the microgrid.

Parameter	Value
Frequency	60 Hz
PCC voltage	1.0 p.u.
Initial SoC	80%
Load demand	10.5 MW
Reactive power demand	4.47 VAr

**Table 8:** Electrical and operational parameters of the PV systems.

Parameter	Symbol	Value	Unit
Rated power	$P_{\mathrm{PV},\;\mathrm{rated}}$	2	MW
Rated apparent power	$S_{\mathrm{PV}}$	3.3.	MVA
Nominal AC voltage	$V_{AC}$	13.8	kV
DC-link voltage	$V_{\mathrm{DC}}$	1000	V
Nominal frequency	$f_{0}$	60	Hz
Converter topology	--	Two-stage VSI	--
PV technology	--	Monocrystalline silicon	--
MPPT algorithm	--	Perturb and observe (P&O)	--
Inverter efficiency	$n_{\mathrm{inv}}$	97.5	%
DC–DC converter efficiency	$n_{\mathrm{dc}}$	98	%
Switching frequency	$f_{\mathrm{sw}}$	10	kHz
Power factor	PF	1.0	p.u.
Irradiance at STC	$G_{\mathrm{STC}}$	1000	W/m^2^
Cell temperature at STC	$T_{\mathrm{STC}}$	25	°C
Maximum power voltage	$V_{\mathrm{MPP}}$	41.2	V
Open-circuit voltage	$V_{\mathrm{oc}}$	49.8	V
Short-circuit current	$I_{\mathrm{sc}}$	13.9	A
Temperature coefficient of power	$K_{P}$	−0.35	%/°C
Series resistance	$R_{s}$	0.28	Ω
Shunt resistance	$R_{\mathrm{sh}}$	450	Ω
Diode ideality factor	*a*	1.3	--

**Table 9:** PV module parameters.

Parameter	Value
Module technology	Monocrystalline PERC
Rated module power	540 W
Module efficiency	21.1%
Cells per module	98
Open-circuit voltage	49.8 V
Short-circuit current	13.9 A
Maximum power voltage	41.2 V
Maximum power current	13.1 A

**Table 10:** Dynamic parameters of the PV converter.

Parameter	Symbol	Value
Current control bandwidth	${\mathrm{BW}}_{i}$	350 Hz
Voltage control bandwidth	${\mathrm{BW}}_{v}$	67 Hz
PLL bandwidth	${\mathrm{BW}}_{\mathrm{PLL}}$	8 Hz
DC-link capacitance	$C_{\mathrm{dc}}$	17.5 mF
Filter inductance	$L_{f}$	1.8 mH
Filter resistance	$R_{f}$	0.01 Ω
Converter response time	$T_{c}$	5 ms
Reactive power capability	$Q_{\max}$	±1.5 MVAr

**Table 11:** Technical parameters of the DFIG wind-turbine generator.

Parameter	Symbol	Value	Unit
Rated power	$P_{\mathrm{DFIG}}$	2	MW
Rated apparent power	$S_{\mathrm{DFIG}}$	2.2	MVA
Rated voltage	$V_{\mathrm{LL}}$	690	V
Grid connection voltage	$V_{\mathrm{grid}}$	13.8	kV
Rated frequency	$f_{0}$	60	Hz
Number of poles	p	4	--
Rated rotor speed	$\omega_{r}$	1.2	p.u.
Cut-in wind speed	$V_{\mathrm{cut}\text{-}\mathrm{in}}$	4	m/s
Rated wind speed	$V_{\mathrm{rated}}$	12	m/s
Cut-out wind speed	$V_{\mathrm{cut}\text{-}\mathrm{out}}$	25	m/s
Rotor radius	*R*	45	m
Swept area	*A*	6362	m^2^
Air density	ρ	1.225	kg/m^3^
Maximum power coefficient	$C_{p\text{\_}\max}$	0.48	--
Optimal tip-speed ratio	${⋋}_{\mathrm{opt}}$	8.1	--
Gearbox ratio	$G_{r}$	90	--
Generator inertia constant	$H_{\mathrm{DFIG}}$	3.5	s
Stator resistance	$R_{s}$	0.00706	p.u.
Rotor resistance	$R_{r}$	0.005	p.u.
Stator leakage reactance	$X_{s}$	0.171	p.u.
Rotor leakage reactance	$X_{r}$	0.156	p.u.
Magnetizing reactance	$X_{m}$	2.9	p.u.
Converter rating	$S_{\mathrm{conv}}$	0.3 $S_{\mathrm{rated}}$	p.u.
Rotor-side converter frequency	$f_{\mathrm{sw}}$	2.5	kHz
Grid-side converter frequency	$f_{\mathrm{sw}}$	2.5	kHz

**Table 12:** Technical parameters of the PMSG wind-turbine generator.

Parameter	Symbol	Value	Unit
Rated power	$P_{\mathrm{PMSG}}$	2	MW
Rated apparent power	$S_{\mathrm{PMSG}}$	2.2	MVA
Rated voltage	$V_{\mathrm{LL}}$	690	V
Grid connection voltage	$V_{\mathrm{grid}}$	13.8	kV
Rated frequency	$f_{0}$	60	Hz
Number of poles	p	88	--
Cut-in wind speed	$V_{\mathrm{cut}\text{-}\mathrm{in}}$	3.5	m/s
Rated wind speed	$V_{\mathrm{rated}}$	11.5	m/s
Cut-out wind speed	$V_{\mathrm{cut}\text{-}\mathrm{out}}$	25	m/s
Rotor radius	*R*	46	m
Swept area	*A*	6648	m^2^
Air density	ρ	1.225	kg/m^3^
Maximum power coefficient	$C_{p\text{\_}\max}$	0.49	--
Optimal tip-speed ratio	${⋋}_{\mathrm{opt}}$	8.3	--
Permanent magnet flux linkage	$\psi_{f}$	1.18	Wb
d-axis inductance	$L_{d}$	4.2	mH
q-axis inductance	$L_{q}$	4.0	mH
Machine inertia constant	$H_{\mathrm{PMSG}}$	4.0	s
DC-link voltage	$V_{\mathrm{dc}}$	1200	V
Converter switching frequency	$f_{\mathrm{sw}}$	2.5	kHz
Full-scale converter rating	$S_{\mathrm{conv}}$	100%	p.u.

**Table 13:** Dynamic control parameters of wind-turbine converters.

Parameter	DFIG	PMSG
Active power control bandwidth	40 Hz	50 Hz
Reactive power control bandwidth	30 Hz	40 Hz
Current control bandwidth	400 Hz	500 Hz
PLL bandwidth	20 Hz	20 Hz
DC-link capacitance	20 mF	25 mF
Converter response time	8 ms	5 ms
Reactive power capability	±0.6 p.u.	±1.0 p.u.
LVRT threshold	0.15 p.u.	0.15 p.u.

**Table 14:** Comparison of dynamic characteristics—DFIG and PMSG.

Characteristics	DFIG	PMSG
Grid coupling	Partial	Fully decoupled
Converter size	25–30% rated power	100% rated power
Physical inertia visibility	Medium	Very low
Frequency-support capability	Limited	Very limited
Fault current contribution	Moderate	Low
Transient-stability contribution	Moderate	Low
Synthetic inertia requirement	Medium	High
Suitability for VSG integration	Good	Excellent

**Table 15:** BESS equivalent circuit parameters.

Parameter	Symbol	Value
Rated power	$P_{\mathrm{rated}\text{\_}\mathrm{BESS}}$	1 MW
Rated energy	$E_{\mathrm{rated}}$	2 MWh
Nominal voltage	$V_{\mathrm{nom}\text{\_}\mathrm{BESS}}$	1000 V
Internal resistance	$R_{0}$	0.015 Ω
Polarization resistance	$R_{p}$	0.008 Ω
Polarization capacitance	$C_{p}$	18 F
Round-trip efficiency	$\eta_{\mathrm{rt}}$	95%

**Table 16:** Parameter specifications for the 1 MW diesel generator.

Parameter	Symbol	Value	Unit	Description/Physical Relevance
Nominal power	$P_{n}^{d}$	1.0	MW	Rated active-power capacity of the unit.
Nominal voltage	$V_{n}^{d}$	4.16	kV	Line-to-line stator terminal voltage.
Inertia constant	$H_{d}$	4.5	s	Mechanical rotor mass inertia constant.
Damping coefficient	$D_{d}$	1.2	p.u.	Friction and windage damping effect.
Governor speed droop	$R_{d}$	4.0	%	Steady-state frequency regulation gain.
Governor time constant	$T_{g}$	0.05	s	Speed governor hydraulic/electronic response lag.
Combustion delay	$T_{d}$	0.12	s	Diesel engine fuel injection and combustion transp.
Stator resistance	$R_{s}$	0.003	p.u.	Internal armature winding resistance.
Synchronous reactance	$X_{d}$	1.56	p.u.	Direct-axis steady-state reactance.
Transient reactance	${X\prime }_{d}$	0.28	p.u.	Direct-axis transient reactance during faults.

**Table 17:** Parameter specifications for the 2 MW combined–cycle power plant.

Parameter	Symbol	Value	Unit	Description/Physical Relevance
Nominal power base	$P_{m}^{\mathrm{cc}}$	2.0	MW	Total aggregated rating (GT + ST combined).
Gas turbine fraction	$X_{\mathrm{GT}}$	65	%	Proportional power shared by the GT stage.
Steam turbine fraction	$X_{\mathrm{ST}}$	35	%	Proportional power shared by the ST stage.
Aggregated inertia	$H_{\mathrm{cc}}$	4.8	s	Combined mechanical inertia of multi-shaft rotors.
Structural damping	$D_{\mathrm{cc}}$	1.5	p.u.	Internal mechanical damping under dynamic oscillations.
Permanent droop	$R_{\mathrm{cc}}$	5.0	%	Speed control regulation characteristic.
Fuel valve lag	$T_{f}$	0.1	s	Positioning time constant of the main gas fuel valve.
Compressor delay	$T_{c}$	0.23	s	Air compressor discharge and volume charging lag.
HRSG transport lag	$T_{h}$	2.5	s	Thermal exhaust transport delay into steam generation.
HRSG efficiency gain	$T_{\mathrm{HRSG}}$	0.92	--	Heat recovery energy conversion efficiency multiplier.
Steam chest constant	$T_{\mathrm{st}}$	5.5	s	Thermodynamic charging delay of the steam turbine chest.

**Table 18:** Load model parameters.

Parameter	Symbol	Value	Unit	Description/Physical Relevance
Nominal active power	$P_{0}$	10.5	MW	Aggregated μG active-power demand base.
Nominal reactive power	$Q_{0}$	4.47	MVAr	Aggregated μG reactive power demand base.
Nominal bus voltage	$V_{0}$	4.16	kV	Line-to-line RMS base voltage at the primary distribution load bus.
Nominal system frequency	$f_{0}$	60	Hz	Fundamental nominal operating frequency of the grid.
Constant impedance fraction (*P*)	$a_{1}$	0.40	--	Proportional fraction of active load behaving as pure impedance (e.g., resistive heating, non-regulated thermal loads).
Constant current fraction (*P*)	$a_{2}$	0.30	--	Proportional fraction of active load behaving as pure current (e.g., resistive heating, non-regulated thermal loads).
Constant power fraction (*P*)	$a_{3}$	0.30	--	Proportional fraction of active load behaving as pure power (e.g., resistive heating, non-regulated thermal loads).
Constant impedance fraction (*Q*)	$a_{4}$	0.30	--	Proportional fraction of reactive load behaving as pure impedance (e.g., resistive heating, non-regulated thermal loads).
Constant current fraction (*Q*)	$a_{5}$	0.30	--	Proportional fraction of reactive load behaving as pure current (e.g., resistive heating, non-regulated thermal loads).
Constant power fraction (*Q*)	$a_{6}$	0.40	--	Proportional fraction of reactive load behaving as pure power (e.g., resistive heating, non-regulated thermal loads).
Active frequency sensitivity	$K_{\mathrm{pf}}$	1.20	%/Hz	Active power sensitivity multiplier; dictates how much the active power drops as system frequency drops.
Reactive frequency sensitivity	$K_{\mathrm{qf}}$	−1.30	%/Hz	Reactive power sensitivity multiplier; dictates how much the active power drops as system frequency drops.

**Table 19:** Operational constraints of the BESS.

Constraint	Symbol	Value
Rated power	$P_{\mathrm{BESS}\text{\_}\max}$	1 MW
Rated energy	$E_{\mathrm{BESS}\text{\_}\max}$	2 MWh
Minimum SoC	${\mathrm{SoC}}_{\min}$	20%
Maximum SoC	${\mathrm{SoC}}_{\max}$	90%
Minimum voltage	$V_{\min}$	0.90 p.u.
Maximum voltage	$V_{\max}$	1.10 p.u.
Maximum current	$I_{\max}$	1.2 p.u.
Maximum ramp rate	${\mathrm{RR}}_{\max}$	0.5 MW/s
Minimum inertia adaptation gain	$G_{J\text{\_}\min}$	1
Maximum inertia adaptation gain	$G_{J\text{\_}\max}$	10
Minimum damping adaptation gain	$G_{D\text{\_}\min}$	0.1
Maximum damping adaptation gain	$G_{D\text{\_}\max}$	5

**Table 20:** MPC configuration and computational performance.

Parameter	Value
Optimization type	Quadratic programming (QP)
Solver	Active-set QP
Prediction horizon $\left(N_{p}\right)$	20
Control horizon $\left(N_{c}\right)$	10
Sampling time $\left(T_{s}\right)$	10 ms
States	4
Control inputs	3
Constraints	12
Average computation time	2.8 ms
Maximum computation time	6.5 ms
Real-time feasibility	Yes
Constrain handling	Soft constraints
Feasibility mechanism	Slack variables
Constraint smoothing	Progressive limit activation

**Table 21:** Expected impact on transient stability.

Performance Indicator	Expected Improvement
Critical Clearing Time (CCT)	Increase
Maximum RoCoF	Reduction
Frequency Nadir	Improvement
Settling Time	Reduction
Frequency Overshoot	Reduction
Oscillation Damping	Improvement
BESS Utilization Efficiency	Improvement
Renewable Hosting Capacity	Increase

**Table 22:** Verified simulation and control configuration.

Parameter	Value
Simulation environment	MATLAB–Simulink^®^ + Simscape Electrical^TM^
Simulation domain	EMT, Time–domain
Numerical solver	Fixed–step discrete
Electrical integration step	50 μs
Total simulation duration	60 *s*
Fault inception time	20 *s*
Nominal system frequency	60 Hz
PCC voltage	13.8 kV
System base power	100 MVA
AEA-VSG sampling time	1 ms
MPC sampling time	10 ms
MPC prediction horizon	$N_{p}$ = 20
MPC control horizon	$N_{c}$ = 10
MPC prediction window	0.2 s
MPC control-action window	0.1 s
BESS rated power	1 MW
BESS rated energy	2 MWh

**Table 23:** Definition of the renewable-penetration and synchronous-generation scenarios.

Penetration Level	Diesel Unit (1 MW Base)	Combined-Cycle (2 MW base)	RESs	Total Synchronous Generation	Physical Inertia $\left(H_{\mathrm{sys}}\right)$
0%	0	0	0	0	4.27
10%	1.00 MW (100%)	2.00 MW (100%)	1.05 MW	3.0 MW	High $\left(H_{d}+H_{\mathrm{cc}}\right)$ = 4.01
20%	1.00 MW (100%)	2.00 MW (100%)	2.10 MW	3.0 MW	High $\left(H_{d}+H_{\mathrm{cc}}\right)$ = 3.55
30%	0.85 MW (85%)	1.80 MW (90%)	3.15 MW	2.65 MW	Moderate (governor) = 3
50%	0.50 MW (50%)	1.25 MW (62.5%)	5.25 MW	1.75 MW	Low (governed) = 2.5
70%	0.20 MW (20%)	0.75 MW (37.5%)	7.35 MW	0.95 MW	Critical (minimum) = 1.5
90%	0.00 MW–Isolated	0.25 MW (12.5%)	9.45 MW	0.25 MW	Ultra-Low $\left(H_{\mathrm{cc}}\right)$ = 1.25
100%	0.00 MW–Isolated	0.00 MW–Isolated	10.50 MW	0.00 MW	Zero physical inertia

Note: RES penetration is defined based on the total active-power contribution from PV units, DFIG, and PMSG. The BESS is not included in the RES penetration metric, as it is an energy-storage resource and not a primary generation source. The reported RES values represent the renewable contribution in steady-state conditions that define the scenario. The internal distribution among PV, DFIG, and PMSG units is not detailed separately, as it is not necessary to define the penetration metric and was not uniformly documented for all operating points. Diesel and CCGT units represent the contribution of synchronous generation and consequently determine the availability of physical electromechanical inertia.

**Table 24:** Indicators evaluating the effect of RESs on overall transient stability in the event of a three-phase short circuit.

	Case Study		
	1	2	3
	RES = 0%	RES = 10%	RES = 30%
$H_{\mathrm{sys}}$ $\left(s\right)$	4.27	4.01	2.89
*RoCoF* $\left(\mathrm{Hz}/s\right)$	1.23	1.38	Unstable
*Nadir Frequency*	59.74	59.12	Unstable
CCT (ms)	270	180	90
$I_{SC\_N1}$ (kA)	7.43	6.78	6.03

**Table 25:** Quantitative degradation of transient-stability indicators with increasing renewable penetration.

RES Penetration	$H_{\mathrm{sys}}$ $\left(s\right)$	RoCoF $\left(\mathrm{Hz}/s\right)$	Frequency Nadir (Hz)	CCT (ms)	Stability Condition
0%	4.27	1.23	59.74	270	Stable
10%	4.01	1.06	59.49	180	Stable
30%	2.89	0.97	59.46	90	Reduced stability margin
50%	2.60	0.88	59.45	10	Critical
70%	1.25	Unstable	Unstable	Unstable	Unstable

**Table 26:** Optimal MPC parameters for case study 6.

Gen.	Optimized MPC				
	K	$T_{1}$	$T_{2}$	$T_{3}$	$T_{4}$
N1	31.61	0.06	2.24	1.17	1.62
N12	15.59	2.34	1.20	0.9	0.36
N15	25.53	0.02	0.65	2.46	0.22

**Table 27:** Optimal BESS controller parameters for case study 6.

$T_{r}$	$T_{\mathrm{rq}}$	$K_{p}$	$T_{\mathrm{ip}}$	$K_{q}$	$T_{\mathrm{iq}}$
1.89	0.59	7.57	0.36	13.19	1.57

**Table 28:** Normalized inertia factors associated with the MPC configuration under different renewable-penetration scenarios.

		Value		
Name	Description	RES = 70%	RES = 90%	RES = 100%
NG	Number of generators	9	8	7
ND	Number of data	22	36	55
NP_MPC	MPC parameters	2	5	8
NP_BESS	BESS parameters	6	8	11
$\delta_{0}$	Real-part eigenvalue threshold	−2	−5	−8
$\xi_{0}$	Damping threshold	5%	7%	10%
α	Scaling factor	10	12	15
$J_{n\text{\_}máx}$	Scenario-specific MPC upper bound for $J_{n}$	0.7	0.73	0.78
$J_{n\text{\_}\min}$	Scenario-specific MPC lower bound for $J_{n}$	0.01	0.02	0.04
$C_{1\text{\_}máx}$	Upper damping-related parameter	0.8	1.2	1.5
$C_{1\text{\_}\min}$	Lower damping-related parameter	0.2	0.3	0.5
$C_{2}$	Parameters of $C_{2}$	0.3	0.6	0.7

Note: The l normalized J_n_ values in this table represent MPC configuration limits specific to each scenario. They are not SoC values and should not be interpreted as direct evaluations of the SoC-dependent, energy-aware function defined in (18). During closed-loop operation, the optimal value J_n_^*^(k) is selected within the feasible region defined by the active MPC constraints.

**Table 29:** Optimal MPC parameters for case study 7.

SG	Optimized MPC				
	K	$T_{1}$	$T_{2}$	$T_{3}$	$T_{4}$
N12	31.61	0.06	2.24	1.17	1.62

**Table 30:** Optimal BESS controller parameters for case study 7.

$T_{r}$	$T_{\mathrm{rq}}$	$K_{p}$	$T_{\mathrm{ip}}$	$K_{q}$	$T_{\mathrm{iq}}$
1.32	0.63	7.02	0.22	12.75	1.33

**Table 31:** Summary of the MPC–VSG–BESS response under high renewable penetration.

Scenario	Res Penetration	Synchronous Generation	Controller	Main Observed Response
Case study 6	70%	N1, N12, N15	MPC	Reduced transient duration
Case study 6	70%	N1, N12, N15	MPC + BESS	Further transient suppression
Case study 7	90%	N12	MPC	Improved frequency/ voltage response
Case study 7	90%	N12	MPC + BESS	Stable frequency support during generator outage
Case study 8	100%	None	MPC + VSG + BESS	BESS establishes voltage- frequency reference

**Table 32:** Quantitative performance comparison of the proposed MPC–VSG–BESS framework.

RES Penetration	Control Configuration	$f_{\max}$ (Hz)	$f_{\mathrm{nadir}}$ (Hz)	Maximum Frequency Deviation	CCT (ms)	Transient/Setting Time
70%	Conventional	60.78	59.88	0.78 Hz	NR	>4 s
70%	MPC	60.73	59.90	0.73 Hz	NR	≈1 s
70%	MPC + BESS	60.27	59.90	0.27 Hz	NR	≈1 s
90%	Conventional	60.92	58.33	1.67 Hz	NR	NR
90%	MPC	60.44	59.76	0.44 Hz	NR	NR
90%	MPC + BESS	60.32	59.26	0.74 Hz	NR	NR
100%	Conventional	NR	56.78	3.22 Hz	0	NR
100%	MPC + VSG + BESS	NR	59.26	0.74 Hz	185	<1.82 s

NR: not reported or not recoverable from the available simulation record.

**Table 33:** Comprehensive comparative matrix of state-of-the-art transient-stability controls.

Control Framework and Reference	Control Layer	Core Optimization Algorithm	BESS SoC/Energy Awareness	Multi-Rate Execution	Real-Time FRT and CCT Extension	Primary Technical Vulnerability/ Research Gap
Heuristic & Alternating VSG Schemes (e.g., Alipoor et al.; Fang et al., 2022) [42]	Local	Fuzzy-logic/step-by-step parameter alternation rules	No (assumes infinite DC source)	No	Poor (causes post-fault parameter hunting and windup)	Lacks mathematical optimality; high risk of underdamped power oscillations during short-circuits
Robust $H_{\infty }$/Droop Control (e.g., Oureilidis et al., 2023) [43]	Local	Worst-case frequency disturbance attenuation	No	No	Moderate (bounded voltage stiffness)	Fixed conservative safety margins reduce system flexibility; incapable of handling high-penetration RES scaling dynamically
Nonlinear MPC for VSG Loops (e.g., Elwakil et al., 2023) [44]	Supervisory	Nonlinear interior-point/SQP optimization	Partial (static power tracking limits)	No	Moderate (lacks overcurrent loop synchronization)	Excessive computational burden; solver execution times exceed the fast-sampling constraints required for primary control
Degradation-Aware Microgrid Dispatch (e.g., Zhang & Control Cohort, 2025) [45]	Tertiary	Mixed-integer programming (MILP)	High (tracks battery lifetime degradation and capacity fade)	Yes (minutes/ hours/scale)	None (static baseline dispatch references only)	Incapable of reacting to sub-second transient contingencies; cannot prevent UFLS activation during generation trips
This paper	Dual-layer (supervisory + local)	Successive linearization quadratic programming (SL-QP)	High (dynamic asymmetric exponential boundary tracking)	Yes (50 μs/1 ms/10 ms multi-rate loop synchronization)	Excellent (guarantees safe maximum CCT extension up to 245 ms)	Requires accurate online tracking of BESS state variables (Soc and $V_{p}$) to maintain precise constraint mapping

## Data Availability

We are fully committed to providing any additional data, simulation files, or supplementary information required. Please kindly let us know the preferred guidelines, platform, or repository where we should upload or submit this information by email: jdrr@ier.inam.mx.

## Abbreviations

The following abbreviations are used in this manuscript.

RES	renewable energy sources
RoCoF	rate of change of frequency
MG	microgrid
VSG	virtual synchronous generator
BESS	battery energy-storage system
MPC	model predictive control
PCC	point common coupling
CCT	critical clearing time
PV	photovoltaic
WT	wind turbine
SG	synchronous generator
SoC	state of charge
EPS	electric power system
DER	distributed energy resources
DFIG	doubly fed induction generator
PMSG	permanent magnet synchronous generator
MPPT	maximum power point tracking
CCPP	combined-cycle power plant
GT	gas turbine
HRSG	heat recovery steam generator
ST	steam turbine
AEA	adaptive energy-aware
UFLS	underfrequency load shedding
